# Switched “On” Transient Fluorescence Output from a Pulsed-Fuel Molecular Ratchet

**DOI:** 10.1021/jacs.3c11290

**Published:** 2023-12-04

**Authors:** Andrei S. Baluna, Marcel Dommaschk, Burkhard Groh, Salma Kassem, David A. Leigh, Daniel J. Tetlow, Dean Thomas, Loli Varela López

**Affiliations:** Department of Chemistry, University of Manchester, Oxford Road, Manchester, M13 9PL, U.K.

## Abstract

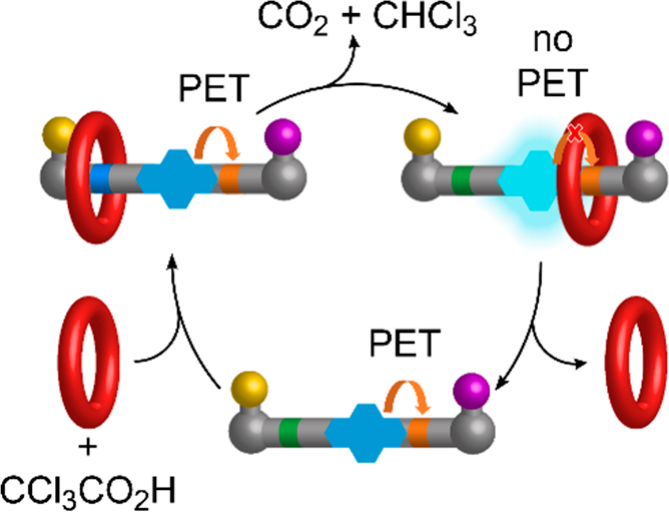

We report the synthesis and operation of a molecular energy ratchet that transports a crown ether from solution onto a thread, along the axle, over a fluorophore, and off the other end of the thread back into bulk solution, all in response to a single pulse of a chemical fuel (CCl_3_CO_2_H). The fluorophore is a pyrene residue whose fluorescence is normally prevented by photoinduced electron transfer (PET) to a nearby *N*-methyltriazolium group. However, crown ether binding to the *N*-methyltriazolium site inhibits the PET, switching on pyrene fluorescence under UV irradiation. Each pulse of fuel results in a single ratchet cycle of transient fluorescence (encompassing threading, transport to the *N*-methyltriazolium site, and then dethreading), with the onset of the fluorescent time period determined by the amount of fuel in each pulse and the end-point determined by the concentration of the reagents for the disulfide exchange reaction. The system provides a potential alternative signaling approach for artificial molecular machines that read symbols from sequence-encoded molecular tapes.

## Introduction

**Figure 1:**
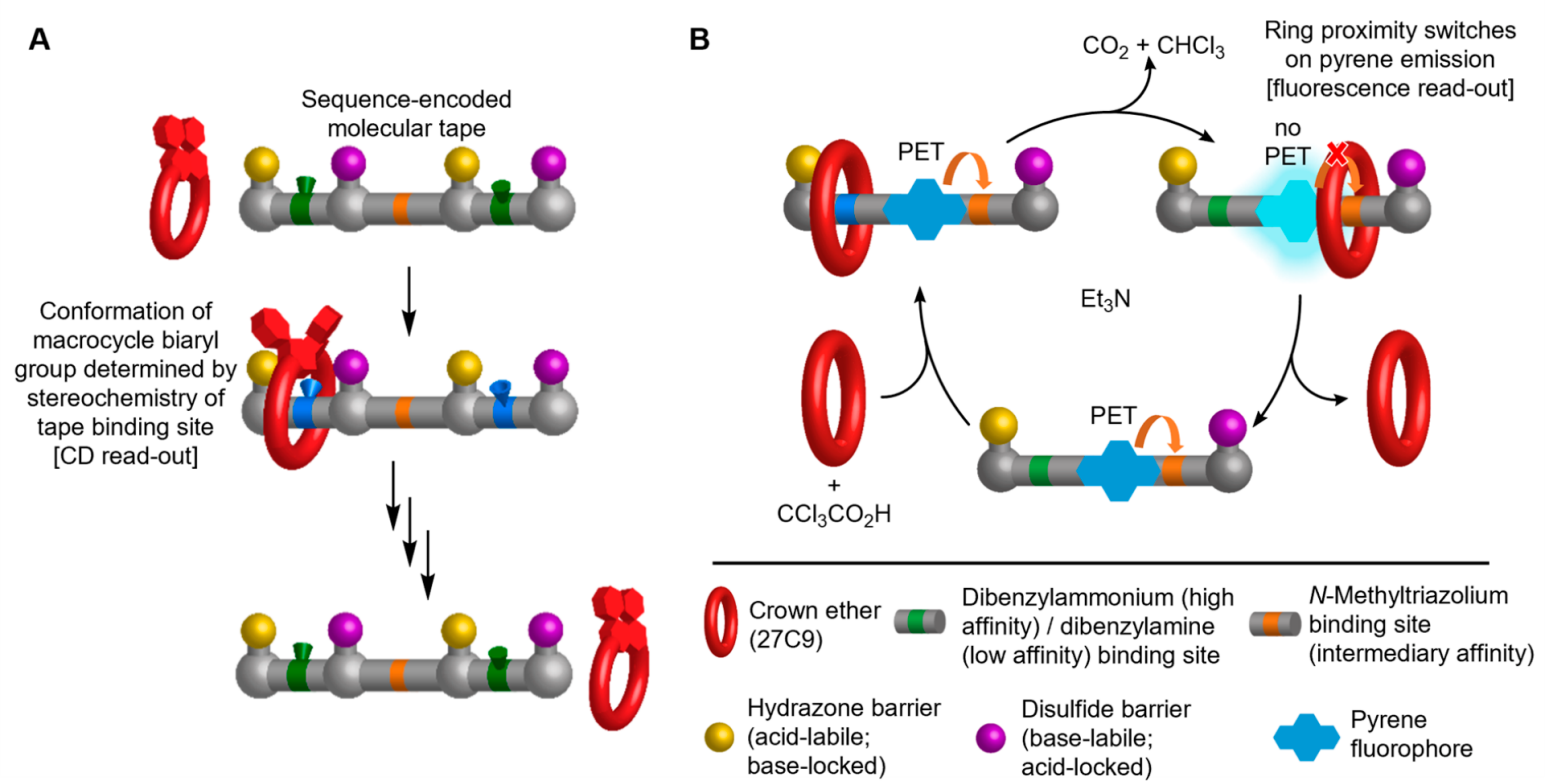
(A) Chiroptical readout of the sequence of stereochemistry encoded ‘symbols’ on a molecular tape through the directionally changing macrocycle position in a pulsed-fuel molecular ratchet (previous work).^43^ (B) Fluorescence readout of a fluorophore ‘symbol’ on a molecular tape through inhibition of PET by the directionally changing macrocycle position in a pulsed-fuel molecular ratchet. Operational cycle of a transiently fluorescent molecular ratchet in response to discrete pulses of CCl_3_CO_2_H (this work).

Biology uses chemically fueled ratcheting^1−7^ in numerous metabolic processes,^8^ including molecular-level information processing^9−11^ and transportation.^12^ Chemically powered ratcheting also forms the basis of a number of artificial molecular motors,^13−21^ pumps,^20,22−31^ and dissipative materials.^2,3,32−37^ A molecular energy ratchet^16,29,38−44^ was recently reported^43^ in which a crown ether (a “reading head” that responds to particular “symbols” encoded on a molecular tape) is pumped from solution onto a molecular strand by a pulse^16,29,45−53^ of a chemical fuel.^5^ Further fuel pulses transported the macrocycle along the tape before releasing it back to the bulk solution off the other end of the strand. During its directional transport the crown ether changes conformation according to the stereochemistry of binding sites (asymmetric substituted dibenzylammonium (*dba*-) and *N*-methyltriazolium (*mt*-) groups) encountered along the way.^43^ This allowed the sequence of stereochemical information programed into the molecular tape (i.e., the order the different binding sites are reached by the directionally transported crown ether) to be read out as a string of digits in a nondestructive manner through a changing circular dichroism response (Figure 1A).^43^

However, in the first generation^43^ tape-reading ratchet the *N*-methyltriazolium groups are achiral, so there is no stereochemistry for the crown ether to respond to at half of the tape binding sites. Here, we demonstrate a potential alternative output that could be used for artificial molecular machines that read information from molecular tapes: transient fluorescence.^54^

### Molecular Design

**Figure 2:**
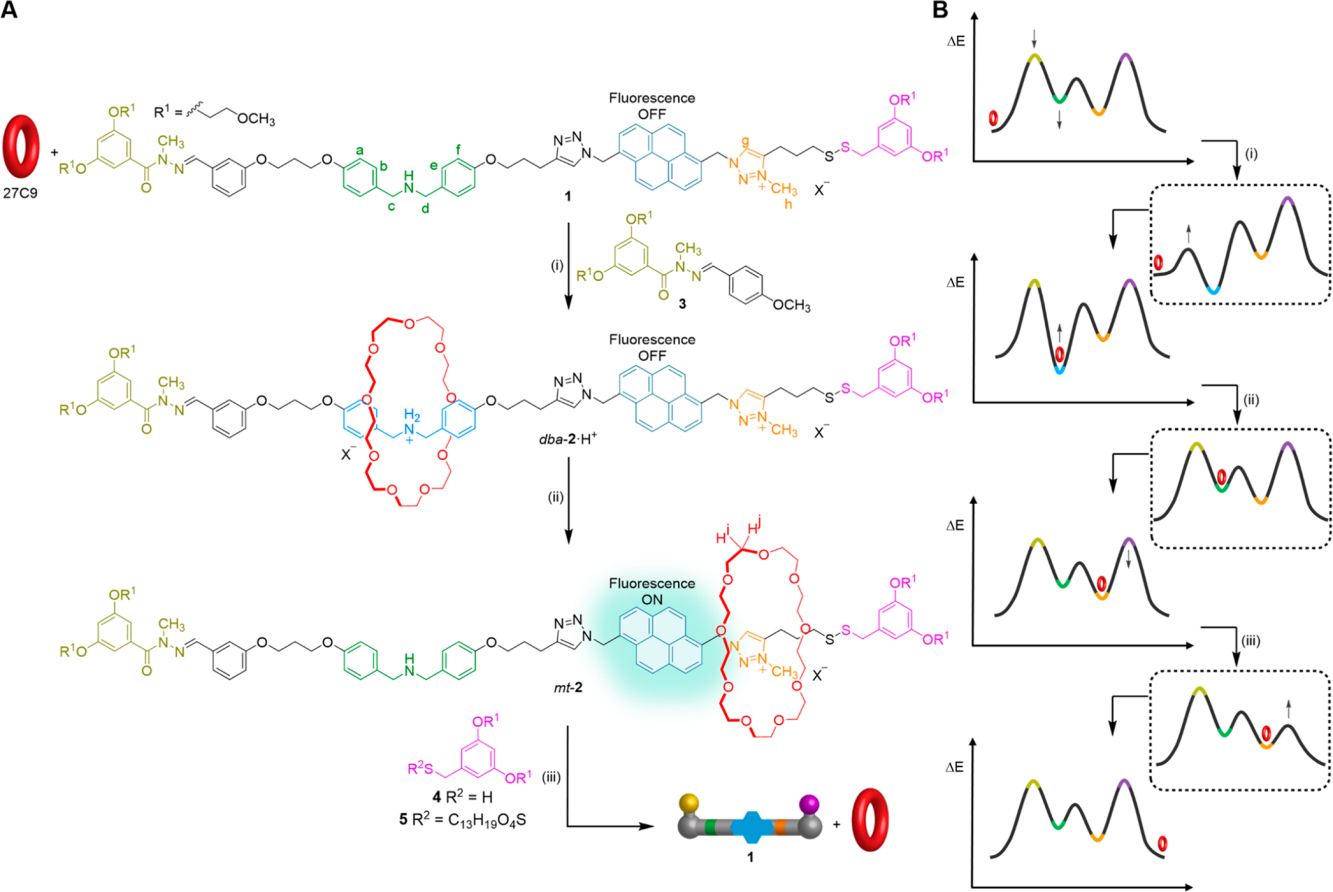
Stepwise operation of a transiently fluorescent molecular ratchet (**1** → **2** → **1**). (A) Reagents and conditions: (i) **1** (10 mM), CF_3_CO_2_H (5.0 equiv), PhNH_2_ (0.5 equiv), hydrazone **3** (3.0 equiv), 27C9 (5.0 equiv), CD_3_CN, RT, 5 d, 21%; (ii) *dba*-**2**·H^+^ (1.6 mM), Et_3_N (50 equiv), CD_3_CN, RT, 5 min, quantitative; (iii) thiol **4** (2.0 equiv), disulfide **5** (20 equiv), RT, 24 h, quantitative. X^–^ denotes various anions that change during ratchet operation. (B) Qualitative representations of the energy profiles of the individual machine states to illustrate how the ring is transported onto, along, and off the thread.

A key concept underpinning the new system (Figure 1B) is that the fluorescence of a pyrene residue on the tape should only be “switched on”^55^ when a threaded directionally transported crown ether binds to the *N*-methyltriazolium group adjacent to the fluorophore. Otherwise the pyrene fluorescence is quenched^56−65^ by photoinduced electron transfer (PET). To explore the feasibility of such a signaling process, we designed a model with a single digit (“1”) encoded in the molecular tape, as shown in Figures 1B and 2.

An energy ratchet switches between different potential energy surfaces to reverse the relative depths of pairs of energy minima and the relative heights of pairs of energy maxima (Figure 2B).^1,7^ This inexorably leads to statistically dictated directional transport without the position of the substrate needing to affect the kinetics of transport.^1,7^ In the model energy ratchet (Figure 2A), the molecular thread contains dibenzylamine (green)/dibenzylammonium (blue) and *N*-methyltriazolium (orange) binding sites for the crown ether. Bulky groups at each end restrict the passage of the ring onto and off of the thread. The bulky hydrazone group (yellow) is acid-labile but kinetically inert under basic conditions; the bulky disulfide (purple) is base-labile but kinetically inert at acidic pH. This enables back-and-forth switching of the pH to cause the crown ether to enter and leave the thread from opposite ends. The system completes one full operational cycle with a single pulse of trichloroacetic acid (CCl_3_CO_2_H), a “pulsed fuel”^16,29,43,45−53^ that undergoes base-catalyzed decarboxylation, forming CO_2_ and CHCl_3_ as the only waste products.^16,29,43,66−68^

In the presence of Et_3_N, the pulse of CCl_3_CO_2_H synchronizes the state of the molecules in the system by first changing the environment to an acidic pH (temporarily converting the Et_3_N present to the ammonium carboxylate salt).^16,43^ This promotes threading of the crown ether onto the protonated dibenzylammonium site past the labile hydrazone barrier. As the trichloroacetic acid decarboxylates, the pH of the solution becomes basic (the loss of acid liberating the Et_3_N originally present), and the ammonium group is deprotonated to an amine, which has a low affinity for crown ether binding.

Consequently, the macrocycle quickly relocates to the *N*-methyltriazolium binding site (the local energy minimum) before slowly dethreading over the disulfide barrier (rate-limited by disulfide exchange) back into the bulk solution, which is the global energy minimum state of the system at basic pH.

The final feature of the design is fluorescence readout of the crown ether position provided by incorporating a pyrene residue within the thread (Figure 2). The pyrene fluorescence is quenched by PET to the adjacent *N*-methyltriazolium group.^58−65^ However, when the crown ether binds to the *N*-methyltriazolium site, the PET is disrupted, and under UV-irradiation centered around 343 nm, the pyrene fluoresces. In this way, the intensity of any fluorescence output can be linked to the position of the macrocycle on the molecular thread.

## Results and Discussion

The thread (**1**; Figure 2) for the molecular ratcheting cycle was prepared in 13 steps from commercially available starting materials (Supporting Information section 3). With **1** in hand, we first carried out the energy ratchet reaction cycle stepwise (Figure 2A) to facilitate structural characterization of each of the intermediates and, in particular, to correlate the position of the macrocycle on the molecular strand with the fluorescent output.

### Stepwise Operation of the Molecular Ratchet

**Figure 3:**
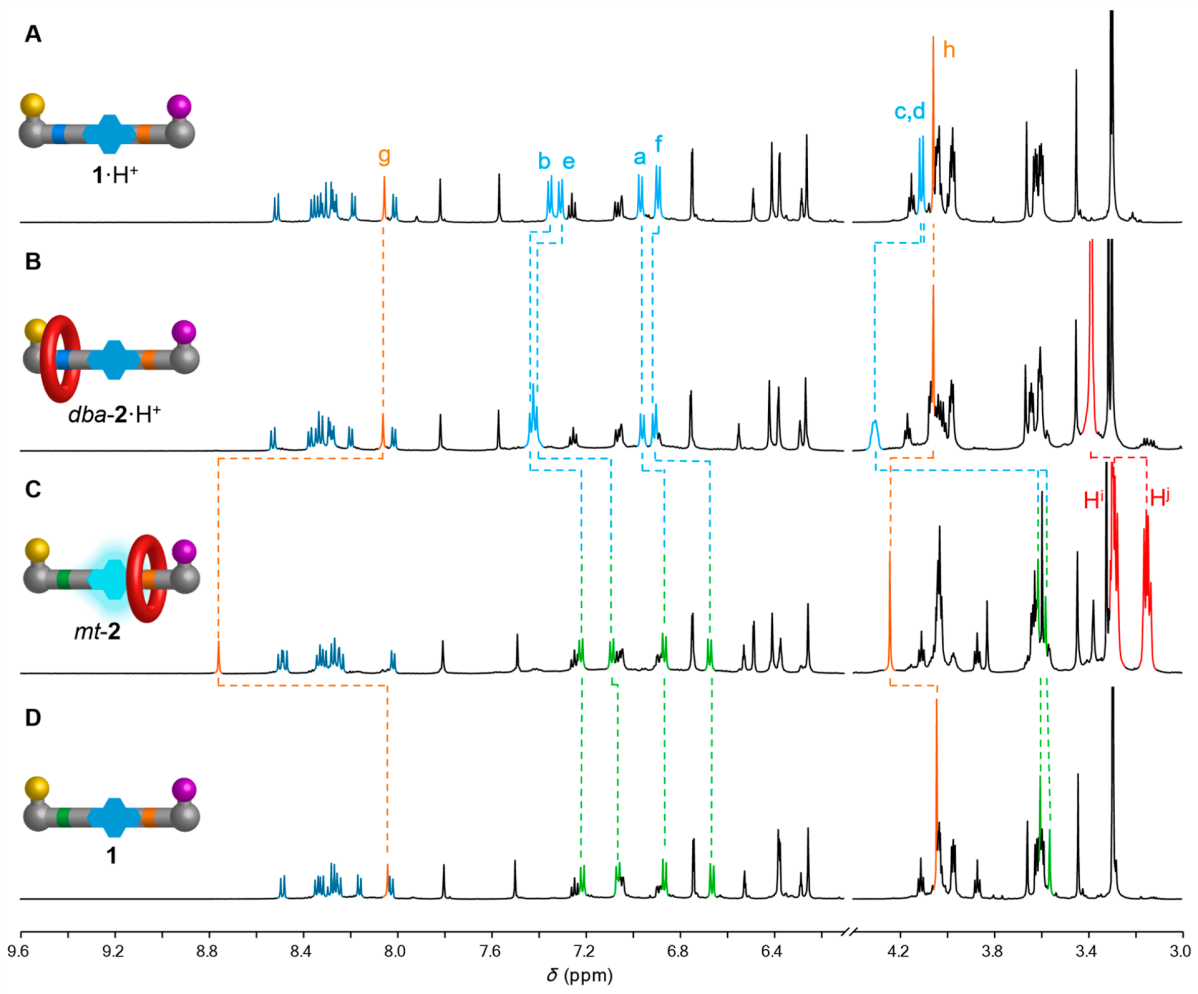
Partial ^1^H NMR (600 MHz, 298 K, CD_3_CN) of thread **1**/**1**·H^+^ and the rotaxane protonated and unprotonated co–conformers (*dba*-**2**·H^+^ and *mt*-**2**) formed during the ratchet cycle: (A) **1**·H^+^; (B) *dba*-**2**·H^+^; (C) *mt*-**2**; (D) **1**. The lettering corresponds to the proton labeling shown in Figure 2A.

**Figure 4:**
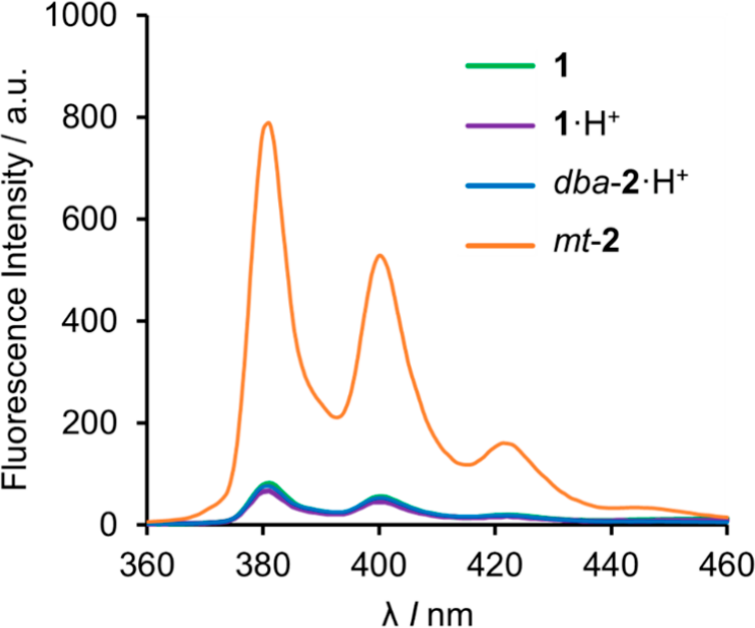
Fluorescence emission spectra (λ_ex_ = 343 nm, Δλ_ex_ = 5 nm, Δλ_em_ = 5 nm, *C* = 25 μM, 298 K) of **1** (green), **1**·H^+^ (purple), *dba*-**2**·H^+^ (blue), and *mt*-**2** (orange) in CH_3_CN.

Treatment of **1** and 27-crown-9 (27C9) with CF_3_CO_2_H (an analog of CCl_3_CO_2_H that does not spontaneously decarboxylate) smoothly afforded a [2]rotaxane (as evidenced by ^1^H NMR spectroscopy and mass spectrometry, see Supporting Information section 4.1) over the course of several days. The rate of threading was limited by slow exchange of the hydrazone barrier, which was easily improved by adding a catalytic amount of aniline.^69^ The ^1^H NMR spectrum of the rotaxane product (Figure 3B) showed substantial shifts in the resonances of protons associated with the dibenzylammonium group (H_b–e_) with respect to those in the protonated but unthreaded precursor, **1**·H^+^ (Figure 3A). This confirmed the macrocycle to be situated at the dibenzylammonium site in this form of the rotaxane, i.e., the structure to be *dba*-**2**·H^+^ (the italicized prefix indicates the position of the macrocycle on the axle). Photoirradiation of rotaxane *dba*-**2**·H^+^ at 343 nm showed a weak emission profile with the characteristic shape of pyrene fluorescence (Figure 4, blue line, and Supporting Information section 5.3).

To transport the macrocycle of the rotaxane from the *dba*-site to the *mt*-site, excess triethylamine (Et_3_N) was added to *dba*-**2**·H^+^ to deprotonate the dibenzylammonium group. ^1^H NMR spectroscopy of the resulting structure (Figure 3C) showed downfield shifts of 0.7 and 0.2 ppm for H_g_ and H_h_, respectively, compared to **1** (Figure 3D), indicating shuttling of the crown ether to the *N*-methyltriazolium group (i.e., co–conformation *mt*-**2**). In contrast to the protonated rotaxane (*dba*-**2**·H^+^) and the unprotonated but unthreaded axle (**1**), *mt*-**2** was brightly fluorescent when irradiated at 343 nm (Figure 4, orange line), demonstrating that crown ether binding to the *N*-methyltriazolium group switched on the fluorescence response of the adjacent pyrene fluorophore.

To induce the final step of the ratcheting cycle (dethreading of the macrocycle off the other end of the track), thiol **4** (2.0 equiv) and disulfide **5** (20 equiv) were added to the solution of *mt*-**2**. Dethreading occurred over the course of 3 h, leading to full recovery of **1** and 27C9 (Supporting Information section 4.2 and Figure S3).

### Pulsed-Fuel Operation of the Molecular Ratchet

**Figure 5:**
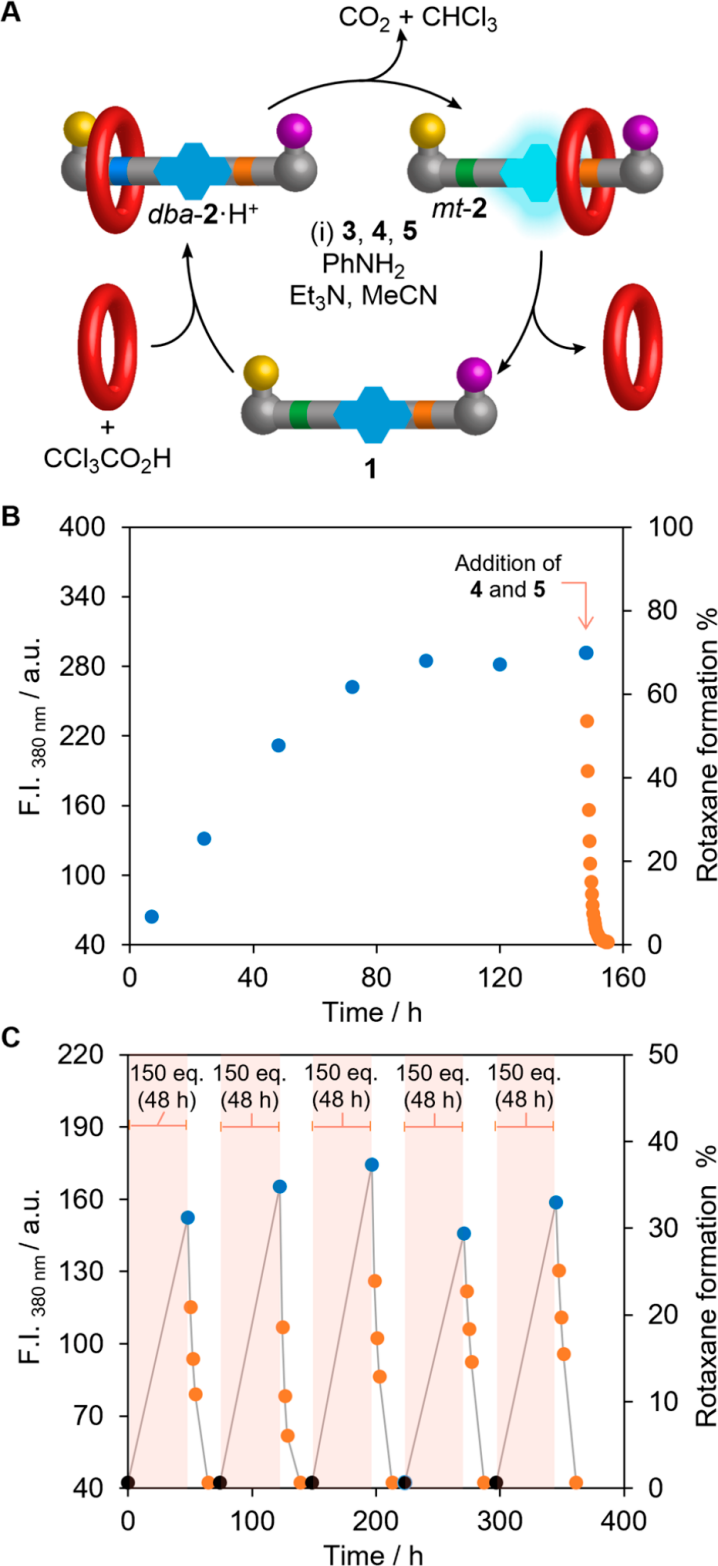
Pulsed-fuel operation of a transiently fluorescent molecular ratchet (**1** → **2** → **1**): (A) Operational cycle **1** → **2** → **1**. Reagents and conditions (representative values): **1** (1.3 mM), Et_3_N (50 equiv), PhNH_2_ (0.5 equiv), hydrazone **3** (3.0 equiv), 27C9 (20 equiv), thiol **4** (2.0 equiv except in part B where instead after 6 d 50 equiv was added to induce rapid dethreading), disulfide **5** (20 equiv except in part B where instead after 6 d 500 equiv was added to induce rapid dethreading), CCl_3_CO_2_H (200 equiv in part B; 150 equiv in part C), CD_3_CN. (B) Fluorescence intensity (normalized for concentration) measured during the operation of ratchet **1** with a single pulse of CCl_3_CO_2_H (200 equiv); **4** (50 equiv) and **5** (500 equiv) added after 6 d. (C) Fluorescence intensity (normalized for concentration) measured during repetitive operation of ratchet **1** over five cycles of CCl_3_CO_2_H (5 × 150 equiv). Data point color coding: black, collected before the addition of CCl_3_CO_2_H; blue, collected during or immediately after the consumption of fuel; orange, collected during dethreading after all fuel consumed. Data obtained from fluorescence intensity at λ_em max_ = 380 nm (λ_ex_ = 343 nm, Δλ_ex_ = 5 nm, Δλ_em_ = 5 nm).

Having demonstrated the stepwise operation of the transiently fluorescent energy ratchet, we initiated the same reaction cycle with a single pulse of CCl_3_CO_2_H (Figure 5A). Compound **1**, 27C9, Et_3_N, and the reagents required to exchange hydrazone and disulfide blocking groups at different pHs (**3**, **4**, and **5**), were mixed in CD_3_CN, and a pulse of CCl_3_CO_2_H was added (Figure 5A). Within 5 min, the formation of some *dba*-**2**·H^+^ was apparent by ^1^H NMR spectroscopy (see Supporting Information section 4.3 and Figure S5) and increased to its maximum value over the course of 6 d (days). Upon consumption of the fuel,^16^ the pH of the medium became basic and the presence of *mt*-**2** (again, evident from ^1^H NMR spectroscopy) corresponded with a substantial increase in fluorescence of the solution under irradiation at 343 nm. Over the course of a further 12 h, the magnitude of the fluorescence response decreased, accompanied by the concomitant formation of **1** and 27C9 (Supporting Information section 4.3).

### Fluorescence Changes during the Ratcheting Cycle

**1**, **1**·H^+^, *dba*-**2**·H^+^, and *mt*-**2** all have intense absorption bands centered around 343 nm, which do not overlap with absorption bands of any of the reagents used in the ratcheting operation. The extinction coefficients are similar irrespective of the presence of the macrocycle (see Supporting Information sections 5.1 and 5.4). The fluorescence emission spectra (Figure 4) show that under irradiation at 343 nm, *mt*-**2** has a fluorescence intensity ∼10× higher than that of the other states of the ratchet, confirming that macrocycle binding to the *N*-methyltriazolium site disrupts PET from the pyrene.

Fluorescence spectroscopy was then used to analyze both the threading and dethreading steps individually (Figure 5B). For the threading study, aliquots at different time points were collected from a pulsed-fuel reaction mixture, in the absence of **4** and **5** to inhibit dethreading, as the system becomes increasingly basic during decomposition of the CCl_3_CO_2_H. After 4 d, the threading process had reached an equilibrium distribution of ∼7:3 rotaxane (**2**):thread (**1**) (see Supporting Information section 5.6). Disulfide exchange was then initiated by adding a large excess of **4** and **5**, leading to rapid dethreading. The release of **1** and 27C9 was accompanied by a decrease in fluorescence intensity down to the original level of the starting materials.

As disulfide exchange is the rate-limiting step for dethreading of *mt*-**2**, the process that switches off fluorescence in the ratcheting cycle, the duration of fluorescence could be varied by changing the concentration of **4** and **5**. Similarly, the time available for the threading step and, following that, the onset of fluorescence varies according to the amount of fuel in each pulse (see Supporting Information section 5.7). Together, these two features offer a means of controlling the onset and end-point of transient fluorescence by the molecular ratchet.

The outcome and performance of the ratcheting cycle were reproducible over multiple pulses of fuel (Figure 5C). The use of more rapidly decomposing pulsed-fuel systems, such as CBr_3_CO_2_H^70^ or the use of polar solvents such as DMSO,^71^ could allow for shorter periods for the ratcheting cycle and correspondingly reduced times for reading “switch on” fluorescent symbols encoded on molecular tapes.

## Conclusions

The pulsed fuel mediated transformation of **1** → **2** → **1** is a model energy ratchet cycle that starts with the crown ether being pumped from solution onto the thread. The ring is then directionally transported along the track, where binding to a *N*-methyltriazolium group transiently switches on fluorescence (∼10× increase in intensity) from an adjacent irradiated pyrene fluorophore, before the ring is returned to bulk solution off the other end of the track. The movements of the molecules in the ensemble are synchronized, as the stage of the ratcheting cycle is linked to the pH of the environment. All of this occurs in response to a single pulse of CCl_3_CO_2_H. The amount of fuel used in the pulse and of the disulfide exchange reagents can be used to vary both the period of the ratcheting cycle and the duration of the transient fluorescence. This would correspond to the time taken to read each symbol on a molecular tape.

In principle, such a system could be used to read the presence or absence of fluorophores at different locations on the tape, each fluorophore only “switched on” for fluorescence output when the directionally transported macrocycle arrives at that position. Digits 0 and 1 could be encoded in sequence by the absence/presence of pyrene, but different fluorophores could also potentially be added (e.g., corresponding to the symbols 2, 3, etc.) to increase the base of digits for information storage. Intriguingly, the fluorophore switch-on system could even be combined with a stereochemistry encoded system^43^ to generate molecular tapes where one message could be read through fluorescence and a different message on the same tape could be read through circular dichroism. Such systems are under investigation in our laboratory.

## Supporting Information Available

Experimental procedures, synthesis and characterization data, NMR, MS, UV/vis, and fluorescence spectra (PDF)
