# Supplementary material for: Switched “On” Transient Fluorescence Output from a Pulsed-Fuel Molecular Ratchet
Source: J Am Chem Soc. 2023 Dec 4;145(49):27113–9. doi: 10.1021/jacs.3c11290 (PMC10722508; doi:10.1021/jacs.3c11290)
Supplement: Supplementary file 1 — ja3c11290_si_001.pdf [file ja3c11290_si_001.pdf]

# Supporting Information

## Switched ‘On’ Transient Fluorescence Output from a Pulsed-Fuel Molecular Ratchet

Andrei S. Baluna, Marcel Dommaschk, Burkhard Groh, Salma Kassem, David A. Leigh,*
Daniel J. Tetlow, Dean Thomas and Loli Varela López

Department of Chemistry, University of Manchester, Oxford Road, Manchester M13 9PL, UK

Correspondence to: david.leigh@manchester.ac.uk

# Table of Contents

1 Abbreviations ... 3

2 General Methods & Information ... 4

3 Experimental Section ... 5

3.1 Synthetic Schemes ... 5

3.2 Synthetic Procedures & Characterization Details ... 8

4 Operation of **1** ... 26

4.1 Stepwise Formation of *dba*-**2**·$H^+$ ... 26

4.2 Switching and Dethreading of *dba*-**2**·$H^+$ ... 29

4.3 Pulsed-Fuel Operation of **1** ... 33

5 Fluorescence Spectroscopy ... 36

5.1 UV-Vis and Fluorescence Emission Spectra of **1** ... 36

5.2 Fluorescence Emission Spectra of **1**·$H^+$ ... 37

5.3 Fluorescence Emission Spectra of *dba*-**2**·$H^+$ ... 37

5.4 UV-Vis and Fluorescence Emission Spectra of *mt*-**2** ... 38

5.5 Fluorescence Emission Spectra During the Switching of *mt*-**2** to *dba*-**2**·$H^+$ Under Transient Acidic Conditions. ... 39

5.6 Fluorescence Emission Spectra During the Stepwise Threading and Dethreading Processes ... 40

5.7 Fluorescence Emission Spectra During the Pulsed-Fuel Operation of **1**. ... 41

6 $^{1}H$ & $^{13}C$ NMR Spectra ... 43

7 References ... 65

# 1 Abbreviations

2-aldrithiol 2,2′-dipyridyl disulfide; 27C9 27-crown-9; Ac acetyl; ADMP azido-1,3-dimethylimidazolinium hexafluorophosphate; Boc *t*-butyl carbamate; Bu butyl; Bz benzoyl; CuAAC copper-catalysed azide-alkyne cycloaddition; DBA dibenzyl ammonium; DBU 1,8-diazabicyclo[5.4.0]undec-7-ene; DEAD diethyl azodicarboxylate; DMAP 4-(dimethylamino)pyridine; DMF *N*,*N*-dimethylformamide; DMSO dimethylsulfoxide; DPPA diphenylphosphoryl azide; EDTA ethylenediaminetetraacetic acid; ET electron transfer; EtOH ethanol; HRMS high resolution mass spectrometry; MeCN acetonitrile; MeOH methanol; Ms methanesulfonyl; NMR nuclear magnetic resonance; PET photoinduced electron transfer; PPTS *p*-toluenesulfonic acid pyridine salt; PTLC preparatory thin layer chromatography; rt room temperature; TBTA tris(benzyltriazolylmethyl)amine; Tf triflate/trifluoromethanesulfonate; THF tetrahydrofuran; TLC thin layer chromatography; Ts tosyl.

# 2 General Methods & Information

Unless stated otherwise, reagents were obtained from commercial sources and used without purification. Unless otherwise stated, all reactions were carried out in anhydrous solvents and under a $N_2$ or Ar atmosphere. Anhydrous THF (HPLC grade, Fischer Scientific), $CHCl_3$ (99.8+%, Fischer Scientific), $CH_2Cl_2$ (HPLC grade, Fischer Scientific), $CH_3CN$ (HPLC grade, Fischer Scientific), and toluene (>99%, Fischer Scientific) were obtained by passing the solvent through an activated alumina column on a Phoenix SDS (solvent drying system; JC Meyer Solvent Systems, CA, USA). DMF (peptide synthesis grade, Merck) was used unless otherwise stated. $^1H$ NMR spectra were recorded on a Bruker Avance III instrument with an Oxford AS600 magnet equipped with a cryoprobe [5mm CPDCH 13C-1H/D] (600 MHz) at 298 K. Chemical shifts are reported in parts per million (ppm) from high to low frequency using the residual solvent peak as the internal reference ($CD_3CN$ = 1.94 ppm, $(CD_3)_2SO$ = 2.50 ppm, $CDCl_3$ = 7.26 ppm). All $^1H$ resonances are reported to the nearest 0.01 ppm. The multiplicity of $^1H$ signals are indicated as: s = singlet; d = doublet; t = triplet; q = quartet, p = pentet; m = multiplet; br = broad; or combinations thereof. Coupling constants (*J*) are quoted in Hz and reported to the nearest 0.1 Hz. $^{13}C$ NMR spectra (151 MHz) were recorded on the same spectrometer at 298 K with the central resonance of the solvent peak as the internal reference ($CD_3CN$ = 1.32 ppm, $(CD_3)_2SO$ = 39.52 ppm, $CDCl_3$ = 77.16 ppm). All $^{13}C$ resonances are reported to the nearest 0.1 ppm in general, or to 0.01 ppm to aid in the differentiation of close but resolved signals. DEPT, COSY, HSQC and HMBC experiments (standard Bruker library, Topspin 3.6) were used to aid structural determination and spectral assignment. Flash column chromatography was carried out using Silica 60 Å (particle size 40–63 µm, Sigma Aldrich, UK) as the stationary phase. Analytical TLC was performed on precoated silica gel plates (0.25 mm thick, 60 F254, Merck, Germany) and visualized using both short and long-waved ultraviolet light in combination with standard laboratory stains (potassium permanganate or ceric ammonium molybdate). Size-exclusion chromatography was carried out under gravity using a neutral, porous styrene divinylbenzene resin (1% crosslinked linked, Bio-Rad, Bio-Beads, S-X1) as stationary phase and $CH_2Cl_2$ as an eluent. Anion-exchange was carried out by passing a concentrated solution of the organic salt through a bed of anion-exchange resin (amberlyst A-26, Sigma-Aldrich) with MeCN as an eluent. The appropriate anion exchange resin is obtained by eluting the resin (OH form) with a concentrated aqueous solution of $KPF_6$, then washed with deionised water till neutral, followed by anhydrous MeOH and finally, MeCN. UV/vis spectra were acquired on a Varian Cary 100 and fluorescence spectra were acquired on a Varian Cary Eclipse instrument. Low resolution ESI mass spectrometry was performed with a Thermo Scientific LCQ Fleet Ion Trap Mass Spectrometer or an Agilent Technologies 1200 LC system with either an Agilent 6130 single quadrupole MS detector or an Advion Expression LCMS single quadrupole MS detector. High-resolution mass spectrometry was carried out by the Mass Spectrometry Service, Department of Chemistry, the University of Manchester. Compounds **S1**[1], **S4**[2], **S6**[3], **S10**[4], **S11**[5], **S17**[6] and 27-crown-9 (27C9)[7] were synthesised according to literature procedures.

# 3 Experimental Section

## 3.1 Synthetic Schemes

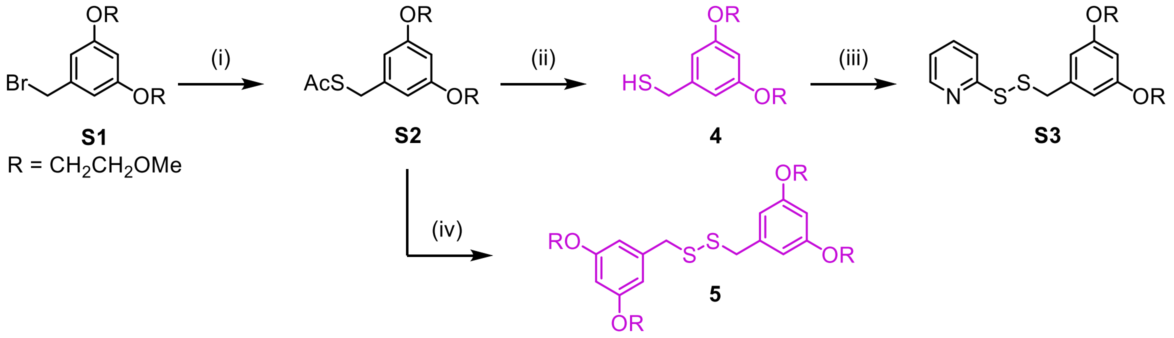


**Scheme S1** – Synthesis of thiol **4**, disulfide **5** and **S3**. Reagents and conditions: (i) **S1** (1.0 equiv.), KSAc (1.2 equiv.), MeCN, 85 °C, 16 h, 99%. (ii) **S2** (1.0 equiv.), $N_2H_4 \cdot H_2O$ (2.0 equiv.), MeCN, 0 °C to rt, 5 h, 78%. (iii) **4** (1.0 equiv.), 2,2′-dipyridyldisulfide (4.0 equiv.), $Et_3N$ (2.0 equiv.), $CH_2Cl_2$, rt, 17 h, 65%. (iv) **S2** (1.0 equiv.), pyrrolidine (2.0 equiv.), $I_2$ (0.1 equiv.), $CH_2Cl_2$, rt, 27 h, 68%.

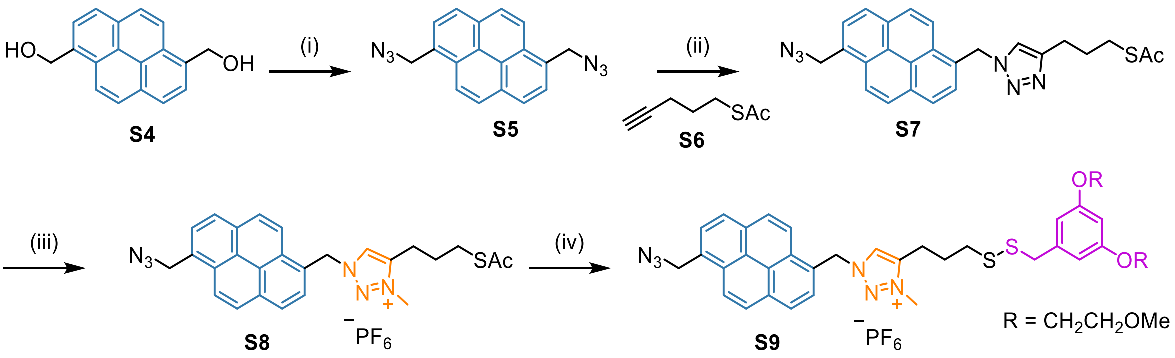


**Scheme S2** – Synthesis of triazolium **S9.** Reagents and conditions: (i) **S4** (1.0 equiv.), DPPA (2.3 equiv.), DBU (2.0 equiv.), DMF, 0 °C to rt, 2 h, 83%. (ii) **S5** (2.0 equiv.), **S6** (1.0 equiv.), $[Cu(MeCN)_4]PF_6$ (20 mol%), TBTA (25 mol%), $CH_2Cl_2/{}^tBuOH$ (4:1, *v*/*v*), rt, 22 h, 69%. (iii) **S7** (1.0 equiv.), $Me_3OBF_4$ (2.0 equiv.), 0 °C to rt, 4 h, $CH_2Cl_2$, *then* anion-exchange (Amberlyst A26/ $PF_6^-$, MeCN), 52%. (iv) **S8** (1.0 equiv.), pyrrolidine (2.0 equiv.), $CH_2Cl_2$, rt, 2 h, *then* **S3** (1.1 equiv.), rt, 20 h, 18%.

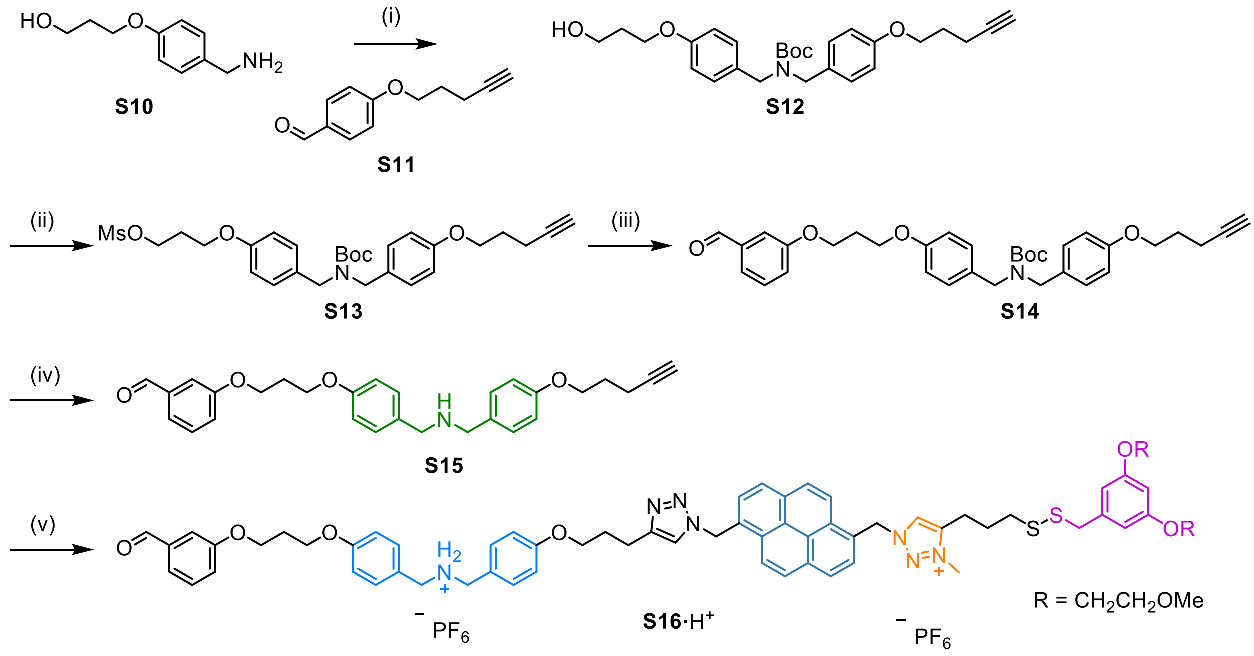


**Scheme S3** – Synthesis of **S16**·H$^+$. Reagents and conditions: (i) **S10** (1.0 equiv.), **S11** (1.2 equiv.), $MgSO_4$, MeOH/THF (7.3:1, *v*/*v*), rt, 16 h, *then* $NaBH_4$ (4.2 equiv.), 0 °C to rt, 1 h, *then* Boc anhydride (1.1 equiv.), $CH_2Cl_2$, rt, 20 h, 87%. (ii) **S12** (1.0 equiv.), MsCl (1.6 equiv.), $Et_3N$ (1.1 equiv.), $CH_2Cl_2$, 0 °C, 10 min, *then* rt, 1 h, 95%. (iii) **S13** (1.0 equiv.), 3-hydroxybenzaldehyde (1.1 equiv.), $K_2CO_3$ (3.0 equiv.), MeCN, 85 °C, 3 d, 64%. (iv) **S14** (1.0 equiv.), $CF_3CO_2H/CH_2Cl_2$ (1:4, *v*/*v*), rt, 30 min, *then* wash with sat. aq. $K_2CO_3$, quant. (v) **S9** (1.0 equiv.), **S15** (1.3 equiv.), $[Cu(MeCN)_4]PF_6$ (20 mol%), TBTA (25 mol%), $CH_2Cl_2$/$^t$BuOH (4:1, *v*/*v*), rt, 20 h, *then* anion-exchange (Amberlyst A26/ $PF_6^-$, MeCN), 52%.

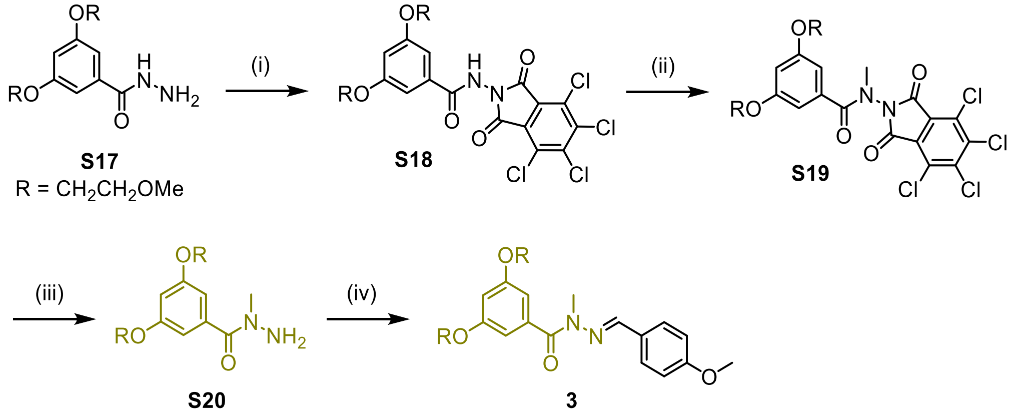


**Scheme S4** – Synthesis of hydrazide **3**. Reagents and conditions: (i) **S17** (1.0 equiv.), tetrachlorophtalic anhydride (1.0 equiv.), toluene, 120 °C, 19 h, 98%. (ii) **S18** (1.0 equiv.), DEAD (1.5 equiv.), $PPh_3$ (1.5 equiv.), MeOH (1.5 equiv.), THF, 0 °C to rt, 2 h, 95%. (iii) **S19** (1.0 equiv.), $N_2H_4 \cdot H_2O$ (1.3 equiv.), EtOH, 70 °C, 24 h, 86%. (iv) **S20** (1.0 equiv.), 4-methoxybenzaldehyde (8.5 equiv.), $CF_3CO_2H$ (1.0 equiv.), $CH_2Cl_2$, rt, 10 min, 80%.

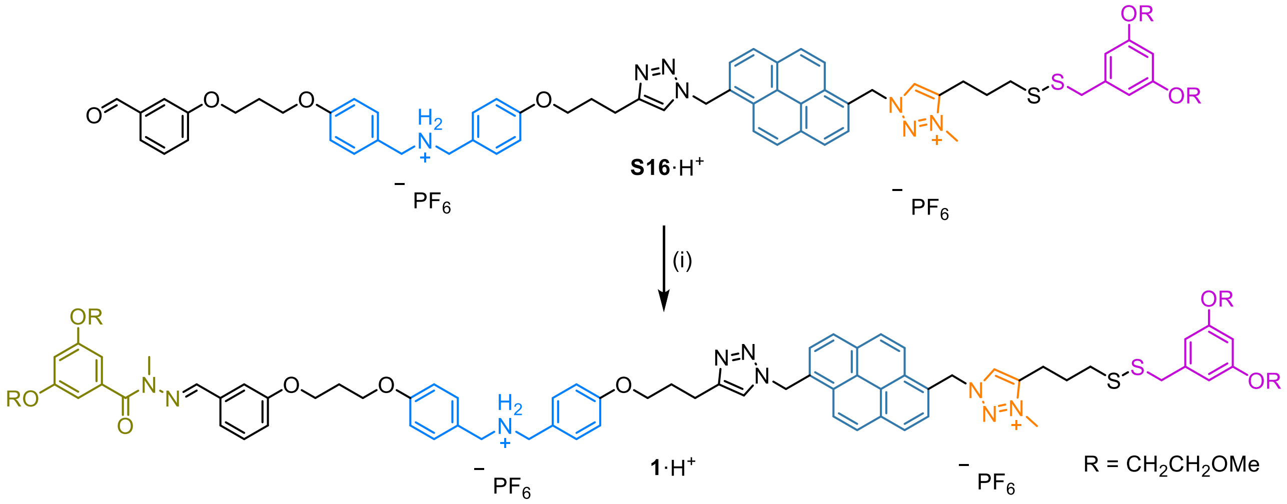


**Scheme S5** – Synthesis of **1**·H$^+$. Reagents and conditions: (i) **S16**·H$^+$ (1.0 equiv.), **S20** (3.0 equiv.), $CF_3CO_2H$ (2.0 equiv.), $CD_3CN$, rt, *then* anion-exchange (Amberlyst A26/ $PF_6^-$, MeCN), 76%.

## 3.2 Synthetic Procedures & Characterization Details

### S2

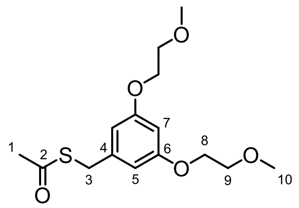


A suspension of **S1** (1.62 g, 5.08 mmol) and potassium thioacetate (700 mg, 6.13 mmol, 1.2 equiv.) in MeCN (50 mL, 0.1 M) was refluxed for 16 h. The solvent was removed under reduced pressure and the resulting crude residue was purified by flash column chromatography ($SiO_2$, 2.5% MeOH in $CH_2Cl_2$) to afford **S2** (1.59 g, 5.06 mmol, 99%) as a yellow oil.

**$^1$H NMR** (600 MHz, 298 K, Chloroform-*d*) δ 6.46 (d, *J* = 2.2 Hz, 2H, $H_5$), 6.40 (t, *J* = 2.2 Hz, 1H, $H_7$), 4.10 – 4.05 (m, 4H, $H_8$), 4.03 (s, 2H, $H_3$), 3.74 – 3.70 (m, 4H, $H_9$), 3.44 (s, 6H, $H_{10}$), 2.34 (s, 3H, $H_1$).

**$^{13}$C NMR** (151 MHz, 298 K, Chloroform-*d*) δ 195.2 ($C_2$), 160.1 ($C_6$), 139.8 ($C_4$), 107.8 ($C_5$), 100.6 ($C_7$), 71.1 ($C_9$), 67.4 ($C_8$), 59.4 ($C_{10}$), 33.7 ($C_3$), 30.4 ($C_1$).

**HRMS** (ASAP$^+$): Calculated for $C_{15}H_{23}O_5S$: 315.1261 $[M+H]^+$, found 315.1259.

**4**

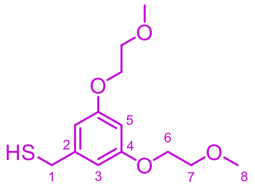


To a solution of **S2** (1.59 g, 5.06 mmol) in degassed MeCN (50 mL, 0.1 M) at 0 °C under Ar, hydrazine monohydrate (0.49 mL, 10.10 mmol, 2.0 equiv.) was added dropwise over 25 minutes *via* syringe. The reaction mixture was warmed to room temperature and stirred for 5 hours. The solvent was removed under reduced pressure and the crude residue was purified by flash column chromatography ($SiO_2$, 15% $Et_2O$ in $CHCl_3$) to afford **4** (1.08 g, 3.96 mmol, 78%) as a colorless oil.

**$^1$H NMR** (600 MHz, 298 K, Chloroform-*d*) δ 6.51 (d, $J$ = 2.2 Hz, 2H, $H_3$), 6.40 (t, $J$ = 2.2 Hz, 1H, $H_5$), 4.11 – 4.06 (m, 4H, $H_5$), 3.76 – 3.70 (m, 4H, $H_7$), 3.65 (d, $J$ = 7.6 Hz, 2H, $H_1$), 3.44 (s, 6H, $H_8$), 1.73 (t, $J$ = 7.6 Hz, 1H, $H_{SH}$).

**$^{13}$C NMR** (151 MHz, 298 K, Chloroform-*d*) δ 160.2 ($C_4$), 143.4 ($C_2$), 107.1 ($C_2$), 100.4 ($C_5$), 71.1 ($C_7$), 67.4 ($C_6$), 59.4 ($C_8$), 29.4 ($C_1$).

**HRMS** ($ASAP^+$): Calculated for $C_{13}H_{21}O_4S$: 271.1155 $[M+H]^+$, found 271.1152.

**S3**

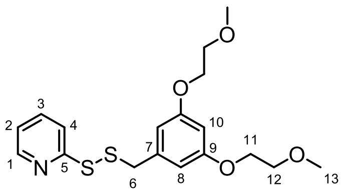


To a solution of **4** (300 mg, 1.10 mmol) and 2,2′-dipyridyldisulfide (969 mg, 4.40 mmol, 4.0 equiv.) in dry degassed $CH_2Cl_2$ (2 mL), $Et_3N$ (0.31 mL, 2.22 mmol, 2.0 equiv.) was added, and the reaction mixture was stirred for 17 hours at room temperature. The reaction was quenched by the addition of saturated aqueous $NH_4Cl$ (10 mL) and the biphasic mixture was extracted with $CH_2Cl_2$ (3 × 20 mL). The combined organic layers were washed with brine, dried over $MgSO_4$, filtered, and the solvent was removed under reduced pressure. The crude residue was purified by flash column chromatography ($SiO_2$, 40% EtOAc in petroleum ether) to afford **S3** (275 mg, 0.72 mmol, 65%) as a colorless oil.

**$^1$H NMR** (600 MHz, 298 K, Chloroform-*d*) δ 8.42 (dd, *J* = 4.8, 1.8 Hz, 1H, $H_1$), 7.56 – 7.49 (m, 2H, $H_{3,4}$), 7.03 (ddd, *J* = 7.0, 4.8, 1.3 Hz, 1H, $H_2$), 6.48 (d, *J* = 2.3 Hz, 2H, $H_8$), 6.38 (t, *J* = 2.3 Hz, 1H, $H_{10}$), 4.04 – 3.98 (m, 4H, $H_{11}$), 3.93 (s, 2H, $H_6$), 3.77 – 3.68 (m, 4H, $H_{12}$), 3.44 (s, 6H, $H_{13}$).

**$^{13}$C NMR** (151 MHz, 298 K, Chloroform-*d*) δ 160.3 ($C_1$), 160.0 ($C_9$), 149.6 ($C_5$), 138.8 ($C_7$), 136.9 ($C_3$), 120.6 ($C_4$), 119.6 ($C_2$), 108.2 ($C_8$), 101.1 ($C_{10}$), 71.1 ($C_{12}$), 67.4 ($C_{11}$), 59.4 ($C_{13}$), 44.3 ($C_6$).

**HRMS** ($ESI^+$): Calculated for $C_{18}H_{23}O_4NS_2Na$: 404.0961 $[M+Na]^+$, found 404.0955.

**5**

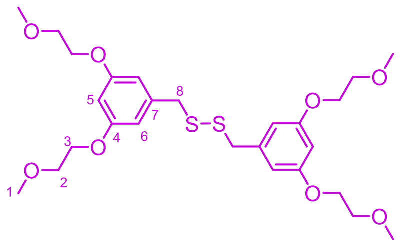


To a solution of **S2** (480 mg, 1.53 mmol) and iodine (40 mg, 0.16 mmol, 0.1 equiv.) in $CH_2Cl_2$ (4 mL), pyrrolidine (0.25 mL, 3.06 mmol, 2.0 equiv.) was added, and the reaction was stirred for 27 hours at room temperature under an atmosphere of air. The resultant mixture was evaporated onto $SiO_2$ under reduced pressure and purified by flash column chromatography ($SiO_2$, 15% $Et_2O$ in $CH_2Cl_2$) to give **5** (280 mg, 0.52 mmol, 68%) as a colorless oil.

**$^1$H NMR** (600 MHz, 298 K, Chloroform-*d*) δ 6.46 – 6.41 (m, 6H, $H_{5,6}$), 4.12 – 4.05 (m, 8H, $H_3$), 3.75 – 3.71 (m, 8H, $H_2$), 3.59 – 3.56 (m, 4H, $H_8$), 3.47 – 3.42 (m, 12H, $H_1$).

**$^{13}$C NMR** (151 MHz, 298 K, Chloroform-*d*) δ 160.0 ($C_4$), 139.6 ($C_7$), 108.3 ($C_6$), 100.9 ($C_5$), 71.1 ($C_2$), 67.4 ($C_3$), 59.4 ($C_1$), 41.8 ($C_8$).

**HRMS** (ESI$^+$): Calculated for $C_{26}H_{38}O_8S_2Na$: 565.1900 $[M+Na]^+$, found 565.1905.

**S5**

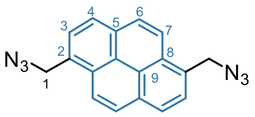

To a solution of **S4**[2] (360 mg, 1.37 mmol) and DBU (0.41 mL, 2.75 mmol, 2.0 equiv.) in dry DMF (40 mL) at 0 °C under Ar, DPPA (0.68 mL, 3.16 mmol, 2.3 equiv.) was added dropwise over 10 minutes. The reaction mixture was warmed to room temperature and stirred for 2 hours. The mixture was concentrated under reduced pressure to ~ 5 mL and purified by flash chromatography ($SiO_2$, $CH_2Cl_2$) to give **S5** (355 mg, 1.14 mmol, 83%) as a yellow solid.

**m.p.** 160 – 162 °C.

**$^1$H NMR** (600 MHz, 298 K, Chloroform-*d*) δ 8.32 (d, *J* = 9.1 Hz, 2H, $H_7$), 8.24 (d, *J* = 7.8 Hz, 2H, $H_4$), 8.20 (d, *J* = 9.1 Hz, 2H, $H_6$), 8.03 (d, *J* = 7.8 Hz, 2H, $H_3$), 5.07 (s, 4H, $H_1$).

**$^{13}$C NMR** (151 MHz, 298 K, Chloroform-*d*) δ 131.5 ($C_2$), 129.6 ($C_5$), 129.2 ($C_8$), 128.5 ($C_6$), 128.0 ($C_3$), 125.5 ($C_4$), 125.3 ($C_9$), 123.3 ($C_7$), 53.3 ($C_1$).

**HRMS** (APCI$^+$): Calculated for $C_{18}H_{13}N_6$: 313.1196 $[M+H]^+$, found 313.1195.

## S7

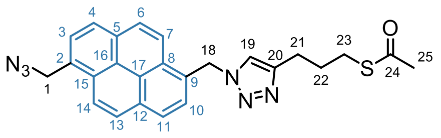


To a solution of **S5** (260 mg, 0.83 mmol, 2.0 equiv.) in degassed $CH_2Cl_2/^tBuOH$ (4:1, 25 mL), $[Cu(MeCN)_4]PF_6$ (30 mg, 0.08 mmol, 0.2 equiv.) and TBTA (53 mg, 0.10 mmol, 0.25 equiv.) were added. **S6**[3] (60 mg, 0.42 mmol) dissolved in degassed $CH_2Cl_2/^tBuOH$ (5 mL, 4:1, *v*/*v*) was added dropwise *via* syringe at room temperature, over 20 hours. After complete addition of **S6**, the reaction was stirred for 2 hours at room temperature and then quenched with saturated aqueous $Na_4EDTA$ (30 mL, pH = 7). The aqueous phase was extracted with $CH_2Cl_2$ (3 × 15 mL) and the organic layers were combined, washed with brine, dried over $MgSO_4$, filtered and the solvent was removed under reduced pressure. The crude residue was purified by flash chromatography ($SiO_2$, 3% MeOH in $CH_2Cl_2$) to give **S7** (130 mg, 0.29 mmol, 69%) as a yellow solid.

**m.p.** 126 – 128 °C.

**$^1$H NMR** (600 MHz, 298 K, Chloroform-*d*) δ 8.35 (d, *J* = 9.2 Hz, 1H, $H_{14}$), 8.26 (d, *J* = 9.2 Hz, 1H, $H_7$), 8.24 (d, *J* = 7.8 Hz, 1H, $H_{11}$), 8.23 (d, *J* = 7.8 Hz, 1H, $H_4$), 8.21 (d, *J* = 9.2 Hz, 1H, $H_{13}$), 8.16 (d, *J* = 9.2 Hz, 1H, $H_6$), 8.04 (d, *J* = 7.8 Hz, 1H, $H_3$), 8.00 (d, *J* = 7.8 Hz, 1H, $H_{10}$), 7.14 (s, 1H, $H_{19}$), 6.26 (s, 2H, $H_{18}$), 5.07 (s, 2H, $H_1$), 2.82 (t, *J* = 7.2 Hz, 2H, $H_{23}$), 2.71 (t, *J* = 7.5 Hz, 2H, $H_{21}$), 2.23 (s, 3H, $H_{25}$), 1.86 (p, *J* = 7.4 Hz, 2H, $H_{22}$).

**$^{13}$C NMR** (151 MHz, 298 K, Chloroform-*d*) δ 196.0 ($C_{24}$), 147.1 ($C_{20}$), 132.0 ($C_{12}$), 131.4 ($C_5$), 129.7 ($C_{15}$), 129.5 ($C_8$), 129.5 ($C_2$), 129.3 ($C_6$), 128.4 ($C_{13}$), 128.2 ($C_3$), 128.2 ($C_{10}$), 127.4 ($C_9$), 125.80 ($C_{11}$), 125.75 ($C_4$), 125.3 ($C_{17}$), 125.1 ($C_{16}$), 121.8 ($C_{14}$), 122.5 ($C_7$), 121.4 ($C_{19}$), 51.2 ($C_1$), 52.9 ($C_{18}$), 30.7 ($C_{25}$), 29.1 ($C_{22}$), 28.4 ($C_{23}$), 24.5 ($C_{21}$).

**HRMS** ($ESI^+$): Calculated for $C_{25}H_{23}ON_6S$: 455.1649 $[M+H]^+$, found 451.1641.

## S8

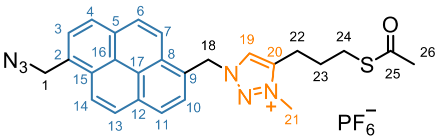


To a solution of **S7** (130 mg, 0.29 mmol) in dry degassed $CH_2Cl_2$ (5 mL) at 0 °C, $Me_3OBF_4$ (86 mg, 0.58 mmol, 2.0 equiv.) was added in one portion and the reaction was stirred at 0 °C for 2 hours. The mixture was then allowed to warm to room temperature and stirred for further 2 hours, before being quenched with $CF_3CO_2H$ (110 µL, 1.45 mmol, 5.0 equiv.). The solvent was removed under reduced pressure and the crude residue was purified by flash column chromatography ($SiO_2$, 6% MeOH in $CH_2Cl_2$) and then by anion-exchange chromatography (Amberlyst A26 $PF_6^-$, MeCN) to give **S8** (90 mg, 0.15 mmol, 52%) as a yellow sticky solid.

**$^1$H NMR** (600 MHz, 298 K, Acetonitrile-$d_3$) δ 8.39 (d, $J$ = 9.1 Hz, 1H, $H_{14}$), 8.33 (d, $J$ = 7.9 Hz, 1H, $H_{11}$), 8.30 (d, $J$ = 7.8 Hz, 1H, $H_4$), 8.26 – 8.22 (m, 3H, $H_{6,7,13}$), 8.15 (d, $J$ = 7.9 Hz, 1H, $H_{10}$), 8.12 (d, $J$ = 7.8 Hz, 1H, $H_3$), 8.11 (s, 1H, $H_{19}$), 6.38 (s, 2H, $H_{18}$), 5.11 (s, 2H, $H_1$), 4.06 (s, 3H, $H_{21}$), 2.81 (t, $J$ = 7.4 Hz, 2H, $H_{24}$), 2.75 (t, $J$ = 7.4 Hz, 2H, $H_{22}$), 2.21 (s, 3H, $H_{26}$), 1.83 (p, $J$ = 7.4 Hz, 2H, $H_{23}$).

**$^{13}$C NMR** (151 MHz, 298 K, Acetonitrile-$d_3$) δ 196.5 ($C_{25}$), 145.0 ($C_{20}$), 131.2 ($C_{12}$), 131.9 ($C_5$), 131.3 ($C_{15}$), 130.7 ($C_8$), 130.16 ($C_{10}$), 130.11 ($C_{16}$), 130.0 ($C_6$), 129.4 ($C_3$), 128.97 ($C_{19}$), 128.94 ($C_{13}$), 126.8 ($C_4$), 126.7 ($C_{11}$), 125.8 ($C_{17}$), 125.6 ($C_9$), 125.3 ($C_2$), 125.2 ($C_{14}$), 121.4 ($C_7$), 55.9 ($C_{18}$), 51.2 ($C_1$), 38.5 ($C_{21}$), 30.8 ($C_{26}$), 28.2 ($C_{24}$), 27.5 ($C_{23}$), 22.7 ($C_{22}$).

**HRMS** (ESI$^+$): Calculated for $C_{26}H_{25}ON_6S$: 469.1805 $[M]^+$, found 469.1799.

**S9**

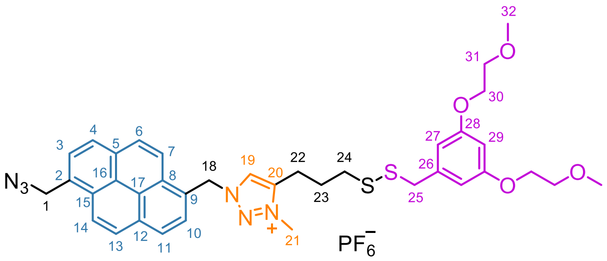


To a solution of **S8** (90 mg, 0.15 mmol) in degassed $CH_2Cl_2$ (1 mL), pyrrolidine (25 µL, 0.30 mmol, 2.0 equiv.) was added and the reaction was stirred at room temperature for 2 hours. A solution of **S3** (60 mg, 0.16 mmol, 1.1 equiv.) in degassed $CH_2Cl_2$ (0.25 mL) was added and the reaction was stirred for 20 hours at room temperature. The solvent was removed under reduced pressure and the crude residue was purified by flash column chromatography ($SiO_2$, 6% MeOH in $CH_2Cl_2$) and, then by anion-exchange chromatography (Amberlyst A26 $PF_6^-$, MeCN) to give **S9** (23 mg, 0.027 mmol, 18%) as an orange solid.

**m.p.** 80 – 82 °C.

**$^1$H NMR** (600 MHz, 298 K, Acetonitrile-$d_3$) δ 8.46 (d, $J$ = 9.2 Hz, 1H, $H_{14}$), 8.39 (d, $J$ = 7.8 Hz, 1H, $H_{11}$), 8.36 (d, $J$ = 7.8 Hz, 1H, $H_4$), 8.34 – 8.28 (m, 3H, $H_{6,7,13}$), 8.21 (d, $J$ = 7.8 Hz, 1H, $H_{10}$), 8.16 (d, $J$ = 7.8 Hz, 1H, $H_3$), 8.07 (s, 1H, $H_{19}$), 6.43 (s, 2H, $H_{18}$), 6.40 (d, $J$ = 2.3 Hz, 2H, $H_{27}$), 6.30 (t, $J$ = 2.3 Hz, 1H, $H_{29}$), 5.15 (s, 2H, $H_1$), 4.06 (s, 3H, $H_{21}$), 4.02 – 3.95 (m, 4H, $H_{30}$), 3.69 (s, 2H, $H_{25}$), 3.63 – 3.59 (m, 4H, $H_{31}$), 3.31 (s, 6H, $H_{32}$), 2.72 (t, $J$ = 7.4 Hz, 2H, $H_{22}$), 2.45 (t, $J$ = 7.3 Hz, 2H, $H_{24}$), 1.84 (p, $J$ = 7.3 Hz, 2H, $H_{23}$).

**$^{13}$C NMR** (151 MHz, 298 K, Acetonitrile-$d_3$) δ 161.0 ($C_{28}$), 145.1 ($C_{20}$), 141.1 ($C_{26}$), 131.3 ($C_{12}$), 131.9 ($C_5$), 131.5 ($C_{15}$), 130.8 ($C_8$), 130.3 ($C_{10}$), 130.2 ($C_{16}$), 130.1 ($C_6$), 129.5 ($C_3$), 129.0 ($C_{19}$), 128.9 ($C_{13}$), 126.9 ($C_4$), 126.7 ($C_{11}$), 125.9 ($C_{17}$), 125.7 ($C_9$), 125.4 ($C_2$), 125.4 ($C_{14}$), 121.5 ($C_7$), 108.9 ($C_{27}$), 100.8 ($C_{29}$), 71.6 ($C_{31}$), 68.3 ($C_{30}$), 59.0 ($C_{32}$), 56.0 ($C_{18}$), 51.2 ($C_1$), 41.4 ($C_{25}$), 38.5 ($C_{21}$), 37.0 ($C_{24}$), 26.6 ($C_{23}$), 22.4 ($C_{22}$).

**HRMS** (ESI$^+$): Calculated for $C_{36}H_{41}O_4N_6S_2$: 697.2626 $[M]^+$, found 697.2617.

## S12

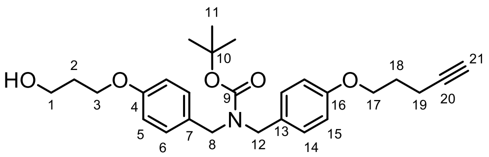


To a solution of **S10**[4] (2.30 g, 12.7 mmol) and **S11**[5] (2.87 g, 15.2 mmol, 1.2 equiv.) in MeOH (110 mL) and dry THF (15 mL) at room temperature, $MgSO_4$ (anhydrous, *excess*) was added, and the suspension was stirred for 16 hours. The reaction mixture was cooled to 0 °C and $NaBH_4$ (2.02 g, 53.4 mmol, 4.2 eq.) was added portion-wise over 20 minutes. The reaction was stirred for 45 minutes at 0 °C, followed by 25 minutes at room temperature, before being quenched with distilled water (5 mL). The solid particles were filtered off through Celite™ and the cake was washed with $CH_2Cl_2$ (2 × 50 mL). The combined organic phases were dried over $Na_2SO_4$, filtered, and the solvent was removed under reduced pressure. The crude residue was dissolved in dry $CH_2Cl_2$ (100 mL), to which Boc anhydride (2.0 M in THF, 7.0 mL, 14.0 mmol, 1.1 equiv.) was added and the reaction was stirred for 20 hours at room temperature. The resultant mixture was evaporated onto $SiO_2$ under reduced pressure and purified by flash column chromatography ($SiO_2$, 30% EtOAc in petroleum ether) to give **S12** (4.99 g, 11.0 mmol, 87%) as a colorless oil.

**Note*:** The molecule exists as a mixture of rotamers at room temperature, due to restricted rotation around the C-N bonds.

**$^1$H NMR** (600 MHz, 298 K, Chloroform-*d*) δ 7.14 (s, 2H, $H_{6,14}$), 7.10 (s, 2H, $H_{6,14}$), 6.87 – 6.85 (m, 4H, $H_{5,15}$), 4.32 (s, 2H, $H_8$), 4.24 (s, 2H, $H_{12}$), 4.12 (t, $J$ = 5.9 Hz, 2H, $H_3$), 4.06 (t, $J$ = 6.1 Hz, 2H, $H_{17}$), 3.87 (q, $J$ = 5.7 Hz, 2H, $H_1$), 2.41 (td, $J$ = 7.0, 2.6 Hz, 2H, $H_{19}$), 2.05 (p, $J$ = 5.9 Hz, 2H, $H_2$), 2.00 (p, $J$ = 6.8 Hz, 2H, $H_{18}$), 1.97 (t, $J$ = 2.6 Hz, 1H, $H_{21}$), 1.88 – 1.81 (m, 1H, $H_{OH}$), 1.50 (s, 9H, $H_{11}$).

**$^{13}$C NMR** (151 MHz, 298 K, Chloroform-*d*) δ 158.3 ($C_{16}$), 158.1 ($C_4$), 156.1 ($C_9$), 130.5 ($C_{7,13}$), 130.3 ($C_{7,13}$), 129.5 ($C_{6,14}$), 128.9 ($C_{6,14}$), 114.64 ($C_{5,15}$), 114.61 ($C_{5,15}$), 81.6 ($C_{20}$), 80.1 ($C_{10}$), 69.0 ($C_{21}$), 66.3 ($C_{17}$), 66.0 ($C_3$), 60.7 ($C_1$), 48.5 ($C_{8,12}$), 48.2 ($C_{8,12}$), 32.1 ($C_2$), 28.6 ($C_{11}$), 28.3 ($C_{18}$), 15.3 ($C_{19}$).

**HRMS** (ESI$^+$): Calculated for $C_{27}H_{35}O_5NNa$: 476.2407 $[M+Na]^+$, found 476.2394.

**S13**

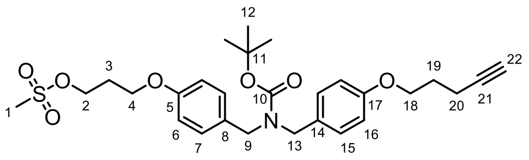


To a solution of **S12** (2.73 g, 6.03 mmol) in dry $CH_2Cl_2$ (20 mL) at 0 °C, MsCl (0.75 mL, 9.64 mmol, 1.6 equiv.) and triethylamine (0.92 mL, 6.63 mmol, 1.1 equiv.) were added, and the reaction was stirred for 10 minutes at 0 °C, followed by 1 hour at room temperature. Water (15 mL) was added, and the biphasic mixture was extracted with $CH_2Cl_2$ (3 × 25 mL). The combined organic layers were washed with brine, dried over $Na_2SO_4$, filtered and the solvent was removed under reduced pressure to give **S13** (3.05 g, 5.74 mmol, 95%) as a colorless oil which was used without further purification.

**Note*:** The molecule exists as a mixture of rotamers at room temperature, due to restricted rotation around the C-N bonds.

**$^1$H NMR** (600 MHz, 298 K, Chloroform-*d*) δ 7.15 (s, 2H, $H_{7,15}$), 7.11 (s, 2H, $H_{7,15}$), 6.88 – 6.83 (m, 4H, $H_{6,16}$), 4.45 (t, *J* = 6.1 Hz, 2H, $H_2$), 4.32 (s, 2H, $H_9$), 4.24 (s, 2H, $H_{13}$), 4.11 – 4.04 (m, 4H, $H_{4,18}$), 3.00 (s, 3H, $H_1$), 2.41 (td, *J* = 7.0, 2.6 Hz, 2H, $H_{20}$), 2.23 (p, *J* = 5.9 Hz, 2H, $H_3$), 2.00 (p, *J* = 6.6 Hz, 2H, $H_{19}$), 1.97 (t, *J* = 2.6 Hz, 1H, $H_{22}$), 1.50 (s, 9H, $H_{12}$).

**$^{13}$C NMR** (151 MHz, 298 K, Chloroform-*d*) δ 158.3 ($C_{17}$), 157.9 ($C_5$), 156.1 ($C_{10}$), 130.8 ($C_{8,14}$), 130.3 ($C_{8,14}$), 129.6 ($C_{7,15}$), 129.0 ($C_{7,15}$), 114.7 ($C_{6,16}$), 114.6 ($C_{6,16}$), 81.6 ($C_{21}$), 80.1 ($C_{11}$), 69.0 ($C_{22}$), 66.9 ($C_2$), 66.3 ($C_{18}$), 61.4 ($C_4$), 48.5 ($C_{9,13}$), 48.2 ($C_{9,13}$), 37.4 ($C_1$), 29.3 ($C_3$), 28.6 ($C_{12}$), 28.3 ($C_{19}$), 15.3 ($C_{20}$).

**HRMS** (ESI$^+$): Calculated for $C_{28}H_{38}O_7NS$: 532.2363 $[M+H]^+$, found 532.2364.

## S14

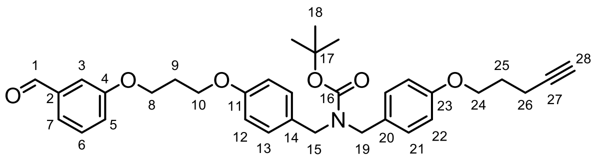


To a solution of **S13** (2.72 g, 5.12 mmol) and 3-hydroxybenzaldehyde (687 mg, 5.63 mmol, 1.1 equiv.) in MeCN (50 mL), $K_2CO_3$ (2.10 g, 15.2 mmol, 3.0 equiv.) was added, and the reaction mixture was heated at reflux for 3 days. The reaction mixture was cooled to room temperature and quenched with distilled water (50 mL). The product was extracted with $CH_2Cl_2$ (3 × 50 mL) and the combined organic layers were washed with brine, dried over $Na_2SO_4$, filtered, and the solvent was removed under reduced pressure. The resultant residue was purified by flash column chromatography ($SiO_2$, 20% EtOAc in petroleum ether) to give **S14** (1.82 g, 3.26 mmol, 64%) as a colorless oil.

**Note*:** The molecule exists as a mixture of rotamers at room temperature, due to restricted rotation around the C-N bonds.

**$^1$H NMR** (600 MHz, 298 K, Chloroform-*d*) δ 9.97 (s, 1H, $H_1$), 7.47 – 7.43 (m, 2H, $H_{6,7}$), 7.42 (d, *J* = 1.8 Hz, 1H, $H_3$), 7.19 (dt, *J* = 7.1, 2.3 Hz, 1H, $H_5$), 7.14 (s, 2H, $H_{13/21}$), 7.10 (s, 2H, $H_{21/13}$), 6.89 – 6.84 (m, 4H, $H_{12,22}$), 4.32 – 4.20 (m, 6H, $H_{8,15,19}$), 4.17 (t, *J* = 6.0 Hz, 2H, $H_{10}$), 4.06 (t, *J* = 6.1 Hz, 2H, $H_{24}$), 2.41 (td, *J* = 7.0, 2.6 Hz, 2H, $H_{26}$), 2.29 (p, *J* = 6.0 Hz, 2H, $H_9$), 2.00 (p, *J* = 6.5 Hz, 2H, $H_{25}$), 1.97 (t, *J* = 2.6 Hz, 1H, $H_{28}$), 1.50 (s, 9H, $H_{18}$).

**$^{13}$C NMR** (151 MHz, 298 K, Chloroform-*d*) δ 192.3 ($C_1$), 159.6 ($C_4$), 158.3 ($C_{23}$), 158.2 ($C_{11}$), 156.1 ($C_{16}$), 138.0 ($C_2$), 130.4 ($C_{14,20}$), 130.3 ($C_{14,20}$), 130.2 ($C_6$), 129.5 ($C_{13/21}$), 128.9 ($C_{21/13}$), 123.7 ($C_7$), 122.0 ($C_5$), 114.65 ($C_{12/22}$), 114.62 ($C_{22/12}$), 111.0 ($C_3$), 81.6 ($C_{27}$), 80.1 ($C_{17}$), 69.0 ($C_{28}$), 66.3 ($C_{24}$), 64.9 ($C_8$), 64.4 ($C_{10}$), 48.4 ($C_{15/19}$), 48.1 ($C_{19/15}$), 29.4 ($C_9$), 28.6 ($C_{18}$), 28.3 ($C_{25}$), 15.3 ($C_{26}$).

**HRMS** (APCI$^+$): Calculated for $C_{34}H_{40}O_6N$: 558.2850 $[M+H]^+$, found 558.2852.

## S15

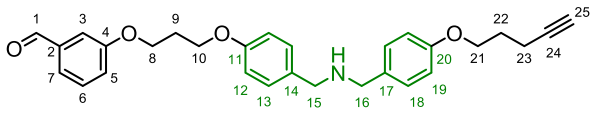


**S14** (19.0 mg, 34 µmol) was dissolved in $CF_3CO_2H/CH_2Cl_2$ (1 mL, 1:4, *v*/*v*) and the reaction mixture was stirred for 30 minutes at room temperature. The solution was quenched with sat. aq. $K_2CO_3$ (2 mL). The organic layer was separated, and the solvent was removed under reduced pressure to give **S15** (15.6 mg, 34 µmol, quant.) as a colorless oil which was used in the next step without further purification.

**$^1$H NMR** (600 MHz, 298 K, Chloroform-*d*) δ 9.97 (s, 1H, $H_1$), 7.47 – 7.42 (m, 2H, $H_{6,7}$), 7.41 (dd, *J* = 2.5, 1.2 Hz, 1H, $H_3$), 7.26 – 7.22 (m, 4H, $H_{13,18}$), 7.18 (dt, *J* = 7.2, 2.5 Hz, 1H, $H_5$), 6.89 – 6.85 (m, 4H, $H_{12,19}$), 4.23 (t, *J* = 6.1 Hz, 2H, $H_8$), 4.16 (t, *J* = 6.1 Hz, 2H, $H_{10}$), 4.05 (t, *J* = 6.4 Hz, 2H, $H_{21}$), 3.73 – 3.71 (m, 4H, $H_{15,16}$), 2.40 (td, *J* = 6.5, 2.7 Hz, 2H, $H_{23}$), 2.28 (p, *J* = 6.1 Hz, 2H, $H_9$), 1.99 (p, *J* = 6.5 Hz, 2H, $H_{22}$), 1.97 (t, *J* = 2.7 Hz, 1H, $H_{25}$).

**$^{13}$C NMR** (151 MHz, 298 K, Chloroform-*d*) δ 192.3 ($C_1$), 159.6 ($C_4$), 158.1 ($C_{20}$), 158.0 ($C_{11}$), 137.9 ($C_2$), 132.4 (br, $C_{14,17}$), 130.2 ($C_6$), 129.62 ($C_{13/18}$), 129.59 ($C_{18/13}$), 123.6 ($C_7$), 122.0 ($C_5$), 114.5 ($C_{12/19}$), 114.5 ($C_{19/12}$), 113.0 ($C_3$), 83.7 ($C_{24}$), 69.0 ($C_{25}$), 66.3 ($C_{21}$), 64.9 ($C_8$), 64.3 ($C_{10}$), 52.3 (br, $C_{15/16}$), 29.3 ($C_9$), 28.3 ($C_{22}$), 15.3 ($C_{23}$).

**HRMS** (APCI$^+$): Calculated for $C_{29}H_{32}O_4N$: 458.2326 $[M+H]^+$, found 458.2326.

**S16**·H$^+$

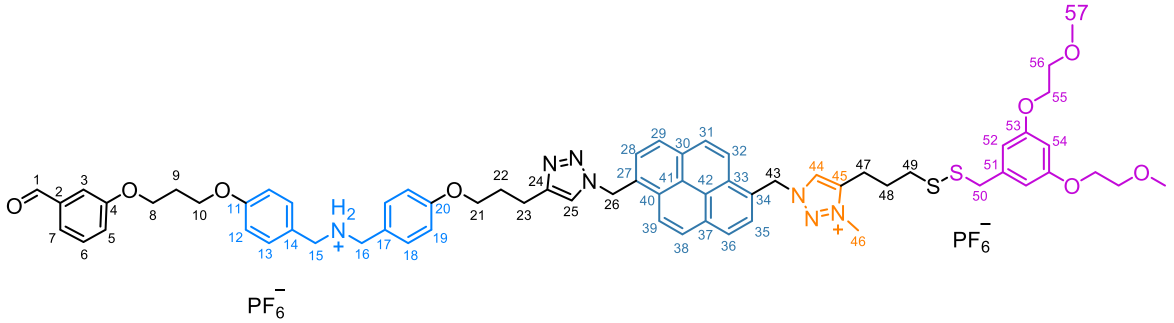

To a degassed solution of **S15** (15.6 mg, 34 μmol, 1.3 equiv.) in $CH_2Cl_2$/$^t$BuOH (4:1, 0.6 mL) under Ar was added $[Cu(MeCN)_4]PF_6$ (2.5 mg, 6 μmol, 0.2 equiv.) and TBTA (1.7 mg, 7 μmol, 0.25 equiv.). **S9** (23.0 mg, 27 μmol) dissolved in degassed $CH_2Cl_2$/$^t$BuOH (4:1, 0.4 mL) was added to the reaction mixture and stirred for 20 hours at room temperature. The reaction was quenched by the addition of saturated aqueous $Na_4$EDTA (2 mL, pH = 7). The biphasic mixture was extracted with $CH_2Cl_2$ (3 × 5 mL). The combined organic layers were washed with brine and dried over $MgSO_4$. The solvent was removed under reduced pressure and the crude residue was purified by size-exclusion chromatography (S-X1, $CH_2Cl_2$), and then by anion-exchange chromatography (Amberlyst A26 $PF_6^-$, MeCN) to give **S16**·H$^+$ (20.3 mg, 14 μmol, 52%), as a yellow crystalline solid.

**m.p.** 99 – 101 °C.

**$^1$H NMR** (600 MHz, 298 K, Acetonitrile-$d_3$) δ 9.92 (s, 1H, $H_1$), 8.48 (d, $J$ = 9.2 Hz, 1H, $H_{39}$), 8.33 (d, $J$ = 7.9 Hz, 1H, $H_{36}$), 8.31 (d, $J$ = 7.9 Hz, 1H, $H_{29}$), 8.29 (d, $J$ = 9.2 Hz, 1H, $H_{32}$), 8.25 (d, $J$ = 6.7 Hz, 1H, $H_{31}$), 8.24 (d, $J$ = 6.9 Hz, 1H, $H_{38}$), 8.17 (d, $J$ = 7.9 Hz, 1H, $H_{35}$), 8.06 (s, 1H, $H_{44}$), 8.00 (d, $J$ = 7.8 Hz, 1H, $H_{28}$), 7.60 (s, 1H, $H_{25}$), 7.49 – 7.46 (m, 2H, $H_{6,7}$), 7.40 – 7.38 (m, 1H, $H_3$), 7.35 (d, $J$ = 8.4 Hz, 2H, $H_{13}$), 7.30 (d, $J$ = 8.3 Hz, 2H, $H_{18}$), 7.22 (dt, $J$ = 6.6, 2.7 Hz, 1H, $H_5$), 6.97 (d, $J$ = 8.4 Hz, 2H, $H_{12}$), 6.87 (d, $J$ = 8.2 Hz, 2H, $H_{19}$), 6.40 (s, 2H, $H_{43}$), 6.37 (d, $J$ = 2.3 Hz, 2H, $H_{52}$), 6.28 (t, $J$ = 2.3 Hz, 1H, $H_{54}$), 6.25 (s, 2H, $H_{26}$), 4.22 (t, $J$ = 6.1 Hz, 2H, $H_8$), 4.18 (t, $J$ = 6.2 Hz, 2H, $H_{10}$), 4.14 – 4.08 (m, 4H, $H_{15,16}$), 4.06 (s, 3H, $H_{46}$), 4.01 – 3.93 (m, 6H, $H_{21,55}$), 3.66 (s, 2H, $H_{50}$), 3.63 – 3.57 (m, 4H, $H_{56}$), 3.30 (s, 6H, $H_{57}$), 2.77 (t, $J$ = 7.6 Hz, 2H, $H_{23}$), 2.72 (t, $J$ = 7.6 Hz, 2H, $H_{47}$), 2.44 (t, $J$ = 7.0 Hz, 2H, $H_{49}$), 2.23 (p, $J$ = 6.2 Hz, 2H, $H_9$), 2.01 (p, $J$ = 6.6 Hz, 2H, $H_{22}$), 1.83 (p, $J$ = 7.3 Hz, 2H, $H_{48}$).

**$^{13}$C NMR** (151 MHz, 298 K, Acetonitrile-$d_3$) δ 191.4 ($C_1$), 160.9 ($C_{53}$), 160.9 ($C_{11}$), 160.9 ($C_{20}$), 160.4 ($C_4$), 148.3 ($C_{24}$), 145.1 ($C_{45}$), 141.1 ($C_{51}$), 139.0 ($C_2$), 131.1 ($C_{37}$), 132.80 ($C_{13}$), 132.74 ($C_{18}$), 132.0 ($C_{30}$), 131.3 ($C_6$), 131.0 ($C_{40}$), 130.7 ($C_{33}$), 130.3 ($C_{35}$), 130.0 ($C_{31}$), 129.9 ($C_{41}$), 129.2 ($C_{38}$), 129.1 ($C_{28}$), 128.9 ($C_{44}$), 127.1 ($C_{29}$), 126.8 ($C_{36}$), 126.0 ($C_{42}$), 125.6 ($C_{34}$), 125.3 ($C_{27}$), 124.8 ($C_{39}$), 121.7 ($C_7$), 121.6 ($C_{32}$), 121.4 ($C_{14}$), 121.2 ($C_{17}$), 121.0 ($C_{25}$), 122.3 ($C_5$), 115.8 ($C_{12}$), 115.7 ($C_{19}$), 114.24 ($C_3$), 108.9 ($C_{52}$), 100.8 ($C_{54}$), 71.6 ($C_{56}$), 68.3 ($C_{55}$), 68.0 ($C_{21}$), 65.6 ($C_8$), 65.4 ($C_{10}$), 59.0 ($C_{57}$), 55.9 ($C_{43}$), 52.5 ($C_{26}$), 51.6 ($C_{15,16}$), 41.4 ($C_{50}$), 38.5 ($C_{46}$), 37.0 ($C_{49}$), 29.7 ($C_9$), 29.6 ($C_{22}$), 26.6 ($C_{48}$), 22.6 ($C_{23}$), 22.4 ($C_{47}$).

**HRMS** (ESI$^+$): Calculated for $C_{66}H_{72}O_6N_7S_2$: 1154.4878 [M]$^+$, found 1154.4861.

**S18**

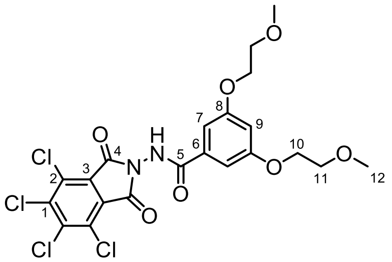


A suspension of **S17** (1.00 g, 3.52 mmol) and tetrachlorophthalic anhydride (1.01 g, 3.53 mmol, 1.0 equiv.) in toluene (50 mL) was heated at 120 °C for 19 hours. The reaction was cooled to room temperature and the solvent was removed under reduced pressure. The crude residue was purified by flash column chromatography ($SiO_2$, 2% MeOH in $CH_2Cl_2$) to afford **S18** (1.90 g, 3.44 mmol, 98%) as an off-white solid.

**m.p.** 190 – 192 °C.

**$^1$H NMR** (600 MHz, 298 K, DMSO-$d_6$) δ 11.44 (s, 1H, $H_{NH}$), 7.13 (d, $J$ = 2.3 Hz, 2H, $H_7$), 6.83 (t, $J$ = 2.3 Hz, 1H, $H_9$), 4.19 – 4.14 (m, 4H, $H_{10}$), 3.70 – 3.65 (m, 4H, $H_{11}$), 3.32 (s, 6H, $H_{12}$).

**$^{13}$C NMR** (151 MHz, 298 K, DMSO-$d_6$) δ 164.8 ($C_5$), 161.0 ($C_4$), 159.8 ($C_8$), 139.4 ($C_1$), 132.2 ($C_6$), 129.0 ($C_3$), 126.2 ($C_2$), 106.3 ($C_7$), 105.7 ($C_9$), 70.2 ($C_{11}$), 67.4 ($C_{10}$), 58.2 ($C_{12}$).

**HRMS** (ASAP$^+$): Calculated for $C_{21}H_{19}O_7N_2Cl_4$: 550.9941 $[M+H]^+$, found 550.9945.

## S19

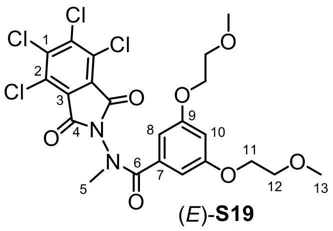

($E$)-**S19**

To a suspension of **S18** (550 mg, 1.00 mmol) and triphenylphosphine (394 mg, 1.50 mmol, 1.5 equiv.) in dry degassed THF (15 mL) at 0 °C under Ar, diethyl azodicarboxylate (0.24 mL, 1.53 mmol, 1.5 equiv.) was added and the reaction was stirred for 10 minutes. MeOH (0.06 mL, 1.49 mmol, 1.5 equiv.) was added dropwise, and the reaction mixture was stirred for 2 hours at room temperature. The reaction was quenched by the addition of sat. aq. $NaHCO_3$ (10 mL) and the biphasic mixture was extracted with $CH_2Cl_2$ (3 × 15 mL). The combined organic layers were washed with brine, dried over $Na_2SO_4$, filtered and the solvent was removed under reduced pressure. The crude residue was purified by flash column chromatography ($SiO_2$, 10% $Et_2O$ in $CH_2Cl_2$) to afford **S19** (538 mg, 0.95 mmol, 95%) as a viscous yellow oil.

**Note**: Two sets of signals have been observed with $^1$H NMR both in $CD_3CN$ and DMSO-$d_6$ ascribed to the occurrence of two amide bond rotamers (*E*)-**S19** (*major*) and (*Z*)-**S19** (*minor*) in slow exchange (298 K) and in an unequal ratio of approx. 3:1 ($CD_3CN$) and 4:1. (DMSO-$d_6$). Signals reported below correspond to the major isomer.

**$^1$H NMR** (600 MHz, 298 K, Acetonitrile-$d_3$) 6.48 (s, 2H, $H_8$), 6.45 (s, 1H, $H_{10}$), 3.97 (s, 4H, $H_{11}$), 3.60 (s, 4H, $H_{12}$), 3.30 (s, 9H, $H_{5,13}$).

**$^{13}$C NMR** (151 MHz, 298 K, Acetonitrile-$d_3$) δ 172.8 ($C_6$), 161.5 ($C_4$), 160.8 ($C_9$), 141.4 ($C_7$), 136.3 ($C_1$), 130.8 ($C_3$), 126.8 ($C_2$), 105.7 ($C_8$), 105.0 ($C_{10}$), 71.4 ($C_{12}$), 68.5 ($C_{11}$), 59.0 ($C_{13}$), 35.9 ($C_5$).

**HRMS** (ASAP$^-$): Calculated for $C_{22}H_{20}O_7N_2Cl_4$: 564.0019 $[M]^-$, found 564.0031.

## S20

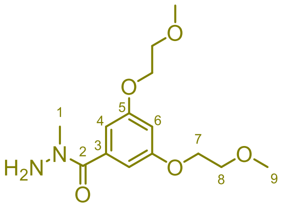


To a suspension of **S19** (510 mg, 0.90 mmol) in EtOH (10 mL), hydrazine monohydrate (65 wt%, 90 µL, 1.20 mmol, 1.3 equiv.) was added and the mixture was heated at 70 °C for 24 hours. The reaction was cooled to room temperature and the solvent was removed under reduced pressure. The crude residue was redissolved in $CHCl_3$ (80 mL), filtrated through a plug of Celite™ and the cake was washed with additional $CHCl_3$ (3 × 10 mL). The organic washes were combined and the solvent was removed under reduced pressure and the crude residue was purified by flash column chromatography ($SiO_2$, 5% MeOH in $CHCl_3$) to afford **S20** (230 mg, 0.77 mmol, 86%) as a light yellow oil.

**$^1$H NMR** (600 MHz, 298 K, Chloroform-*d*) δ 6.62 – 6.55 (m, 3H, $H_{4,6}$), 4.61 (s, 2H, $H_{NH2}$), 4.12 – 4.08 (m, 4H, $H_7$), 3.76 – 3.71 (m, 4H, $H_8$), 3.44 (s, 6H, $H_9$), 3.18 (s, 3H, $H_1$).

**$^{13}$C NMR** (151 MHz, 298 K, Chloroform-*d*) δ 169.7 ($C_2$), 160.0 ($C_5$), 137.0 ($C_3$), 106.2 ($C_4$), 101.4 ($C_6$), 71.0 ($C_8$), 67.6 ($C_7$), 59.4 ($C_9$), 40.6 ($C_1$).

**HRMS** (ASAP$^+$): Calculated for $C_{14}H_{23}O_5N_2$: 299.1601 $[M+H]^+$, found 299.1600.

**3**

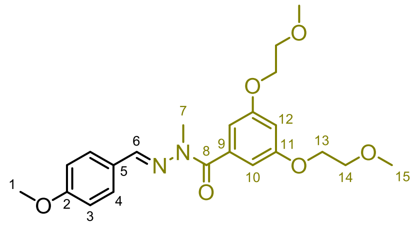


To a solution of **S20** (60 mg, 0.20 mmol) in $CH_2Cl_2$ (2.0 mL) at room temperature, 4-methoxybenzaldehyde (210 μL, 1.73 mmol, 8.5 equiv.) and $CF_3CO_2H$ (15.6 μL, 0.20 mmol, 1.0 equiv.) were added and the reaction was stirred for 10 minutes. $Et_3N$ (36 μL, 0.26 mmol, 1.3 equiv.) and water (5 mL) were added, and the product was extracted with $CH_2Cl_2$ (3 × 10 mL). The combined organic layers were washed with brine, dried over $MgSO_4$ and the solvent was removed under reduced pressure. The crude residue was purified by flash column chromatography ($SiO_2$, 1.5% MeOH in $CH_2Cl_2$) to afford **3** (68 mg, 0.16 mmol, 80%) as a colorless solid.

**m.p.** 105 – 107 °C.

**$^1$H NMR** (600 MHz, 298 K, Chloroform-*d*) δ 7.71 (s, 1H, $H_6$), 7.45 (d, *J* = 8.7 Hz, 2H, $H_4$), 6.88 – 6.84 (m, 4H, $H_{3,10}$), 6.66 (t, *J* = 2.3 Hz, 1H, $H_{12}$), 4.13 – 4.09 (m, 4H, $H_{13}$), 3.81 (s, 3H, $H_1$), 3.75 – 3.70 (m, 4H, $H_{14}$), 3.53 (s, 3H, $H_7$), 3.43 (s, 6H, $H_{15}$).

**$^{13}$C NMR** (151 MHz, 298 K, Chloroform-*d*) δ 170.8 ($C_6$), 161.0 ($C_2$), 159.1 ($C_{11}$), 139.2 ($C_6$), 137.4 ($C_9$), 128.8 ($C_4$), 127.6 ($C_5$), 114.3 ($C_3$), 108.6 ($C_{10}$), 104.4 ($C_{12}$), 71.0 ($C_{14}$), 67.6 ($C_{13}$), 59.3 ($C_{15}$), 55.5 ($C_1$), 28.9 ($C_7$).

**HRMS** (ASAP$^+$): Calculated for $C_{22}H_{29}O_6N_2$: 417.2020 $[M+H]^+$, found 417.2022.

**1**·H⁺

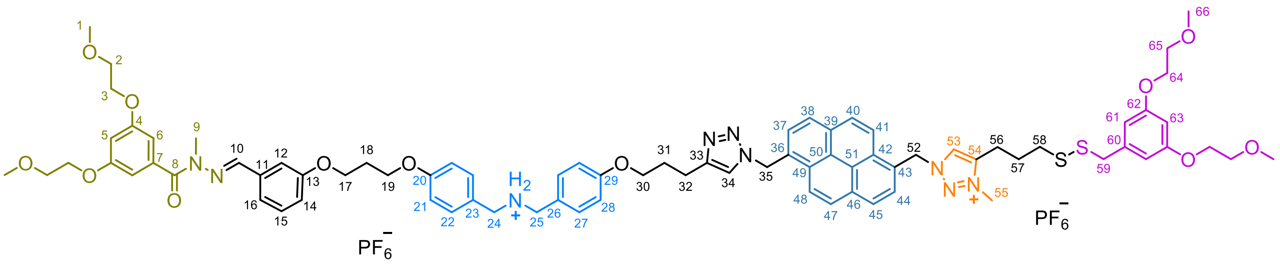

To a solution of **S16**·$H^+$ (5.0 mg, 3.4 μmol) in $CD_3CN$ (450 μL) at room temperature, **S20** (3.0 mg, 10.2 μmol, 3.0 equiv.) and $CF_3CO_2H$ (0.52 μL, 6.8 μmol, 2.0 equiv.) were added, and the reaction was thoroughly mixed. After the complete disappearance of the starting material`s aldehyde peak (by $^1$H NMR). The reaction mixture was basified with $Et_3N$ (2.0 μL, 14 μmol, 4.1 equiv.) and purified by size-exclusion chromatography (S-X1, $CH_2Cl_2$) and then anion-exchange chromatography (amberlyst A26 $PF_6^-$, MeCN) to give **1**·$H^+$ (4.5 mg, 2.6 μmol, 76%) as a yellow crystalline solid.

**$^1$H NMR** (600 MHz, 298 K, Acetonitrile-*d*$_3$) δ 8.51 (d, *J* = 9.2 Hz, 1H, $H_{48}$), 8.36 (d, *J* = 7.9 Hz, 1H, $H_{45}$), 8.33 (d, *J* = 7.9 Hz, 1H, $H_{38}$), 8.31 (d, *J* = 9.1 Hz, 1H, $H_{41}$), 8.27 (d, *J* = 9.1 Hz, 1H, $H_{40}$), 8.26 (d, *J* = 9.2 Hz, 1H, $H_{47}$), 8.18 (d, *J* = 7.9 Hz, 1H, $H_{44}$), 8.05 (s, 1H, $H_{53}$), 8.01 (d, *J* = 7.9 Hz, 1H, $H_{37}$), 7.82 (s, 1H, $H_{10}$), 7.56 (s, 1H, $H_{34}$), 7.35 (d, *J* = 8.7 Hz, 2H, $H_{22}$), 7.30 (d, *J* = 8.7 Hz, 2H, $H_{27}$), 7.26 (t, *J* = 7.9 Hz, 1H, $H_{15}$), 7.07 (dt, *J* = 7.6, 1.2 Hz, 1H, $H_{16}$), 7.04 (t, *J* = 1.9 Hz, 1H, $H_{12}$), 6.97 (d, *J* = 8.7 Hz, 2H, $H_{21}$), 6.89 (m, 3H, $H_{14,28}$), 6.75 (d, *J* = 2.3 Hz, 2H, $H_6$), 6.49 (t, *J* = 2.3 Hz, 1H, $H_5$), 6.41 (s, 2H, $H_{52}$), 6.37 (d, *J* = 2.3 Hz, 2H, $H_{61}$), 6.28 (t, *J* = 2.3 Hz, 1H, $H_{63}$), 6.26 (s, 2H, $H_{35}$), 4.15 (t, *J* = 6.2 Hz, 2H, $H_{17}$), 4.11 (s, 2H, $H_{24}$), 4.10 (s, 2H, $H_{25}$), 4.06 (s, 3H, $H_{55}$), 4.05 – 4.02 (m, 6H, $H_{3,19}$), 4.00 – 3.96 (m, 6H, $H_{30,64}$), 3.66 (s, 2H, $H_{59}$), 3.64 – 3.61 (m, 4H, $H_2$), 3.61 – 3.58 (m, 4H, $H_{65}$), 3.45 (s, 3H, $H_9$), 3.30 (s, 6H, $H_1$), 3.30 (s, 6H, $H_{66}$), 2.77 (t, *J* = 7.4 Hz, 2H, $H_{32}$), 2.72 (t, *J* = 7.6 Hz, 2H, $H_{56}$), 2.44 (t, *J* = 7.1 Hz, 2H, $H_{58}$), 2.18 (p, *J* = 6.2 Hz, 2H, $H_{18}$), 2.02 (p, *J* = 6.6 Hz, 2H, $H_{31}$), 1.83 (p, *J* = 7.4 Hz, 2H, $H_{57}$).

**$^{13}$C NMR** (151 MHz, 298 K, Acetonitrile-*d*$_3$) δ 171.1 ($C_8$), 160.9 ($C_{62}$), 160.84 ($C_{13}$), 160.81 ($C_{29}$), 160.1 ($C_{4,20}$), 148.2 ($C_{33}$), 145.1 ($C_{54}$), 141.0 ($C_{60}$), 140.2 ($C_{10}$), 139.2 ($C_7$), 137.6 ($C_{11}$), 133.1 ($C_{46}$), 132.7 ($C_{22}$), 132.7 ($C_{27}$), 131.9 ($C_{39}$), 131.2 ($C_{49}$), 130.8 ($C_{15}$), 130.7 ($C_{42}$), 130.2 ($C_{44}$), 130.0 ($C_{40}$), 129.9 ($C_{50}$), 129.2 ($C_{47}$), 129.0 ($C_{37}$), 128.8 ($C_{53}$), 127.1 ($C_{38}$), 126.7 ($C_{45}$), 125.9 ($C_{51}$), 125.6 ($C_{43}$), 125.3 ($C_{36}$), 124.8 ($C_{48}$), 123.5 ($C_{41}$), 123.3 ($C_{23}$), 123.2 ($C_{26}$), 122.7 ($C_{34}$), 121.4 ($C_{16}$), 117.4 ($C_{14}$), 115.8 ($C_{21}$), 115.7 ($C_{28}$), 111.5 ($C_{12}$), 108.8 ($C_{61}$), 108.7 ($C_6$), 103.7 ($C_5$), 100.7 ($C_{63}$), 71.5 ($C_{65}$), 71.5 ($C_2$), 68.5 ($C_3$), 68.2 ($C_{64}$), 68.0 ($C_{30}$), 65.5 ($C_{17}$), 65.1 ($C_{19}$), 59.0 ($C_1$), 58.9 ($C_{66}$), 55.9 ($C_{52}$), 52.3 ($C_{35}$), 51.6 ($C_{24,25}$), 43.3 ($C_{59}$), 38.4 ($C_{55}$), 36.9 ($C_{58}$), 29.7 ($C_{18}$), 29.6 ($C_{31}$), 29.1 ($C_9$), 26.5 ($C_{57}$), 22.6 ($C_{32}$), 22.4 ($C_{56}$).

**HRMS** (ESI$^+$): Calculated for $C_{80}H_{92}O_{12}N_9S_2^+$: 1434.6301 $[M]^+$, found 1434.6281.

# 4 Operation of 1

## 4.1 Stepwise Formation of *dba*-2·H$^+$

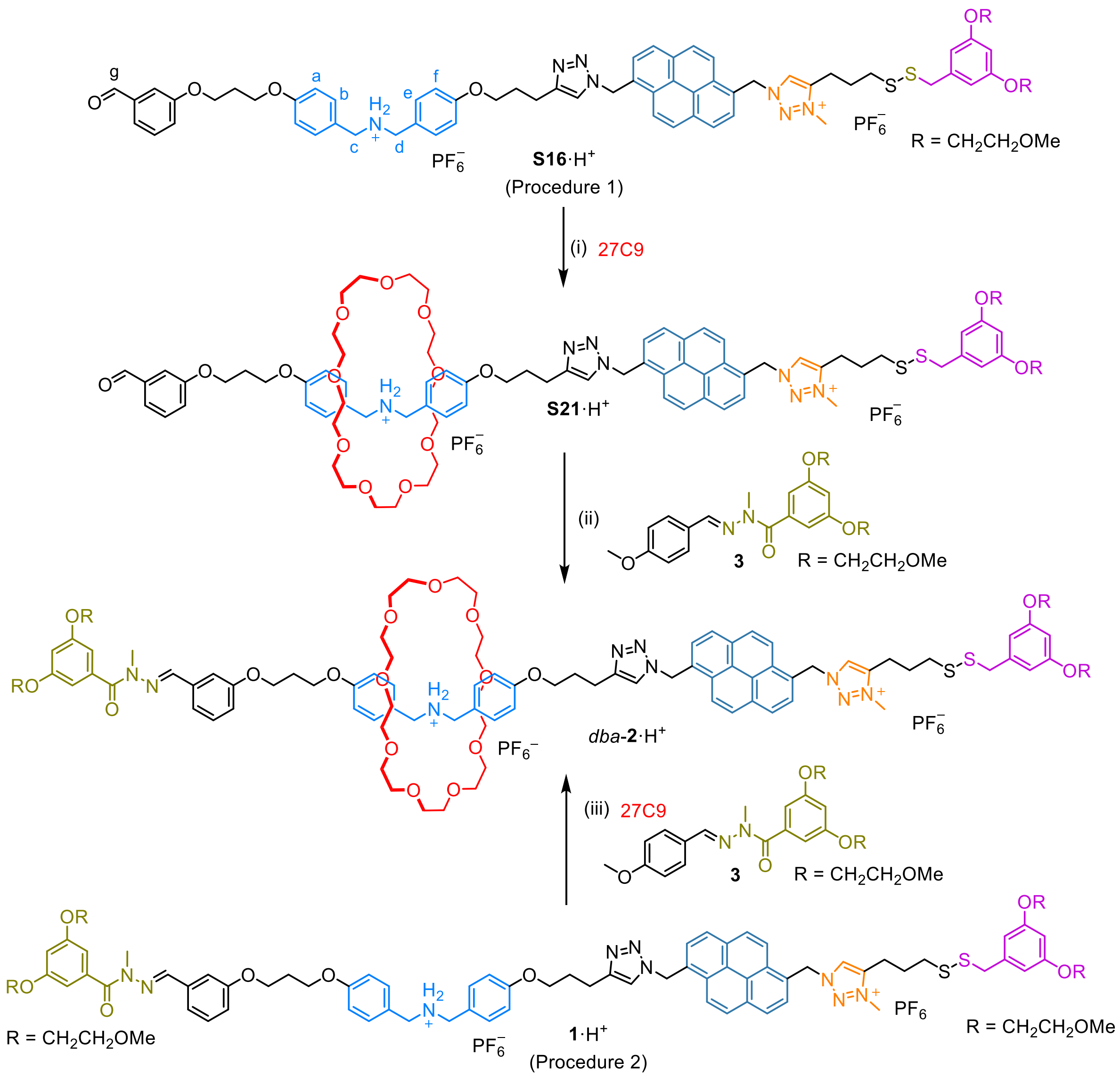


**Scheme S6** – Synthesis of rotaxane *dba*-**2**·H$^+$. Reagents and conditions: (Procedure 1) – (i) **S16**·H$^+$ (19 mM), $CF_3CO_2H$ (5.0 equiv.), 27C9 (5.0 equiv.), $CD_3CN$ (500 µL), rt, 10 mins, *then* (ii) **3** (3.0 equiv.), rt, 1 hour, *then* separation by size-exclusion chromatography and anion-exchange (amberlyst A26/ $PF_6^-$, MeCN), 26%; (Procedure 2) – (iii) **1**·H$^+$ (12 mM), $PhNH_2$ (0.5 equiv.) $CF_3CO_2H$ (5.0 equiv.), 27C9 (5.0 equiv.), **3** (3.0 equiv.), $CD_3CN$ (500 µL), rt, 5 days, *then* separation by size-exclusion chromatography and anion-exchange (amberlyst A26/ $PF_6^-$, MeCN), 21%.

*dba*-**2**·H⁺

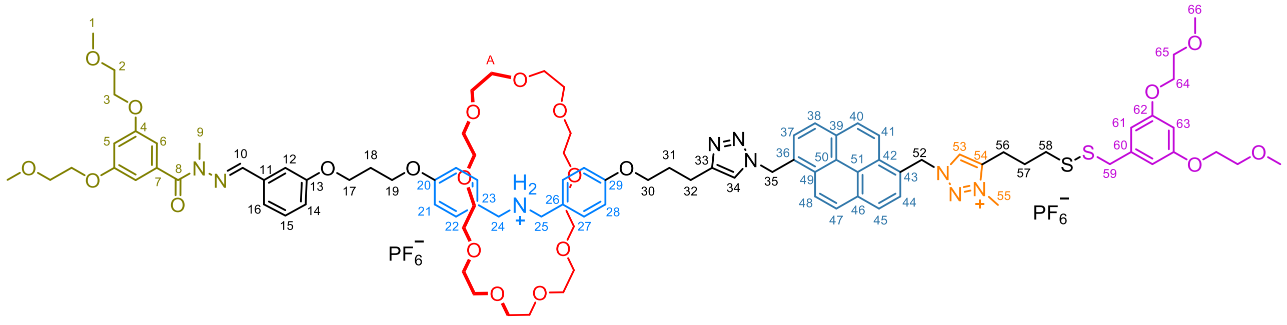

*dba*-**2**·H⁺

Procedure 1. To a solution of **S16**·H⁺ (13.7 mg, 9.5 µmol), $CF_3CO_2H$ (3.7 µL, 48 µmol, 5.0 equiv.) and 27C9 (19.0 mg, 48 µmol, 5.0 equiv.) in $CD_3CN$ (500 µL) at room temperature, hydrazone **3** (11.8 mg, 28 µmol, 3.0 equiv.) was added and the solution was allowed to equilibrate until the aldehyde peak corresponding to **S16**·H⁺ disappeared completely (the process was followed by $^1$H NMR, see Figure S1). $Et_3N$ (6.7 µL, 48 µmol, 5.0 equiv.) was added and the mixture was purified by size-exclusion chromatography (S-X1, $CH_2Cl_2$). The product was passed through an anion-exchange column (amberlyst A-26 $PF_6^-$, MeCN) to give *dba*-**2**·H⁺ (5.3 mg, 2.5 µmol, 26%) as a yellow crystalline solid.

Procedure 2. To a solution of **1**·H⁺ (10.0 mg, 5.8 µmol) in $CD_3CN$ (500 µL, 12 mM) at room temperature, $CF_3CO_2H$ (2.2 µL, 29 µmol, 5.0 equiv.), $PhNH_2$ (0.1 M in $CD_3CN$, 29 µL, 2.9 µmmol, 0.5 equiv.), hydrazone **3** (7.2 mg, 17.4 µmol, 3.0 equiv.) and 27C9 (11.5 mg, 29 µmol, 5.0 equiv.) were added and the mixture was allowed to equilibrate at room temperature for 5 days. $Et_3N$ (4.1 µL, 29 µmol, 5.0 equiv.) was then added and the mixture was purified by size-exclusion chromatography (S-X1, $CH_2Cl_2$). The product was passed through an anion-exchange column (amberlyst A-26 $PF_6^-$, MeCN) to give *dba*-**2**·H⁺ (2.6 mg, 1.2 µmol, 21%) as a light-yellow film. Spectral data was identical to that obtained *via* procedure 1.

**$^1$H NMR** (600 MHz, 298 K, Acetonitrile-*d*$_3$) δ 8.53 (d, $J$ = 9.2 Hz, 1H, $H_{48}$), 8.37 (d, $J$ = 7.9 Hz, 1H, $H_{45}$), 8.34 (d, $J$ = 7.9 Hz, 1H, $H_{38}$), 8.32 (d, $J$ = 9.2 Hz, 1H, $H_{41}$), 8.30 – 8.26 (m, 2H, $H_{40,47}$), 8.20 (d, $J$ = 7.9 Hz, 1H, $H_{44}$), 8.06 (s, 1H, $H_{53}$), 8.01 (d, $J$ = 7.9 Hz, 1H, $H_{37}$), 7.81 (s, 1H, $H_{10}$), 7.57 (s, 1H, $H_{34}$), 7.43 (d, $J$ = 8.9 Hz, 2H, $H_{22}$), 7.41 (d, $J$ = 8.7 Hz, 2H, $H_{27}$), 7.25 (t, $J$ = 7.8 Hz, 1H, $H_{15}$), 7.06 (d, $J$ = 7.8 Hz, 1H, $H_{16}$), 7.05 (s, 1H, $H_{12}$), 6.96 (d, $J$ = 8.9 Hz, 2H, $H_{21}$), 6.91 (d, $J$ = 8.6 Hz, 2H, $H_{28}$), 6.89 (dd, $J$ = 7.3, 2.2 Hz, 1H, $H_{14}$), 6.75 (d, $J$ = 2.3 Hz, 2H, $H_6$), 6.55 (t, $J$ = 2.3 Hz, 1H, $H_5$), 6.42 (s, 2H, $H_{52}$), 6.38 (d, $J$ = 2.3 Hz, 2H, $H_{61}$), 6.29 (t, $J$ = 2.3 Hz, 1H, $H_{63}$), 6.26 (s, 2H, $H_{35}$), 4.34 – 4.26 (m, 4H, $H_{24,25}$), 4.17 (t, $J$ = 6.3 Hz, 2H, $H_{17}$), 4.09 – 4.05 (m, 4H, $H_3$), 4.06 (s, 3H, $H_{55}$), 4.04 – 4.00 (m, 4H, $H_{19,30}$), 3.99 – 3.97 (m, 4H, $H_{64}$), 3.66 (s, 2H, $H_{59}$), 3.66 – 3.62 (m, 4H, $H_2$), 3.62 – 3.58 (m, 4H, $H_{65}$), 3.45 (s, 3H, $H_9$), 3.39 (s, 36H, $H_A$), 3.31 (s, 6H, $H_1$), 3.30 (s, 6H, $H_{66}$), 2.76 (t, $J$ = 7.7 Hz, 2H, $H_{32}$), 2.72 (t, $J$ = 7.6 Hz, 2H, $H_{56}$), 2.44 (t, $J$ = 7.1 Hz, 2H, $H_{58}$), 2.18 (p, $J$ = 6.0 Hz, 2H, $H_{18}$), 2.02 (p, $J$ = 6.6 Hz, 2H, $H_{31}$), 1.83 (p, $J$ = 7.3 Hz, 2H, $H_{57}$).

**$^{13}$C NMR** (151 MHz, 298 K, Acetonitrile-*d*$_3$) δ 171.1 ($C_8$), 160.9 ($C_{62}$), 160.6 ($C_{20}$), 160.5 ($C_{29}$), 160.1 ($C_4$), 160.1 ($C_{13}$), 148.2 ($C_{33}$), 145.1 ($C_{54}$), 141.0 ($C_{60}$), 140.2 ($C_{10}$), 139.2 ($C_7$), 137.7 ($C_{11}$), 133.1

($C_{46}$), 133.0 ($C_{22,27}$), 131.9 ($C_{39}$), 131.2 ($C_{49}$), 130.8 ($C_{15}$), 130.7 ($C_{42}$), 130.3 ($C_{44}$), 130.0 ($C_{40}$), 129.9 ($C_{50}$), 129.2 ($C_{47}$), 129.0 ($C_{37}$), 128.9 ($C_{53}$), 127.1 ($C_{38}$), 126.7 ($C_{45}$), 126.0 ($C_{51}$), 125.6 ($C_{43}$), 125.3 ($C_{36}$), 125.0 ($C_{23}$), 124.9 ($C_{26}$), 124.8 ($C_{48}$), 123.5 ($C_{41}$), 122.7 ($C_{34}$), 121.3 ($C_{16}$), 117.4 ($C_{14}$), 115.4 ($C_{21}$), 115.3 ($C_{28}$), 111.5 ($C_{12}$), 108.8 ($C_{61}$), 108.6 ($C_{6}$), 103.7 ($C_{5}$), 100.7 ($C_{63}$), 71.5 ($C_{2,65}$), 71.1 ($C_{A}$), 68.5 ($C_{3}$), 68.2 ($C_{64}$), 68.0 ($C_{30}$), 65.5 ($C_{17}$), 65.0 ($C_{19}$), 59.0 ($C_{66}$), 59.0 ($C_{1}$), 55.9 ($C_{52}$), 52.5 ($C_{24,25}$), 52.3 ($C_{35}$), 43.4 ($C_{59}$), 38.4 ($C_{55}$), 36.9 ($C_{58}$), 29.8 ($C_{18}$), 29.5 ($C_{31}$), 29.1 ($C_{9}$), 26.5 ($C_{57}$), 22.6 ($C_{32}$), 22.4 ($C_{56}$).

**HRMS** (ESI$^+$): Calculated for $C_{98}H_{129}O_{21}N_9S_2^{2+}$: 915.9367 $[M]^{2+}$, found 915.9361.

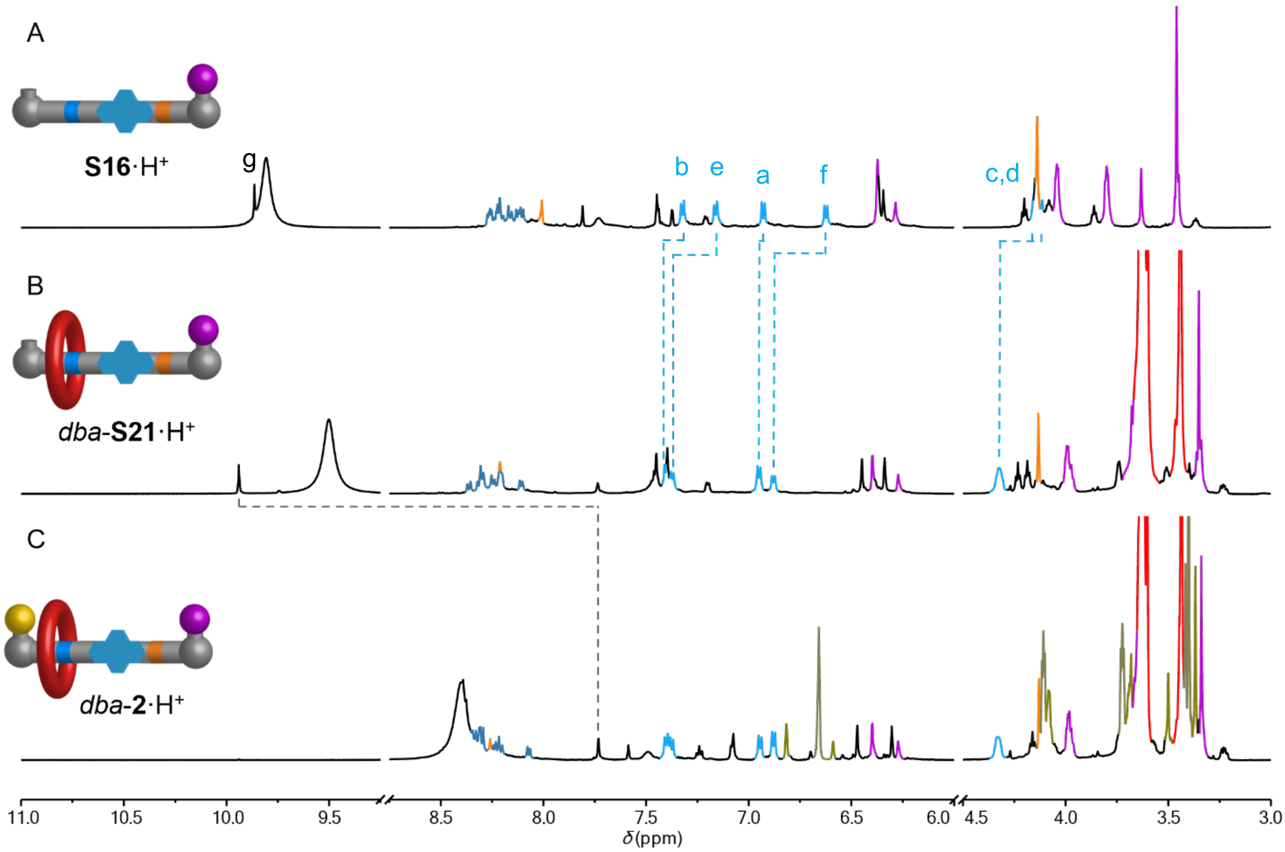


**Figure S1** – Partial $^1$H NMR (600 MHz, 298 K, $CD_3CN$) of the stepwise rotaxane formation from **S16**·H$^+$: (A) After addition of $CF_3CO_2H$ (5.0 equiv.); (B) After addition of 27C9 (5.0 equiv.); (C) After addition of **3** (3.0 equiv.). The lettering relates protons in the chemical structures shown in Scheme S6 to the corresponding signals in the $^1$H NMR spectra.

## 4.2 Switching and Dethreading of *dba*-2·H⁺

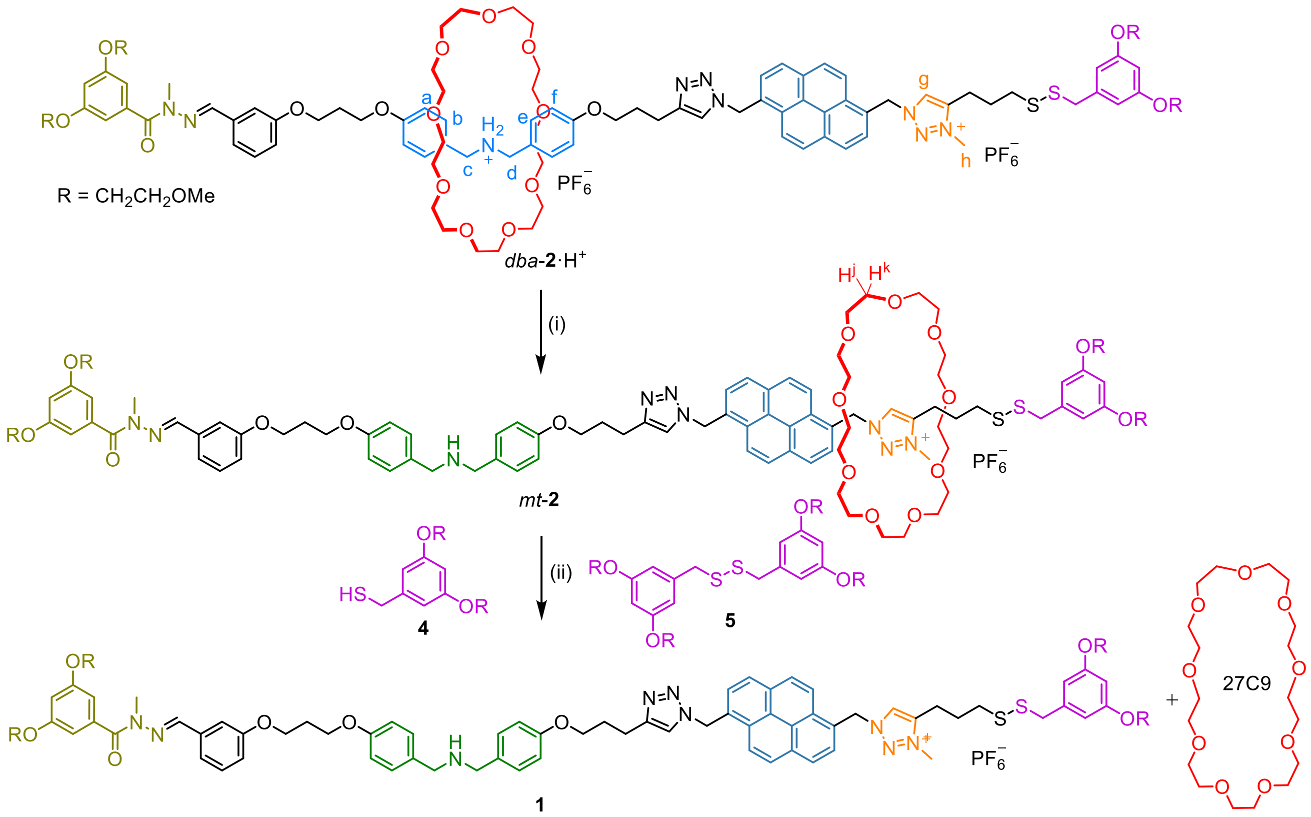


**Scheme S7** – Switching of *dba*-**2**·$H^+$ to *mt*-**2**, followed by dethreading under disulfide exchange conditions to give **1** and 27C9. Reagents and conditions: (i) *dba*-**2**·$H^+$ (1.6 mM), $Et_3N$ (50 eq), $CD_3CN$ (500 µL), *then* (ii) thiol **4** (2.0 equiv.) and disulfide **5** (20 equiv.).

*mt*-**2**

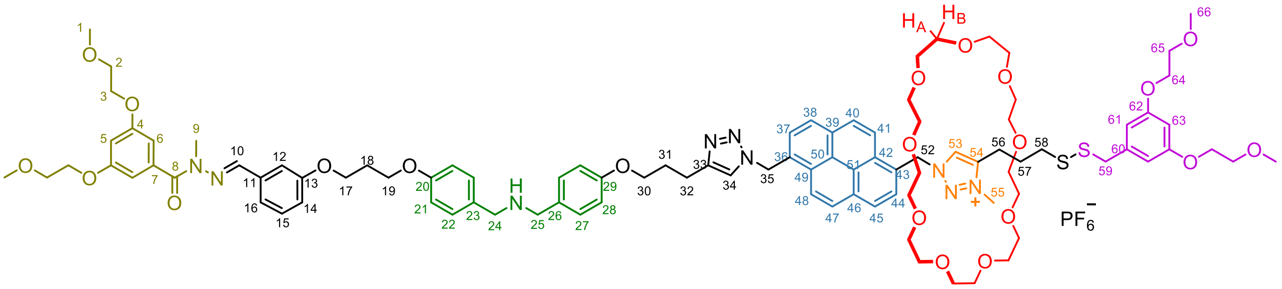

To a solution of *dba*-**2**·$H^+$ (1.7 mg, 0.8 μmol) in $CD_3CN$ (500 μL), $Et_3N$ (5.6 μL, 40 μmol, 50 equiv.) was added and the $^1H$ NMR was recorded (see Figure S2). Thiol **4** (0.4 mg, 1.6 μmol, 2.0 equiv.) and disulfide **5** (8.7 mg, 16 μmol, 20 equiv.) were then added to initiate dethreading of 27C9 and formation of **1** (see Figure S3).

**$^1$H NMR** (600 MHz, 298 K, Acetonitrile-*d*$_3$) δ 8.76 (s, 1H, $H_{53}$), 8.50 (d, *J* = 9.2 Hz, 1H, $H_{48}$), 8.47 (d, *J* = 9.2 Hz, 1H, $H_{41}$), 8.34 (d, *J* = 7.9 Hz, 1H, $H_{45}$), 8.31 (d, *J* = 7.9 Hz, 1H, $H_{38}$), 8.28 – 8.22 (m, 3H, $H_{40,44,47}$), 8.02 (d, *J* = 7.9 Hz, 1H, $H_{37}$), 7.81 (s, 1H, $H_{10}$), 7.49 (s, 1H, $H_{34}$), 7.25 (t, *J* = 7.9 Hz, 1H, $H_{15}$), 7.22 (d, *J* = 8.4 Hz, 2H, $H_{22}$), 7.09 (d, *J* = 8.6 Hz, 2H, $H_{27}$), 7.06 (d, *J* = 7.7 Hz, 1H, $H_{16}$), 7.05 (d, *J* = 2.0 Hz, 1H, $H_{12}$), 6.89 (dd, *J* = 8.2, 2.7 Hz, 1H, $H_{14}$), 6.87 (d, *J* = 8.4 Hz, 2H, $H_{21}$), 6.75 (d, *J* = 2.4 Hz, 2H, $H_6$), 6.67 (d, *J* = 8.6 Hz, 2H, $H_{28}$), 6.53 (t, *J* = 2.4 Hz, 1H, $H_5$), 6.49 (d, *J* = 2.2 Hz, 2H, $H_{61}$), 6.41 (s, 2H, $H_{52}$), 6.37 (t, *J* = 2.2 Hz, 1H, $H_{63}$), 6.26 (s, 2H, $H_{35}$), 4.24 (s, 3H, $H_{55}$), 4.11 (t, *J* = 6.2 Hz, 2H, $H_{17}$), 4.05 – 4.01 (m, 10H, $H_{3,19,64}$), 3.87 (t, *J* = 6.3 Hz, 2H, $H_{30}$), 3.83 (s, 2H, $H_{59}$), 3.65 – 3.62 (m, 8H, $H_{2,65}$), 3.61 (s, 2H, $H_{24}$), 3.58 (s, 2H, $H_{25}$), 3.45 (s, 3H, $H_9$), 3.32 (s, 6H, $H_1$), 3.30 (s, 6H, $H_{66}$), 3.31 – 3.26 (m, 18H, $H_{A/B}$), 3.15 (td, *J* = 8.1, 7.2, 3.2 Hz, 18H, $H_{A/B}$), 2.88 (t, *J* = 7.8 Hz, 2H, $H_{56}$), 2.77 (t, *J* = 7.4 Hz, 2H, $H_{32}$), 2.63 (t, *J* = 7.3 Hz, 2H, $H_{58}$), 2.16 (p, *J* = 6.3 Hz, 2H, $H_{18}$), 2.04 (p, *J* = 7.5 Hz, 2H, $H_{57}$), 1.98 (p, *J* = 7.0 Hz, 2H, $H_{31}$).

**$^{13}$C NMR** (151 MHz, 298 K, Acetonitrile-*d*$_3$) δ 171.1 ($C_8$), 161.0 ($C_{62}$), 160.3 ($C_{13}$), 160.1 ($C_4$), 158.71 ($C_{20}$), 158.65 ($C_{29}$), 148.2 ($C_{33}$), 145.2 ($C_{54}$), 141.2 ($C_{60}$), 140.2 ($C_{10}$), 139.2 ($C_7$), 137.6 ($C_{11}$), 134.1 ($C_{23}$), 133.9 ($C_{26}$), 133.0 ($C_{46}$), 132.0 ($H_{39}$), 131.7 ($C_{53}$), 130.8 ($C_{15}$), 130.7 ($C_{42}$), 130.2 ($C_{22}$), 130.1 ($C_{27}$), 129.9 ($C_{49}$), 129.7 ($C_{44}$), 129.5 ($C_{40}$), 129.4 ($C_{50}$), 129.2 ($C_{47}$), 129.1 ($C_{37}$), 126.8 ($C_{38}$), 126.6 ($C_{45}$), 125.4 ($C_{51}$), 124.6 ($C_{41}$), 124.4 ($C_{48}$), 123.7 ($C_{43}$), 123.3 ($C_{36}$), 122.7 ($C_{34}$), 121.3 ($C_{16}$), 117.4 ($C_{14}$), 115.1 ($C_{21}$), 115.0 ($C_{28}$), 111.6 ($C_{12}$), 108.8 ($C_{61}$), 108.7 ($C_6$), 103.6 ($C_5$), 100.7 ($C_{63}$), 71.5 ($C_2$), 71.2 ($C_{A,B}$), 71.0 ($C_{65}$), 68.5 ($C_{64}$), 68.3 ($C_3$), 67.6 ($C_{30}$), 65.2 ($C_{17}$), 65.2 ($C_{19}$), 59.3 ($C_9$), 59.0 ($C_1$), 59.0 ($C_{66}$), 55.3 ($C_{52}$), 52.9 ($C_{24,25}$), 52.4 ($C_{35}$), 43.8 ($C_{59}$), 38.3 ($C_{55}$), 36.9 ($C_{58}$), 29.9 ($C_{18}$), 29.5 ($C_{31}$), 26.6 ($C_{57}$), 22.7 ($C_{32}$), 22.4 ($C_{56}$).

**HRMS** (ESI$^+$): Calculated for $C_{98}H_{128}O_{21}N_9S_2^+$: 1830.8661 $[M]^+$, found 1830.8638.

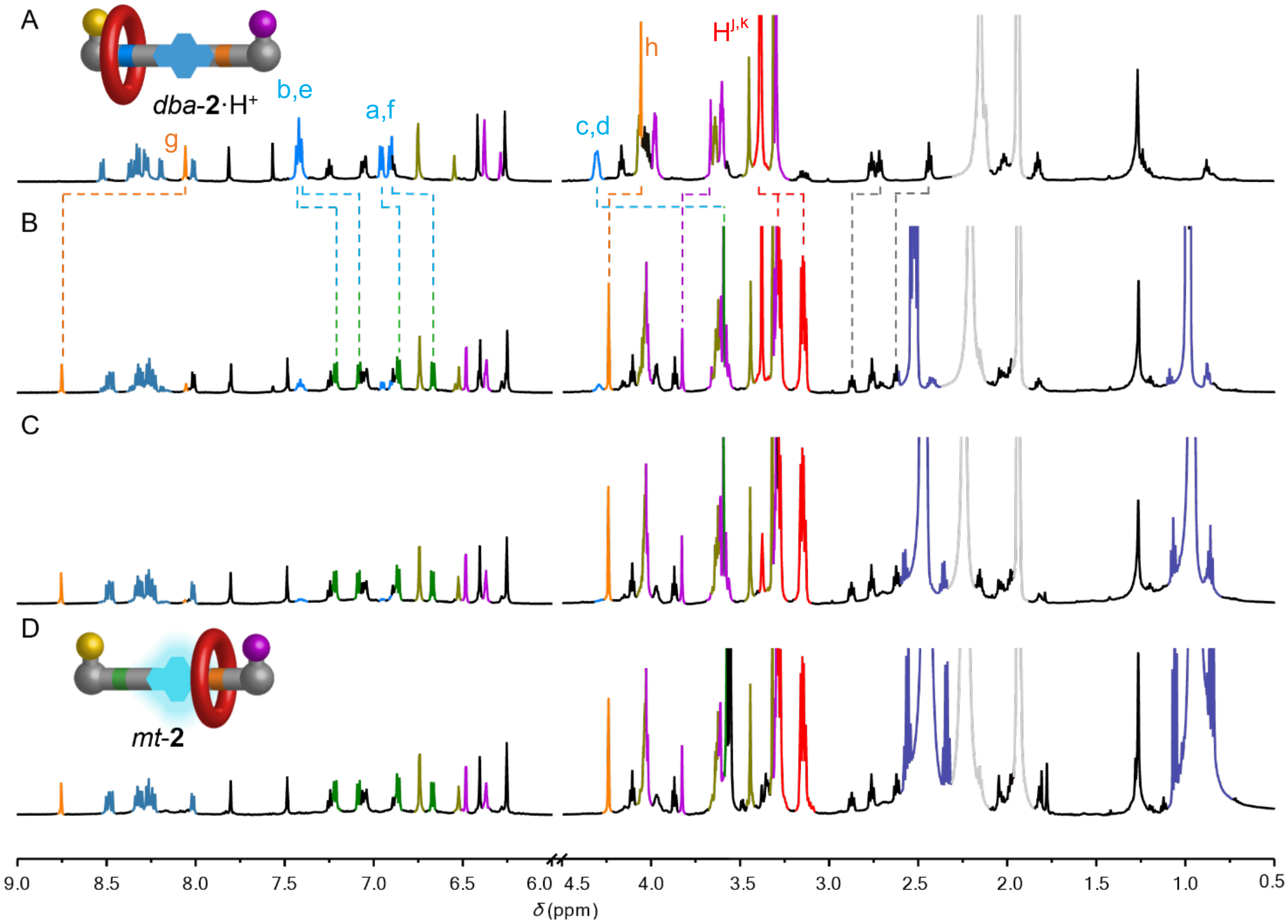


**Figure S2** – Partial $^1$H NMR (600 MHz, 298 K, $CD_3CN$) of the switching process with $Et_3N$: (A) Protonated rotaxane *dba*-**2**·$H^+$; (B) After addition of triethylamine (5.0 equiv.); (C) After addition of triethylamine (20 equiv.) and (D) After addition of triethylamine (50 equiv.). The lettering relates protons in the chemical structures shown in Scheme S7 to the corresponding signals in the $^1$H NMR spectra. Peaks shaded gray correspond to residual solvent, peaks shaded dark blue correspond to triethylamine.

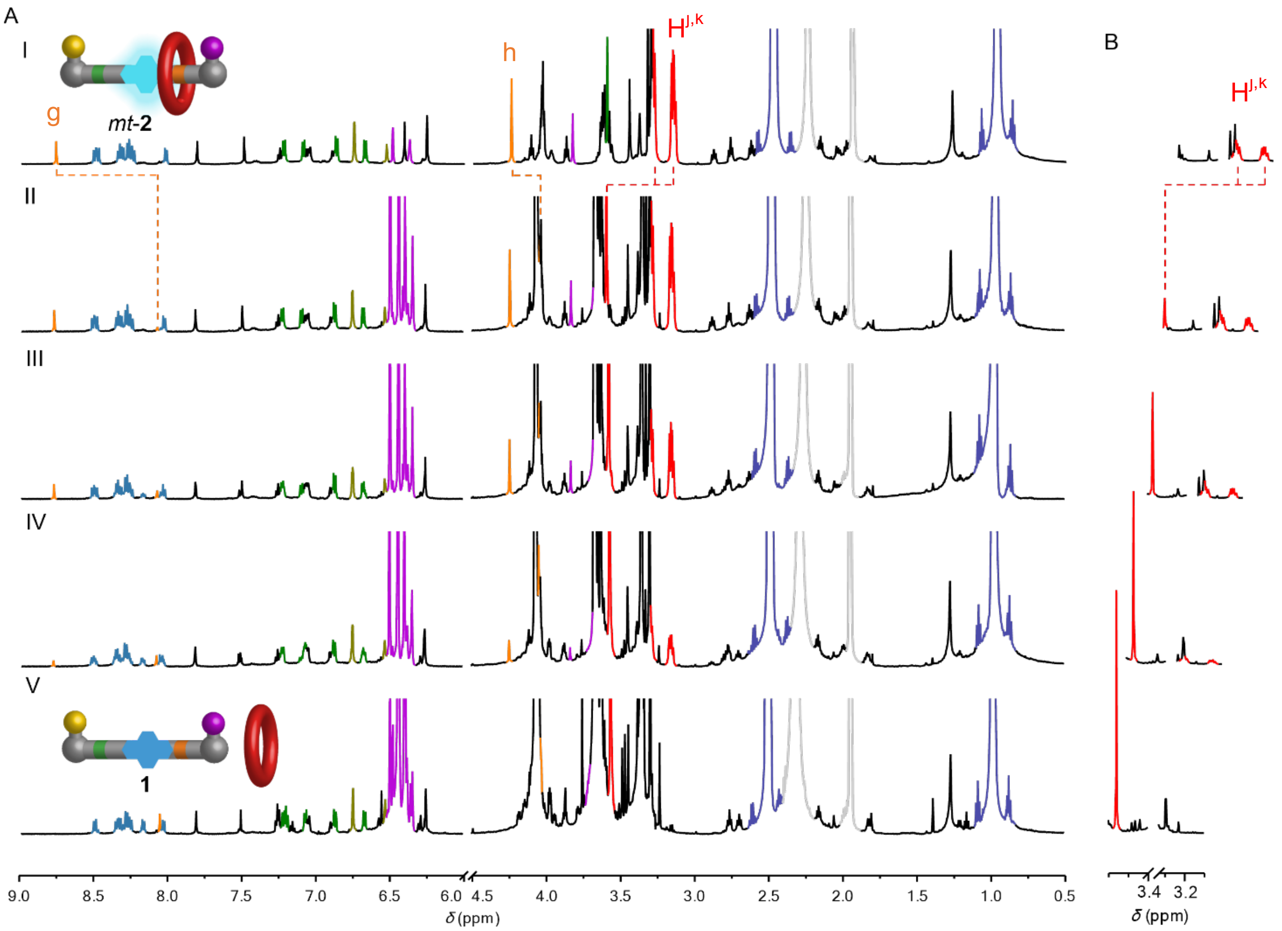


**Figure S3** – (A) Partial $^{1}$H NMR (600 MHz, 298 K, $CD_3CN$) of the stepwise dethreading of *mt*-**2**: (I) *mt*-**2** and triethylamine (50 equiv.); (II) 10 minutes after addition of thiol **4** (2.0 equiv.) and disulfide **5** (20 equiv.); (III) 1 hour after addition of thiol **4** and disulfide **5**; (IV) 3 hours after addition of thiol **4** and disulfide **5** and (V) 24 hours after addition of thiol **4** and disulfide **5**; (B) Partial $^{1}$H NMR (600 MHz, 298 K, $CD_3CN$) of the dethreading of *mt*-**2** focused on the region of the spectra containing the proton shifts of the 27C9 macrocycle over the same time periods. The lettering relates protons in the chemical structures shown in Scheme S7 to the corresponding signals in the $^{1}$H NMR spectra. Peaks shaded gray correspond to residual solvent, peaks shaded dark blue correspond to triethylamine.

### 4.3 Pulsed Fuel Operation of 1.

To a solution of **1**·H$^+$ (2.0 mg, 1.2 µmol) in $CD_3CN$ (600 µL), $Et_3N$ (8.4 µL, 60 µmol, 50 equiv.), $PhNH_2$ (0.1 M in $CD_3CN$, 6 µL, 0.6 µmmol, 0.5 equiv.), hydrazone **3** (1.5 mg, 3.6 µmol, 3.0 equiv.) and 27C9 (9.5 mg, 24 µmol, 20 equiv.), thiol **4** (0.6 mg, 2.4 µmol, 2.0 equiv.) and disulfide **5** (13.0 mg, 24 µmol, 20 equiv.) were added. To this, 10 M $CCl_3CO_2H$ in $CD_3CN$ (24 µL, 240 µmol, 200 equiv.) was added and the reaction was monitored by $^1$H NMR (Figure S4-6).

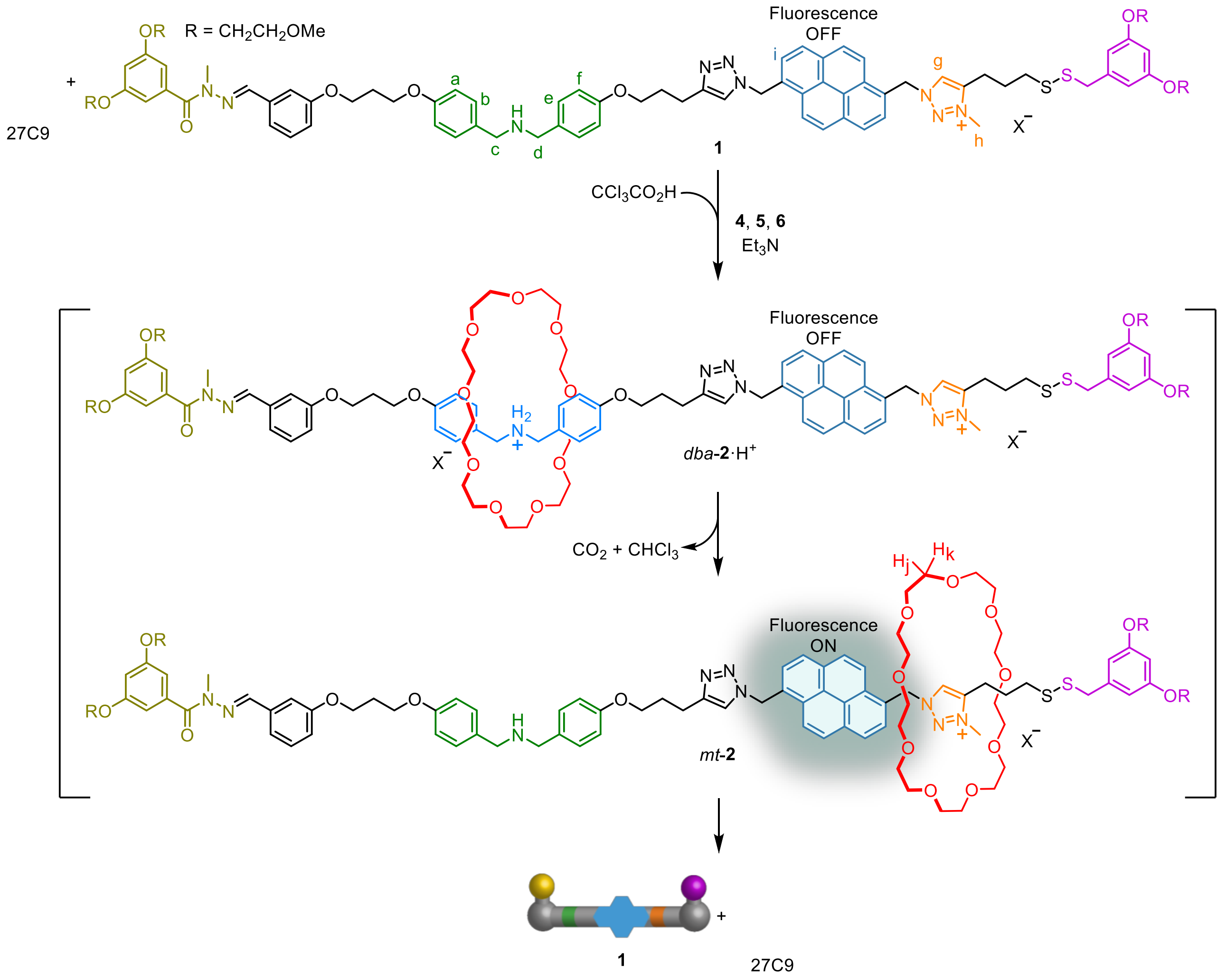


**Scheme S8** – Pulsed fuel operation of **1**. Reagents and conditions: **1**· H$^+$ (1.9 mM), $Et_3N$ (50 equiv.), $PhNH_2$ (0.5 equiv.), 27C9 (20 equiv.), hydrazone **3** (3.0 equiv.), thiol **4** (2.0 equiv.), disulfide **5** (20 equiv.) in $CD_3CN$, *then* 10 M $CCl_3CO_2H$ in $CD_3CN$ (200 equiv.).

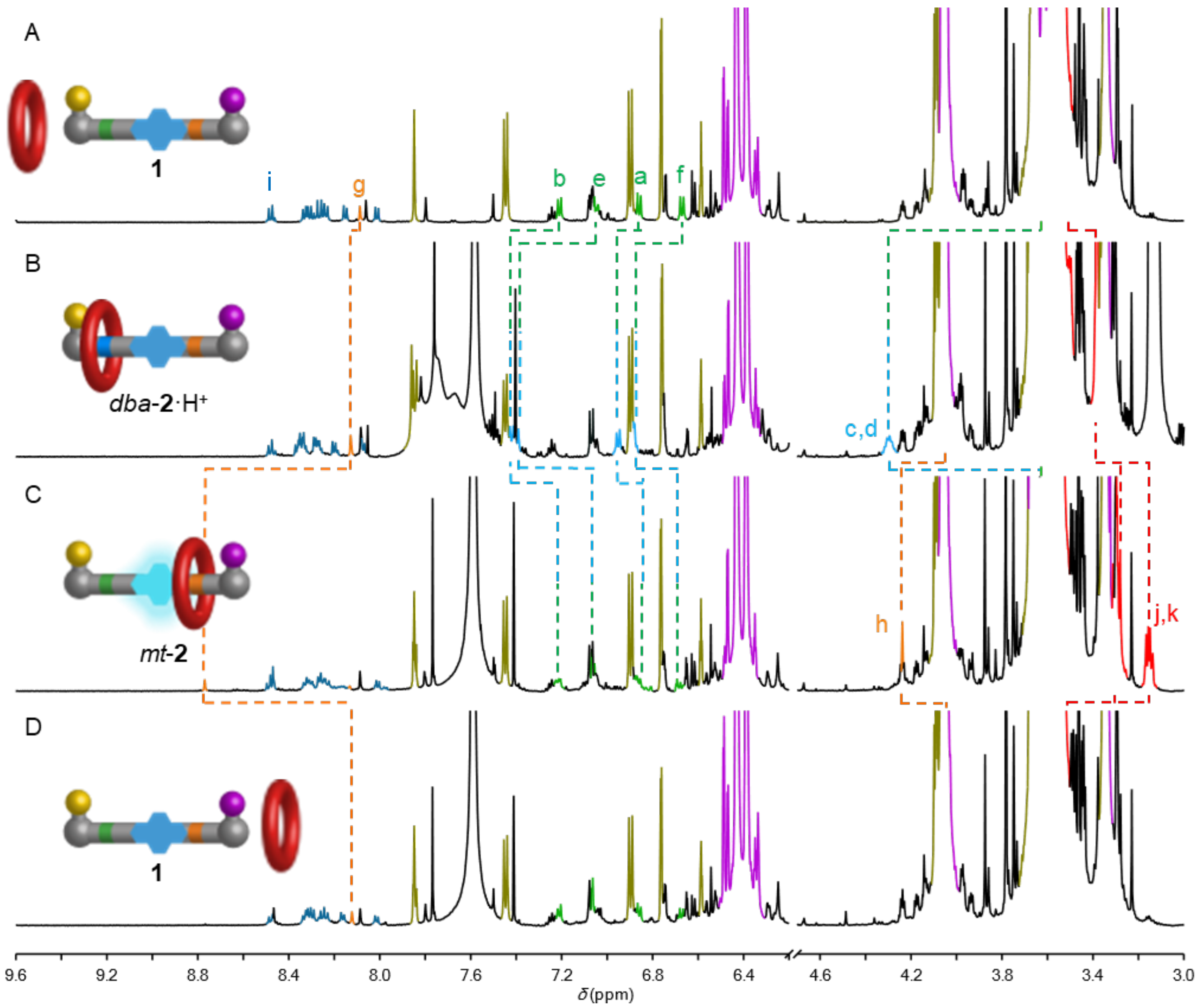


**Figure S4** – Partial $^{1}$H NMR (600 MHz, 298 K, $CD_3CN$) of the pulse-fuelled operation: (A) before addition of fuel; (B) Right before complete fuel consumption; (C) Immediately after fuel consumption; (D) After complete dethreading. The lettering relates protons in the chemical structures shown in Scheme S8 to the corresponding signals in the $^{1}$H NMR spectra.

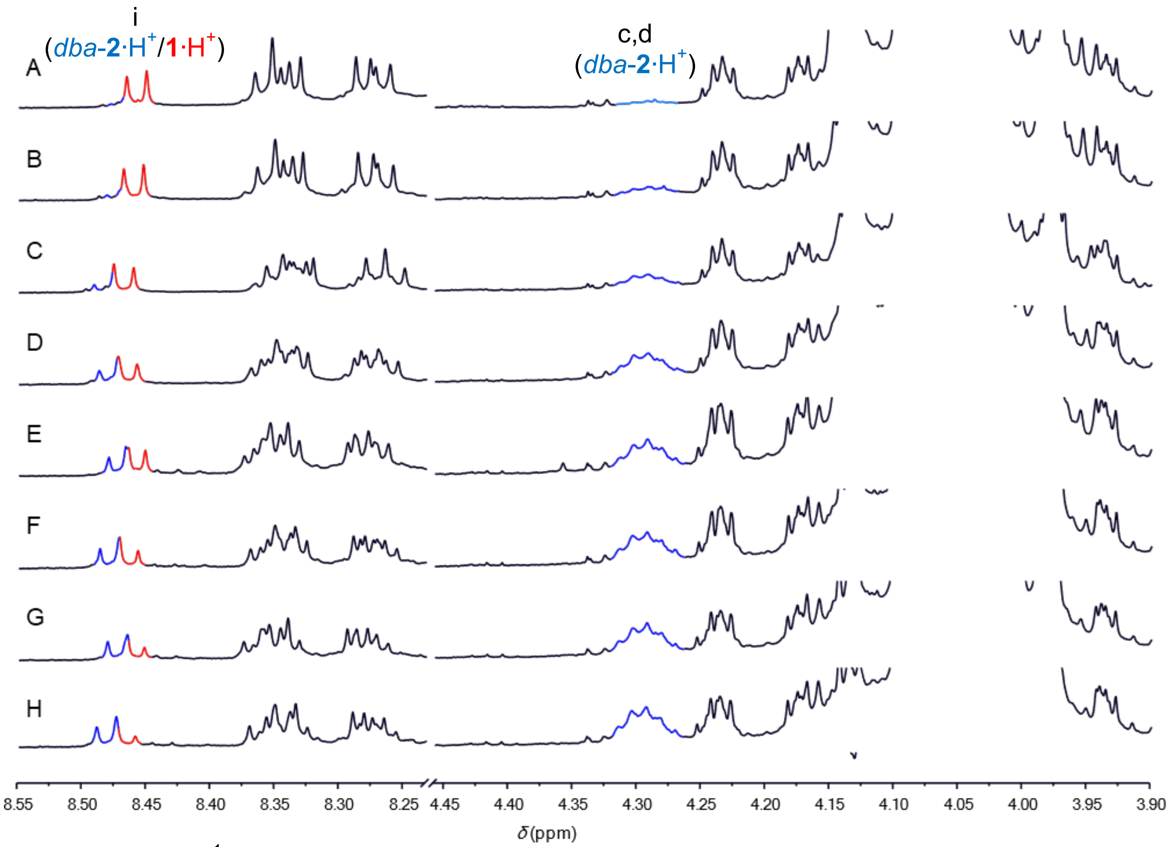


**Figure S5** – Partial $^{1}H$ NMR (600 MHz, 298 K, $CD_3CN$) of pulse-fuelled threading: (A) 5 minutes after the addition of $CCl_3CO_2H$; (B) After 7 hours; (C) After 1 day; (D) After 2 days; (E) After 3 days; (F) After 4 days; (G) After 5 days and (H) After 6 days. The lettering relates protons in the chemical structures shown in Scheme S8 to the corresponding signals in the $^{1}H$ NMR spectra.

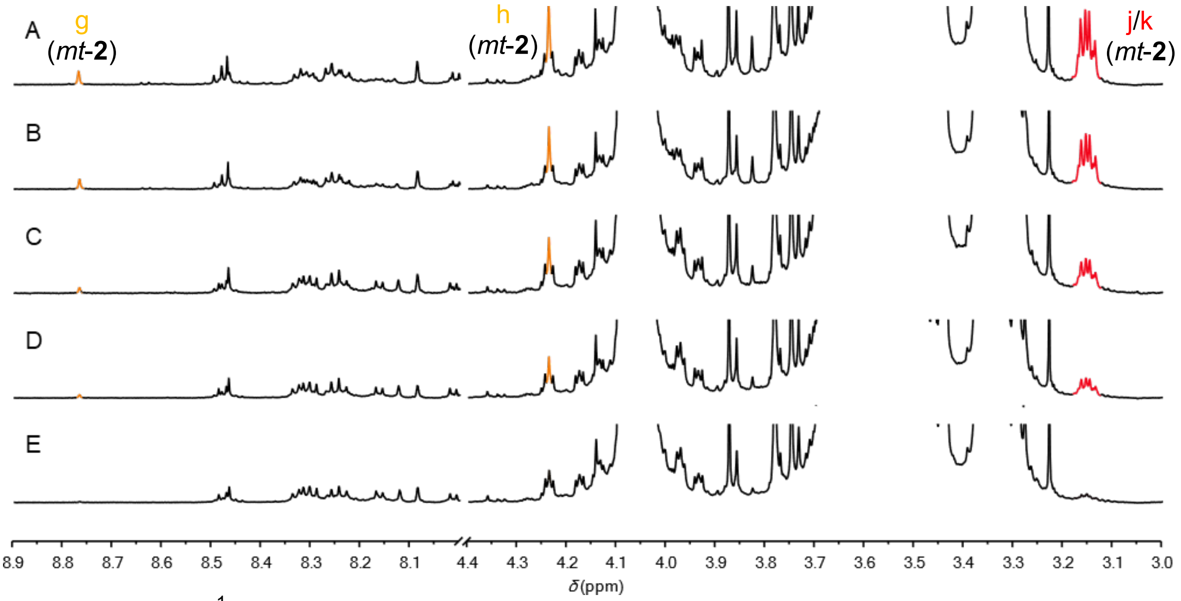


**Figure S6** – Partial $^{1}H$ NMR (600 MHz, 298 K, $CD_3CN$) of pulse-fuelled dethreading: (A) After 5 minutes; (B) After 1 hour; (C) After 4 hours; (D) After 8 hours and (E) After 12 hours. The lettering relates protons in the chemical structures shown in Scheme S8 to the corresponding signals in the $^{1}H$ NMR spectra.

# 5 Fluorescence Spectroscopy

## 5.1 UV-Vis and Fluorescence Emission Spectra of 1

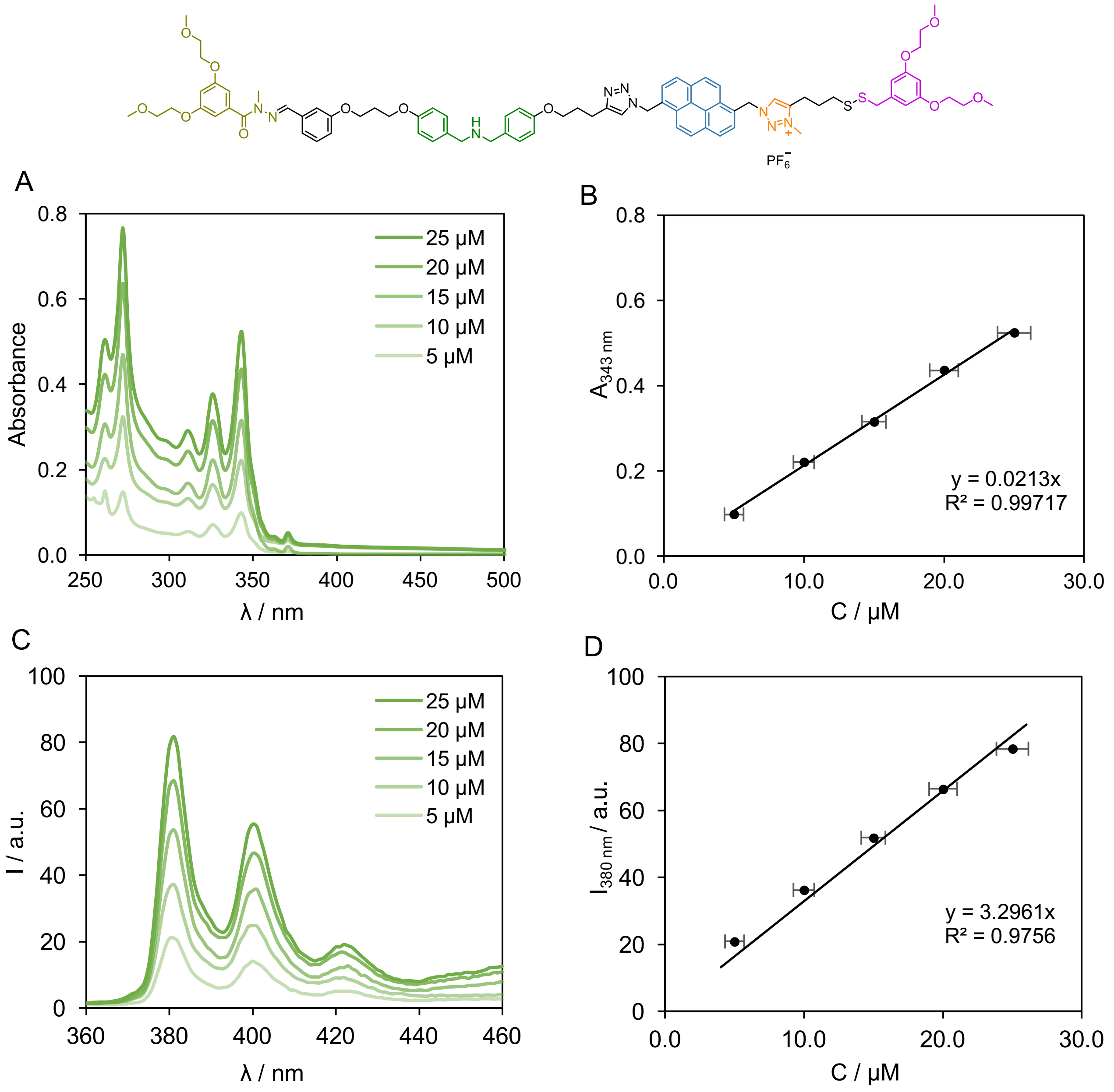


**Figure S7** – (A) UV/Vis spectrum (MeCN) of **1** at different concentrations. (B) Plot of the absorbance of **1** at 343 nm versus concentration, *C*. The molar extinction coefficient ($\varepsilon$) is $5.3 \times 10^4$ L mol$^{-1}$ cm$^{-1}$. (C) Fluorescence emission spectra (MeCN, $\lambda_{ex}$ = 343 nm, $\Delta\lambda_{ex}$ = 5 nm, $\Delta\lambda_{em}$ = 5 nm) of **1** at different concentrations. (D) Plots of emission intensity of **1** at $\lambda_{em\ max}$ = 380 nm versus concentration, *C*.

## 5.2 Fluorescence Emission Spectra of 1·H⁺

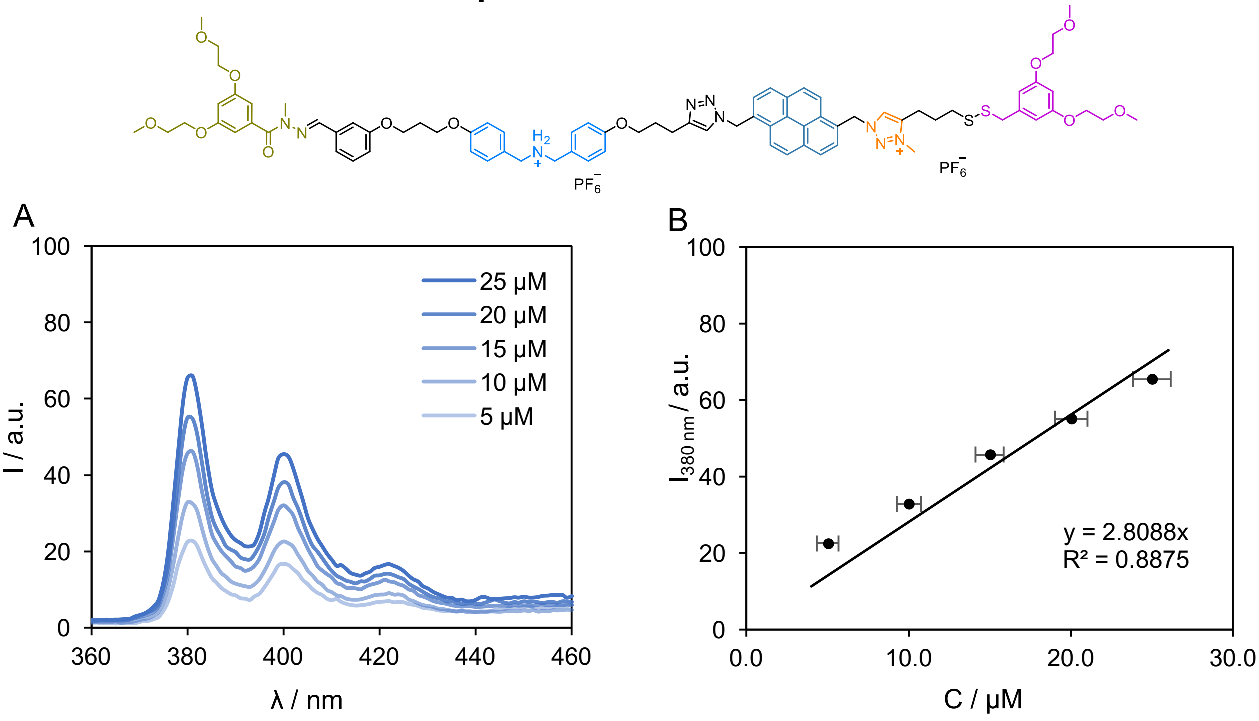


**Figure S8** – (A) Fluorescence emission spectra (MeCN, $\lambda_{ex}$ = 343 nm, $\Delta\lambda_{ex}$ = 5 nm, $\Delta\lambda_{em}$ = 5 nm) of **1**·$H^+$ at different concentrations. (D) Plots of emission intensity of **1**·$H^+$ at $\lambda_{em\ max}$ = 380 nm versus concentration, *C*.

## 5.3 Fluorescence Emission Spectra of *dba*-2·H⁺

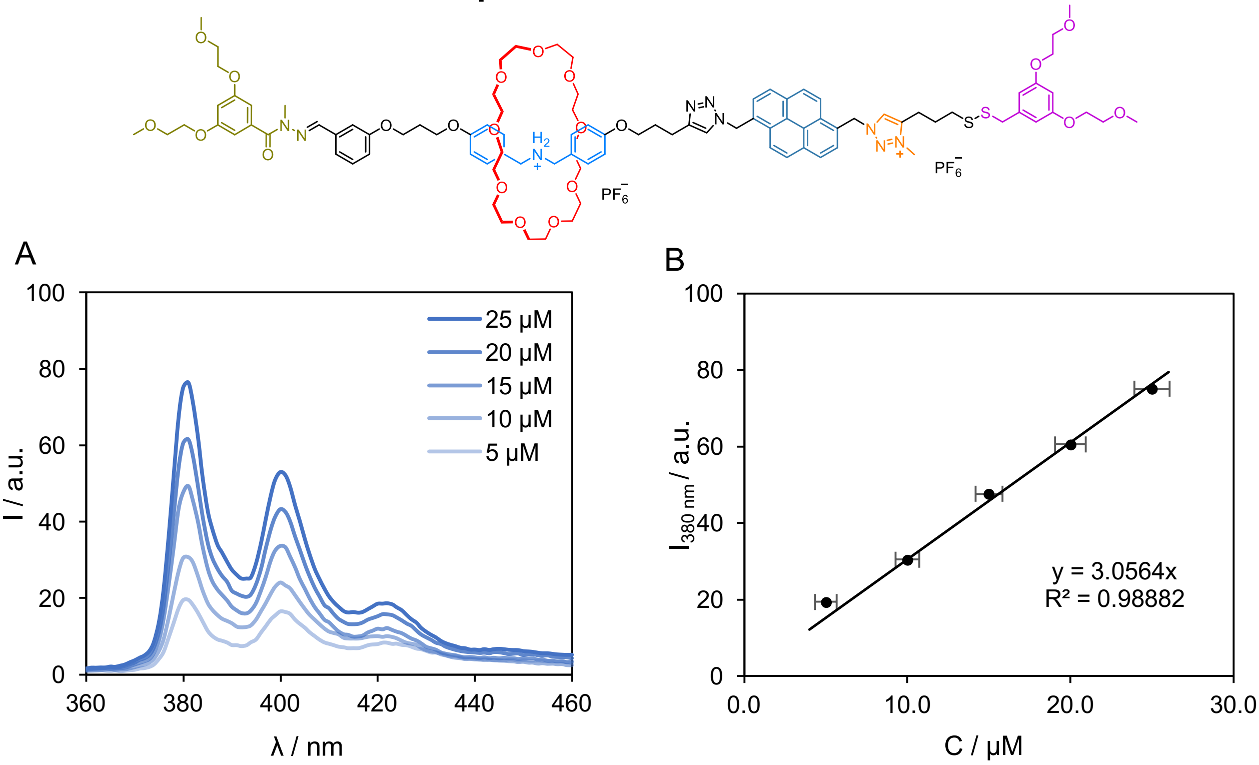


**Figure S9** – (A) Fluorescence emission spectra (MeCN, $\lambda_{ex}$ = 343 nm, $\Delta\lambda_{ex}$ = 5 nm, $\Delta\lambda_{em}$ = 5 nm) of *dba*-**2**·$H^+$ at different concentrations. (D) Plots of emission intensity of *dba*-**2**·$H^+$ at $\lambda_{em\ max}$ = 380 nm versus concentration, *C*.

## 5.4 UV-Vis and Fluorescence Emission Spectra of *mt*-2

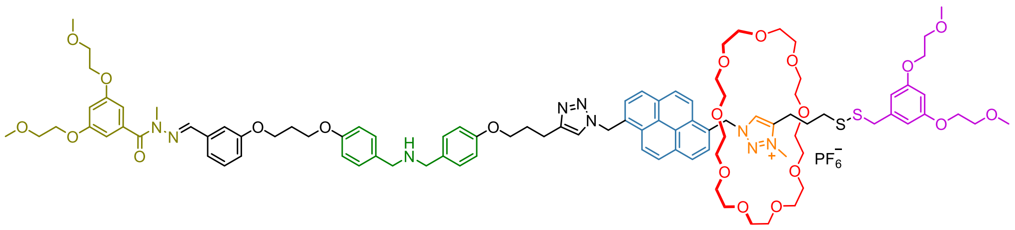

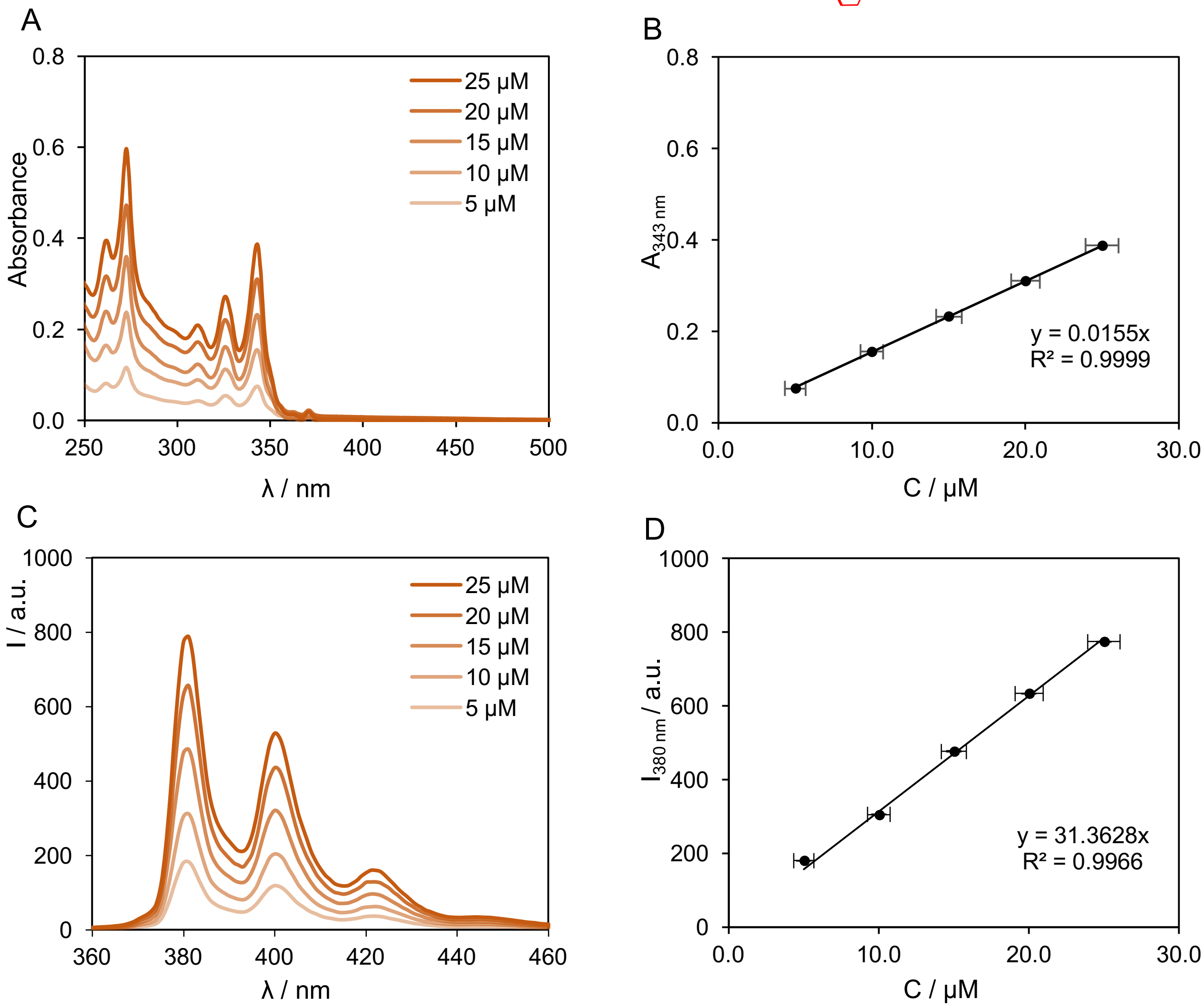


**Figure S10** – (A) UV/Vis spectrum (MeCN) of *mt*-**2** at different concentrations. (B) Plot of the absorbance of *mt*-**2** at 343 nm versus concentration, *C*. The molar extinction coefficient ($\varepsilon$) is 3.9 × $10^4$ L $mol^{-1}$ $cm^{-1}$. (C) Fluorescence emission spectra (MeCN, $\lambda_{ex}$ = 343 nm, $\Delta\lambda_{ex}$ = 5 nm, $\Delta\lambda_{em}$ = 5 nm) of *mt*-**2** at different concentrations. (D) Plots of emission intensity of *mt*-**2** at $\lambda_{em\ max}$ = 380 nm versus concentration, *C*.

## 5.5 Fluorescence Emission Spectra During the Switching of *mt*-2 to *dba*-2·H+ Under Transient Acidic Conditions

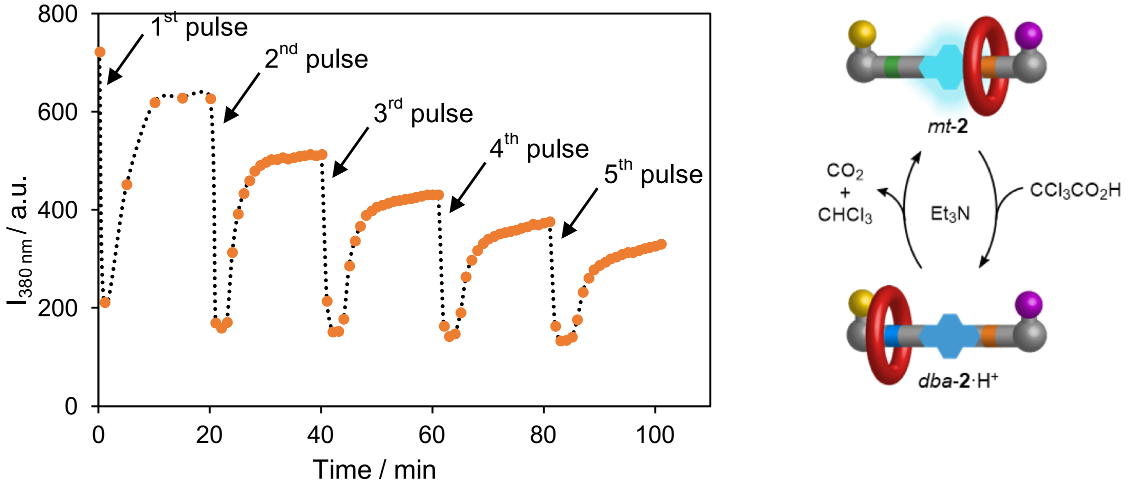


**Figure S11** – Fluorescence emission spectra (MeCN, $\lambda_{ex}$ = 343 nm, $\Delta\lambda_{ex}$ = 5 nm, $\Delta\lambda_{em}$ = 5 nm) during the switching between *mt*-**2** and *dba*-**2**·$H^+$ under transient acidic conditions. Reagents and conditions: *mt*-**2** (22 μM), $Et_3N$ (100 equiv.), *then* 10 M $CCl_3CO_2H$ in MeCN (110 eq. per pulse). *Note: some dethreading is observed after each pulse of $CCl_3CO_2H$ due to hydrolysis of the hydrazone stopper under high dilution conditions.

## 5.6 Fluorescence Emission Spectra During the Stepwise Threading and Dethreading Processes

1. Procedure for threading study: **1**·H$^+$ (2.0 mg, 1.2 µmol), $Et_3N$ (8.4 µL, 60 µmol, 50 equiv.), $PhNH_2$ (0.1 M in $CD_3CN$, 6 µL, 0.6 µmmol, 0.5 equiv.), 27C9 (9.5 mg, 24 µmol, 20 equiv.) and hydrazone **3** (1.5 mg, 3.6 µmol, 3.0 equiv.) were dissolved $CD_3CN$ (600 µL). To this, 10 M $CCl_3CO_2H$ in $CD_3CN$ (24 µL, 240 µmol, 200 equiv.) was added. Aliquots were sampled after 7 hours, 24 hours and then each 24 hours over the course of 6 days. Upon collection, samples were basified with $Et_3N$, diluted with MeCN (up to 1.5 mL) and analysed by fluorescence spectroscopy (MeCN, $\lambda_{ex}$ = 343 nm, $\Delta\lambda_{ex}$ = 5 nm, $\Delta\lambda_{em}$ = 5 nm).

2. Procedure for dethreading study: To the samples prepared previously, thiol **4** (50 equiv.) and disulfide **5** (500 equiv.) were added and the samples were continuously analysed by fluorescence spectroscopy (MeCN, $\lambda_{ex}$ = 343 nm, $\Delta\lambda_{ex}$ = 5 nm, $\Delta\lambda_{em}$ = 5 nm) until they reached a minimum intensity (assumed to be the point of complete dethreading).

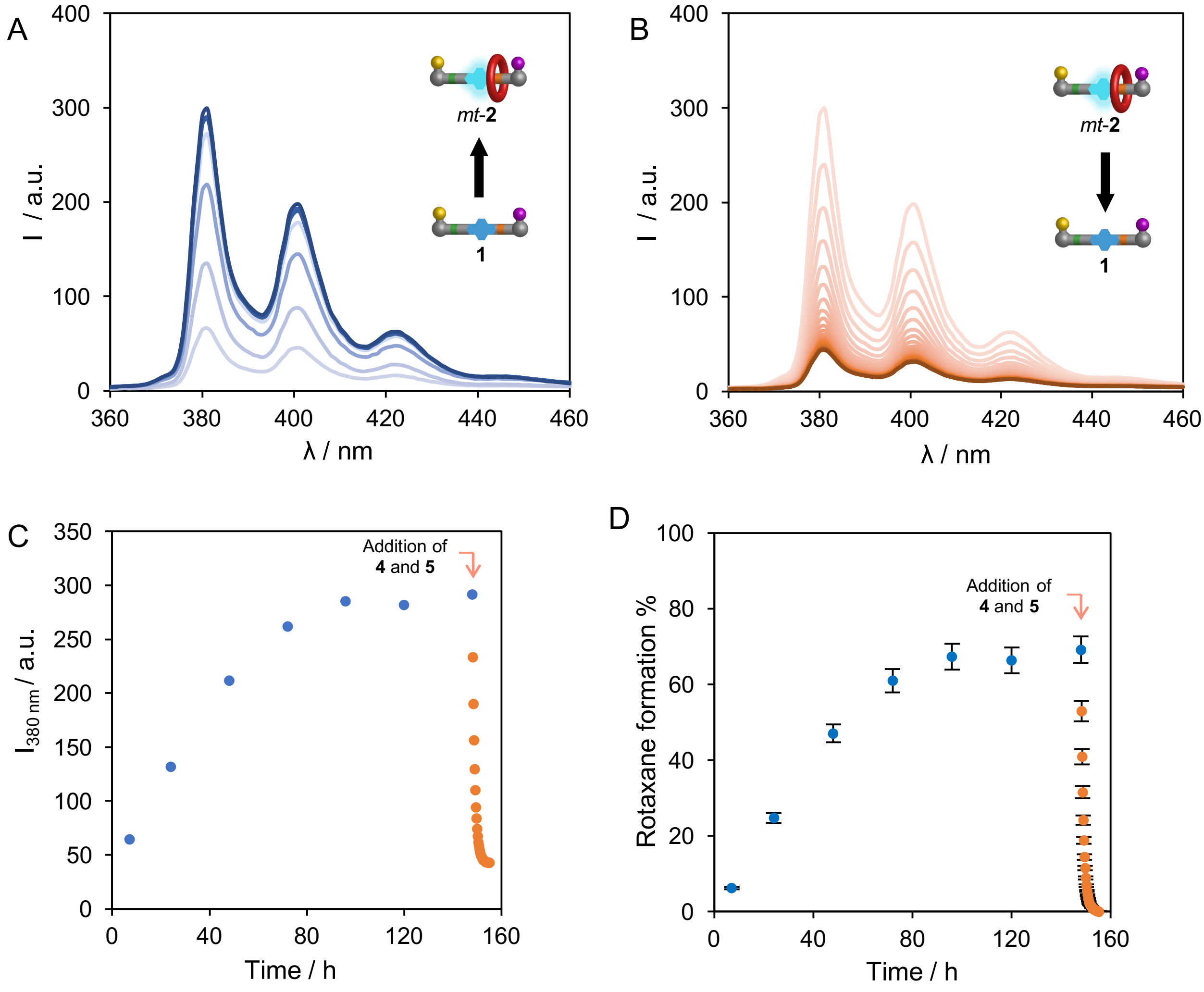


**Figure S12** – (A) Fluorescence spectra of the threading study normalized for concentration. (B) Fluorescence spectra of the *in situ* dethreading of the sample collected after 6 days. (C) Plot showing the evolution in fluorescence intensity at $\lambda_{em\ max}$ = 380 nm during the threading (**blue**) and dethreading (**orange**) steps. (D) Plot showing the rotaxane percentage in solution during the threading and dethreading steps.

## 5.7 Fluorescence Emission Spectra During the Pulsed Fuel Operation of 1

Procedure for the repetitive operation, analyzed by fluorescence spectroscopy (Figure S13-14):

To a solution of **1**·H$^+$ (1.4 mg, 0.8 μmol) in $CD_3CN$ (600 μL), $Et_3N$ (5.6 μL, 40 μmol, 50 equiv.), $PhNH_2$ (0.1 M in $CD_3CN$, 4 μL, 0.4 μmol, 0.5 equiv.), 27C9 (6.2 mg, 16 μmol, 20 equiv.), hydrazone **3** (1.0 mg, 2.4 μmol, 3.0 equiv.), thiol **4** (0.4 mg, 1.6 μmol, 2.0 equiv.) and disulfide **5** (8.7 mg, 16 μmol, 20 equiv.) were added. An aliquot was collected before addition of $CCl_3CO_2H$, and the sample was analysed by fluorescence spectroscopy (MeCN, $\lambda_{ex}$ = 343 nm, $\Delta\lambda_{ex}$ = 5 nm, $\Delta\lambda_{em}$ = 5 nm), showing the starting point corresponding to a mixture of thread **1** and 27C9 (**Black**). Then, a pulse of $CCl_3CO_2H$ (100, 150 or 200 equiv.) was added to the reaction mixture, and the evolution of the threading, as well as decarboxylation of the $CCl_3CO_2H$, were monitored by $^1$H NMR. Immediately after full decarboxylation of the acid, another aliquot was collected and analysed by fluorescence spectroscopy, corresponding the point of maximum threading (**Blue**). To this aliquot, excess thiol **4** (50 equiv.) and disulfide **5** (500 equiv.) were added to speed up dethreading, which was continuously analyzed by fluorescence spectroscopy (**Orange**), until a minimum intensity was observed (assumed to be the point of complete dethreading).

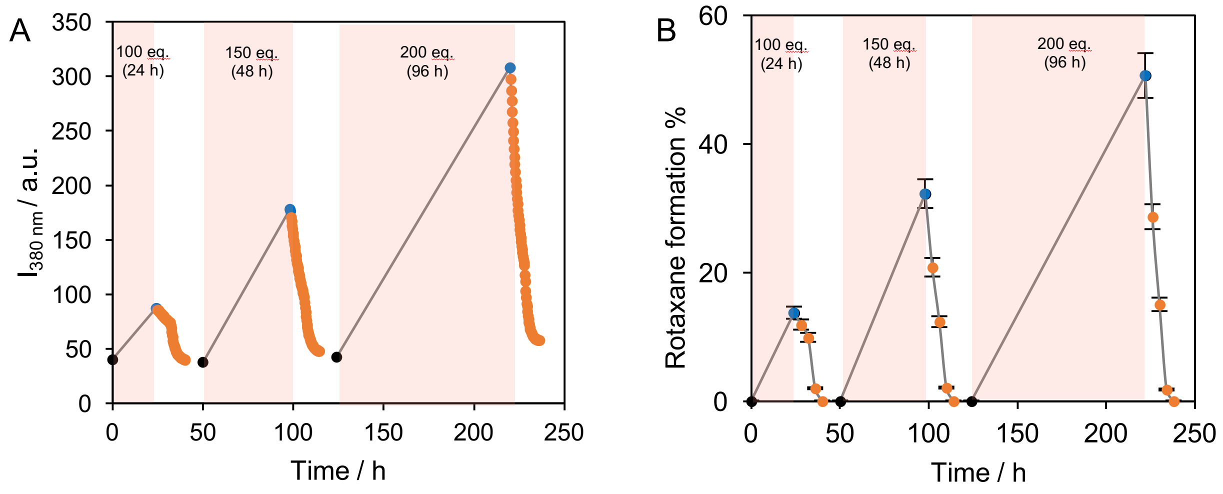


**Figure S13** – (A) Plot showing the evolution in fluorescence intensity at $\lambda_{em\ max}$ = 380 nm over three pulses of $CCl_3CO_2H$ of 100, 150 and 200 equiv. respectively. **Black** – sample before addition of the $CCl_3CO_2H$; **Blue** – sample immediately after full decarboxylation of $CCl_3CO_2H$; **Orange** – dethreading of the [2]rotaxane molecules *in situ* by addition of excess thiol **4** (50 equiv.) and disulfide **5** (500 equiv.). (B) Plot showing the rotaxane percentage in solution during the three operation cycles.

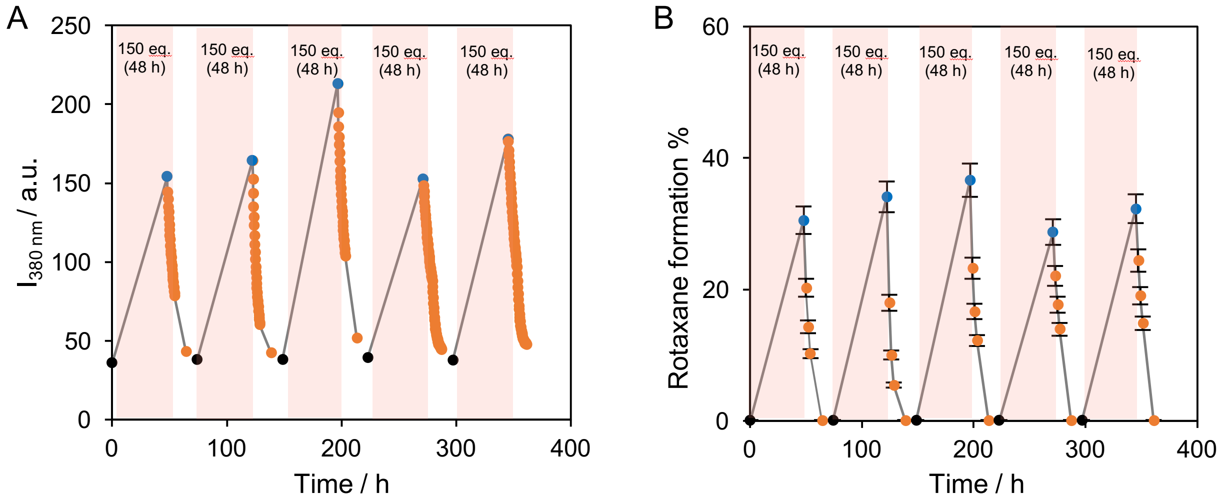


**Figure S14** – (A) Plot showing the evolution in fluorescence intensity at $\lambda_{em\ max}$ = 380 nm over five consecutive pulses of $CCl_3CO_2H$ (150 equiv.) in one pot. **Black** – sample before addition of the $CCl_3CO_2H$; **Blue** – sample immediately after full decarboxylation of $CCl_3CO_2H$; **Orange** – dethreading of the [2]rotaxane molecules *in situ* by addition of excess thiol **4** (50 equiv.) and disulfide **5** (500 equiv.). (B) Plot showing the rotaxane percentage in solution during the five operation cycles.

# 6 $^1$H & $^{13}$C NMR Spectra

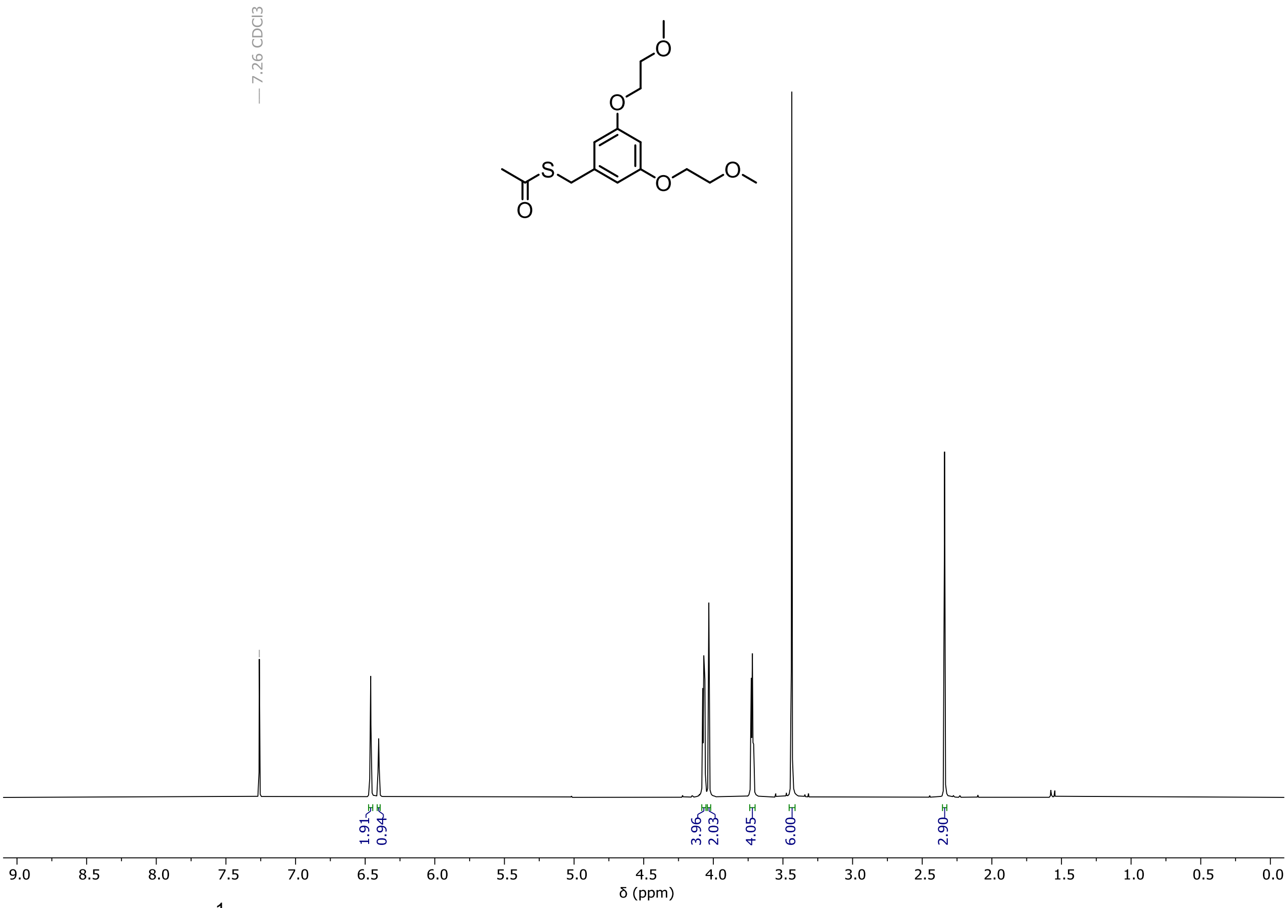


**Spectrum S1** – $^1$H NMR (600 MHz, 298 K, Chloroform-*d*) spectrum of **S2**.

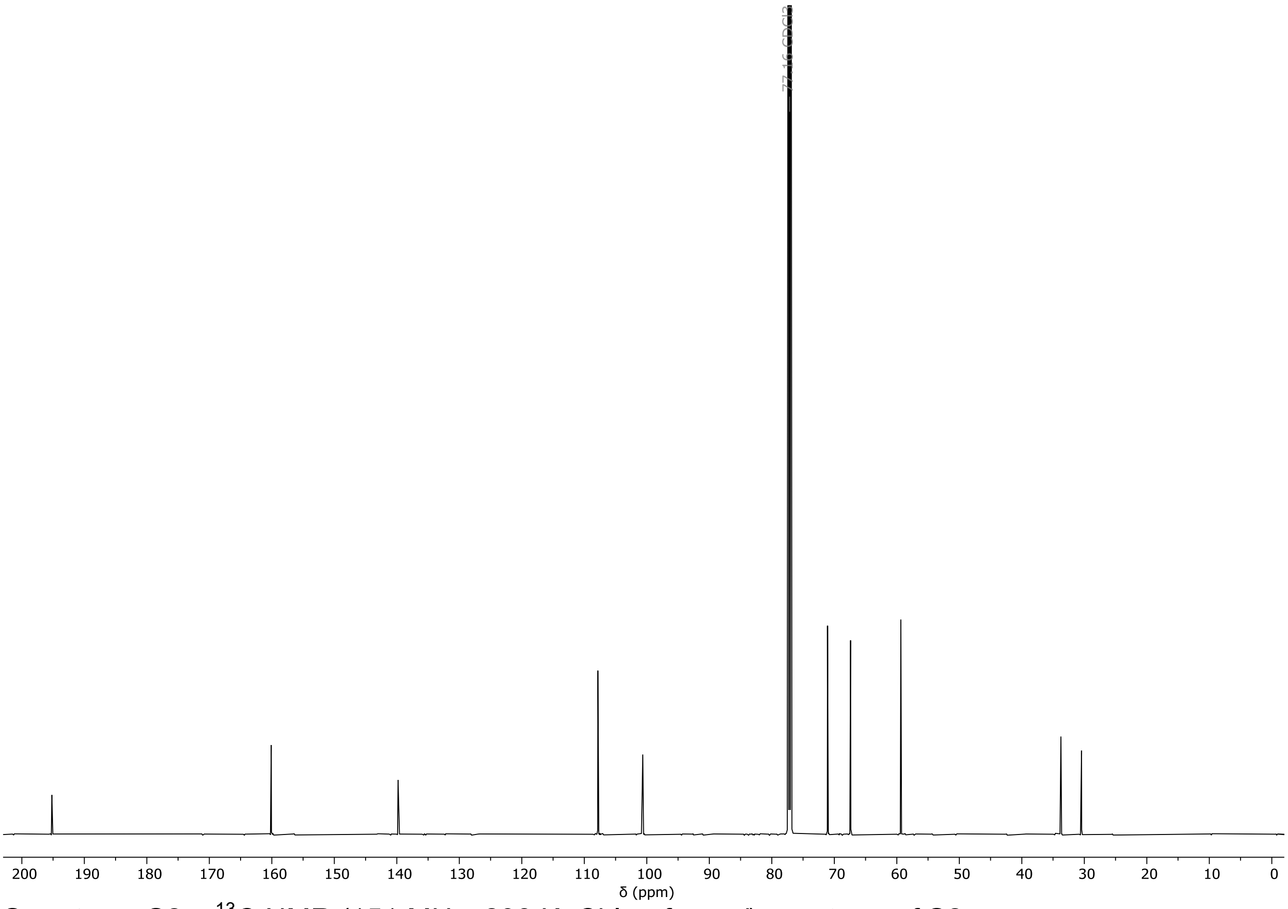


**Spectrum S2** – $^{13}$C NMR (151 MHz, 298 K, Chloroform-*d*) spectrum of **S2**.

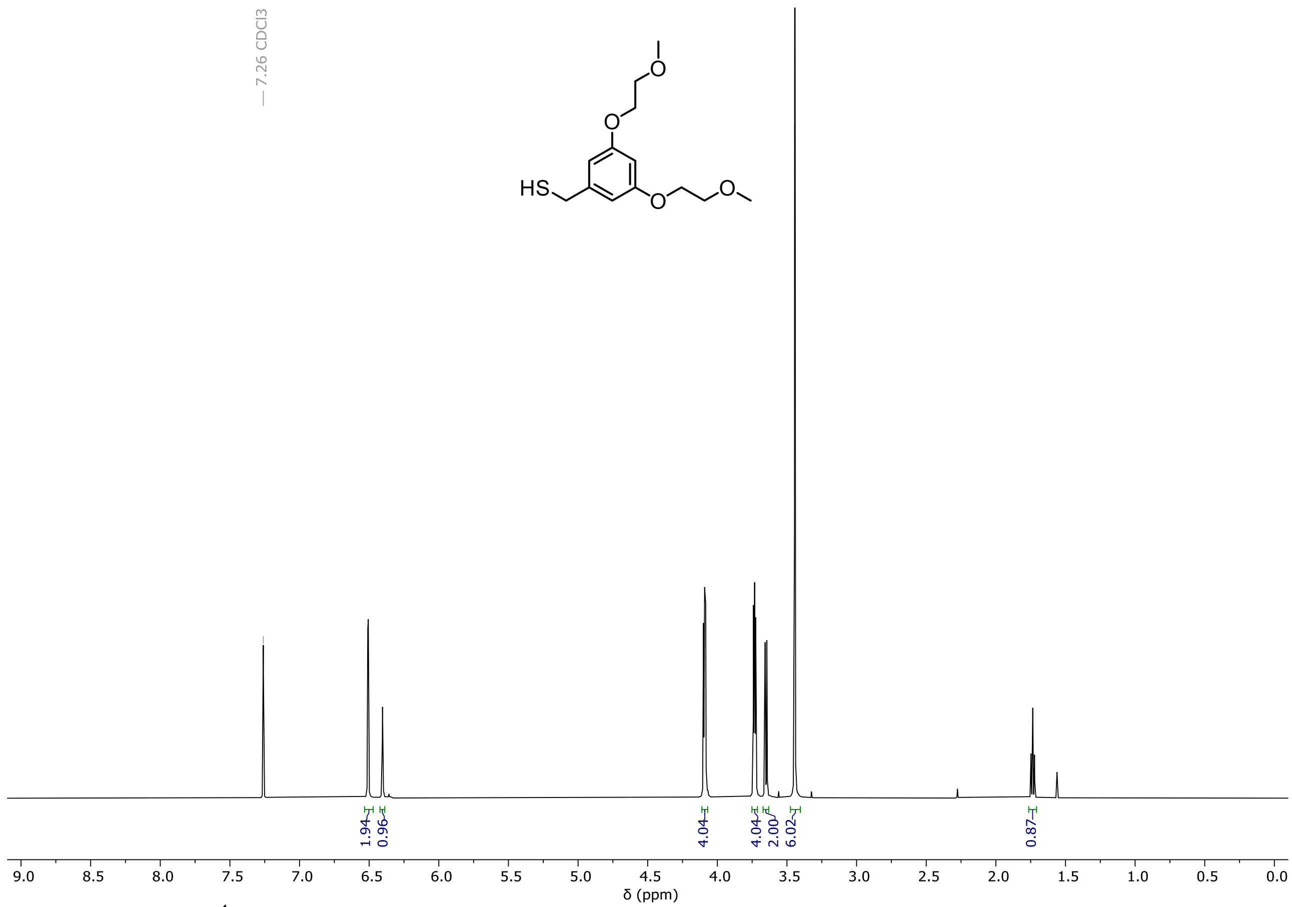


**Spectrum S3** – $^{1}$H NMR (600 MHz, 298 K, Chloroform-*d*) spectrum of **4**.

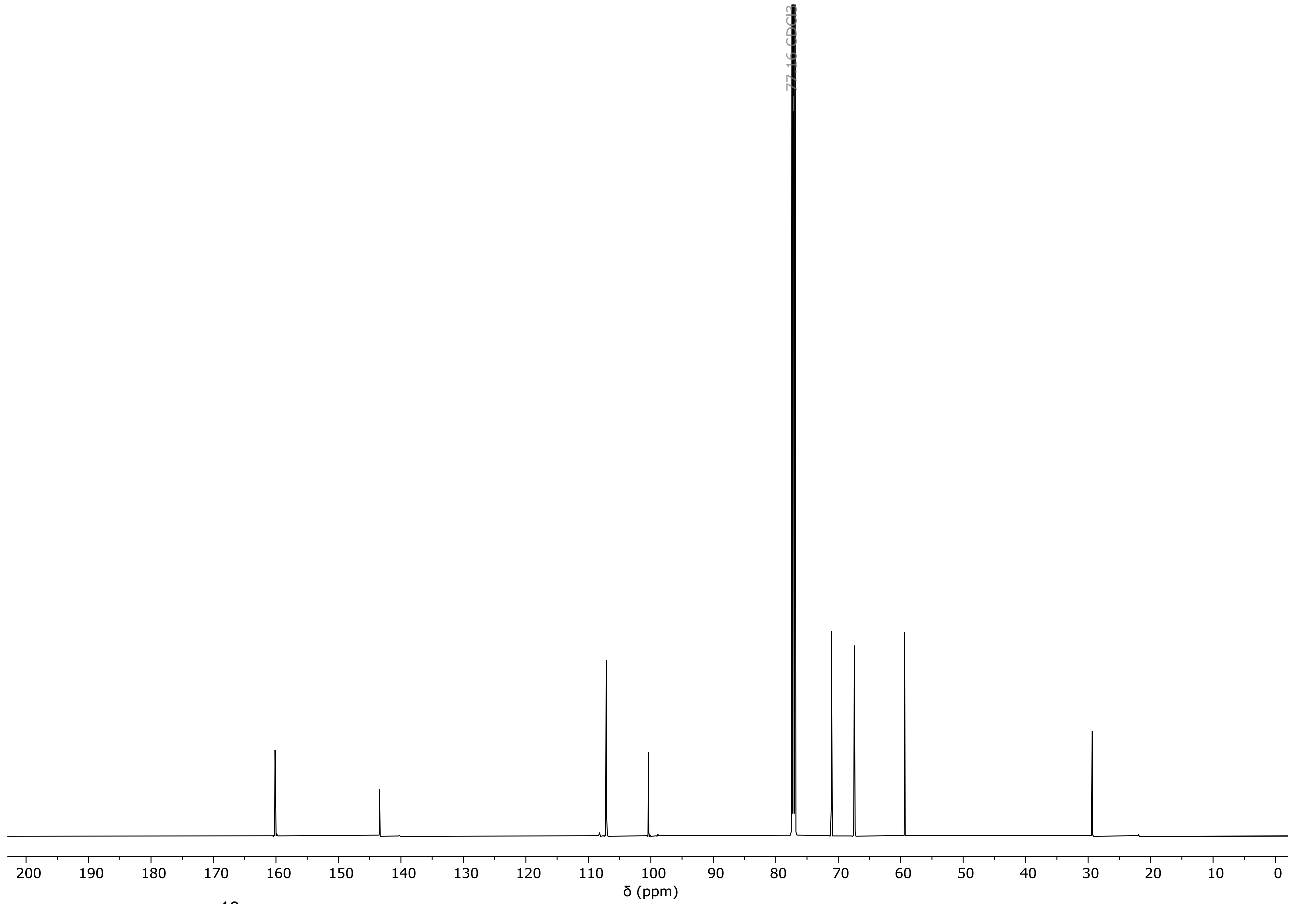


**Spectrum S4** – $^{13}$C NMR (151 MHz, 298 K, Chloroform-*d*) spectrum of **4**.

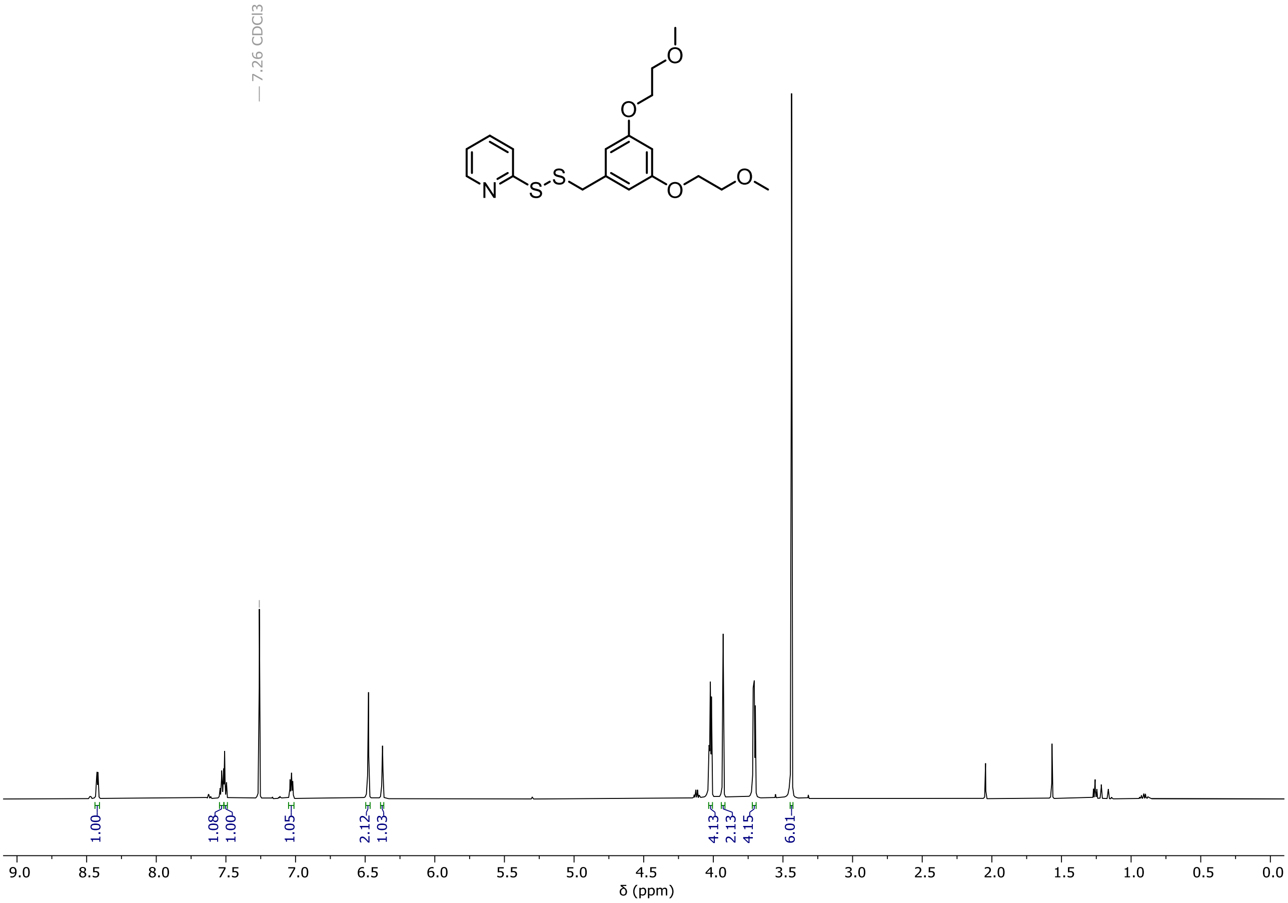


**Spectrum S5** – 1H NMR (600 MHz, 298 K, Chloroform-*d*) spectrum of **S3**.

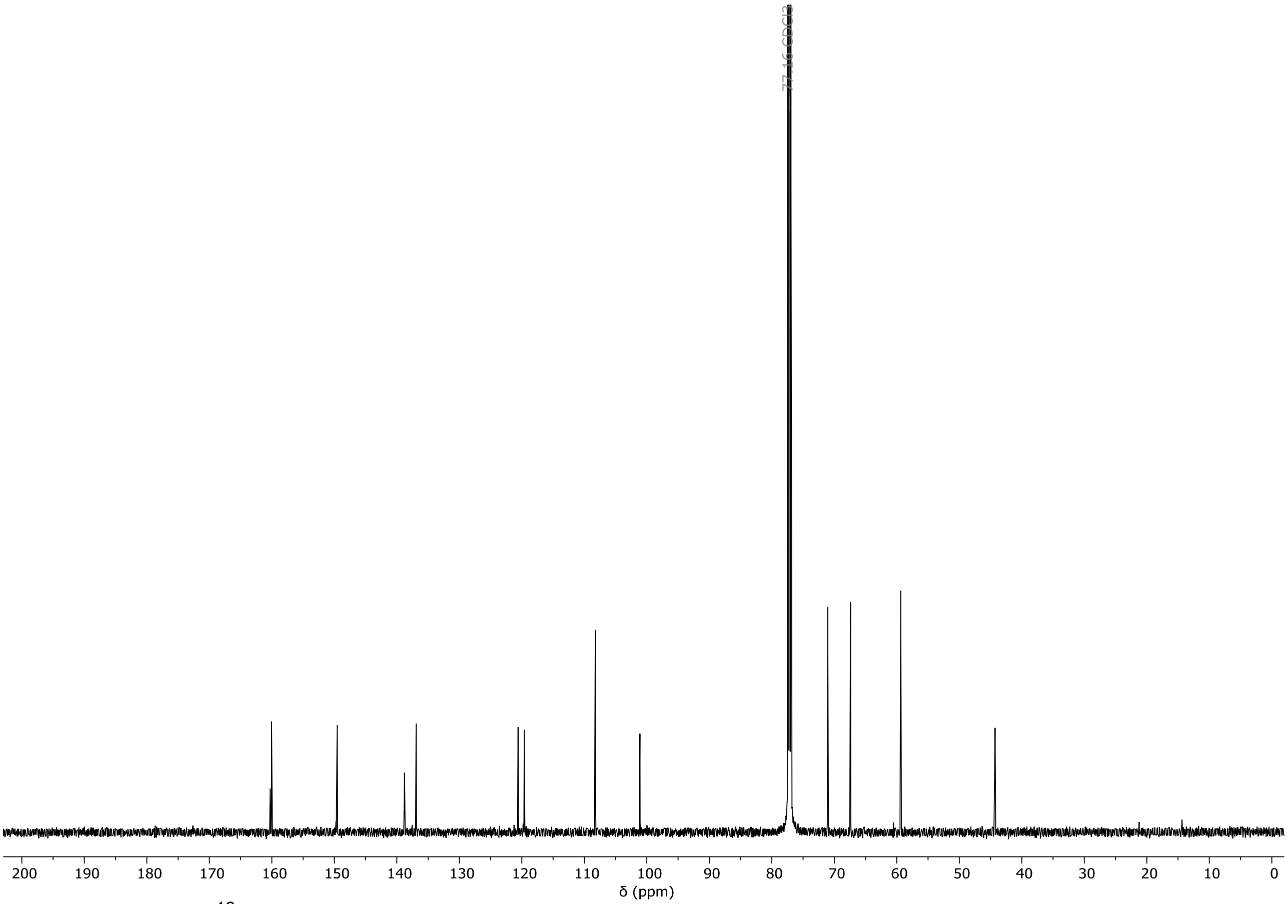


**Spectrum S6** – $^{13}$C NMR (151 MHz, 298 K, Chloroform-*d*) spectrum of **S3**.

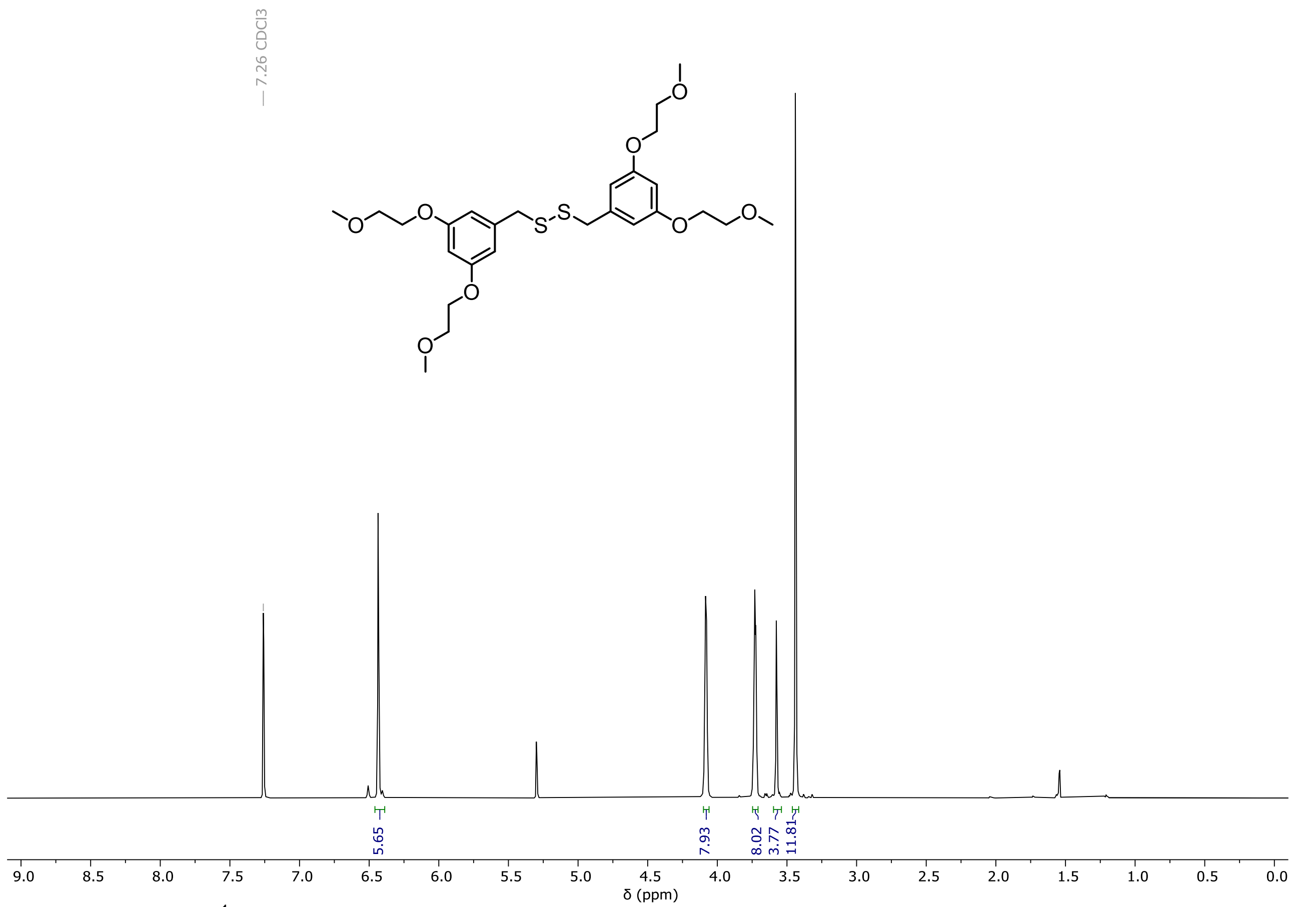


**Spectrum S7** – $^{1}$H NMR (600 MHz, 298 K, Chloroform-*d*) spectrum of **5**.

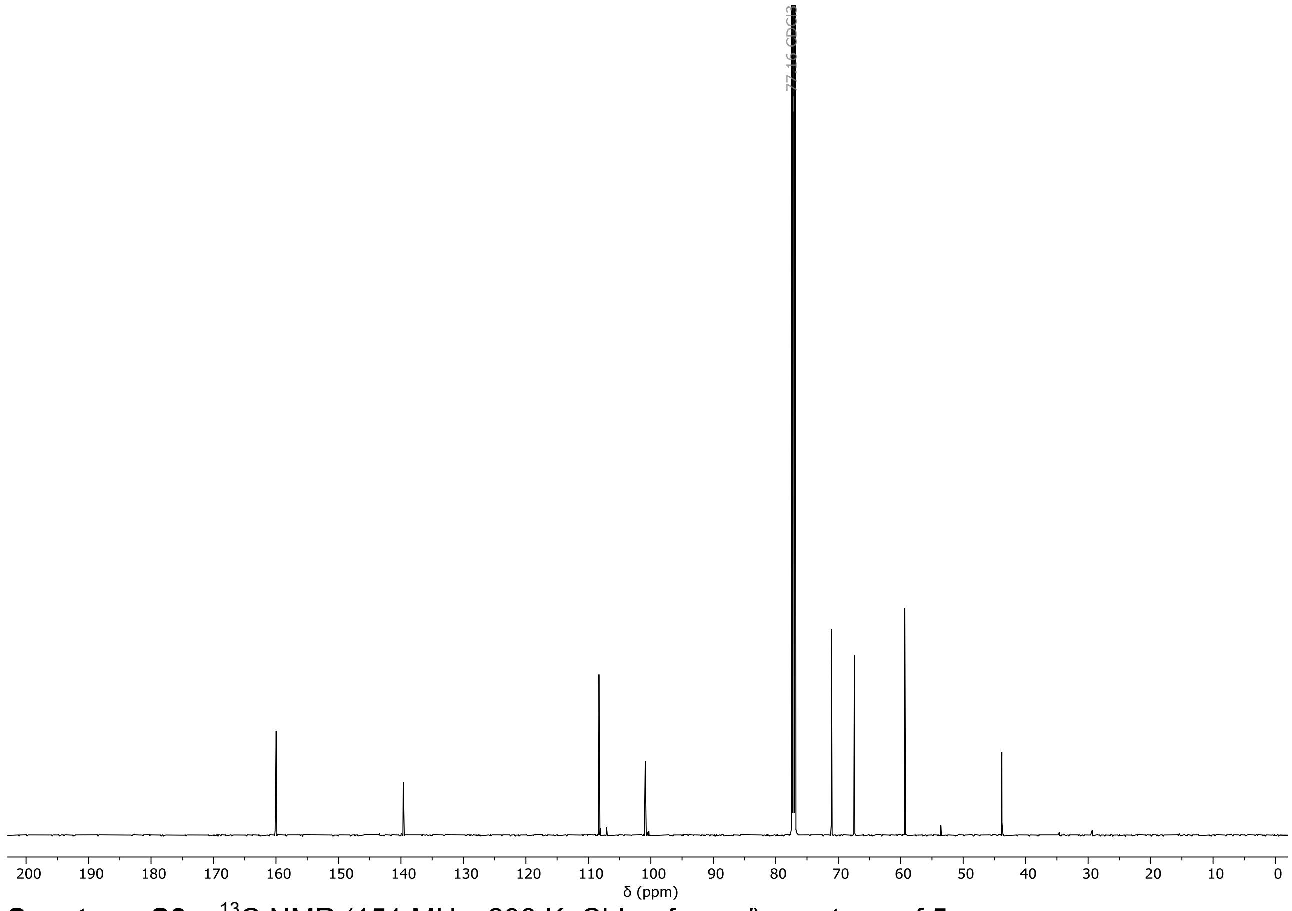


**Spectrum S8** – $^{13}$C NMR (151 MHz, 298 K, Chloroform-*d*) spectrum of **5**.

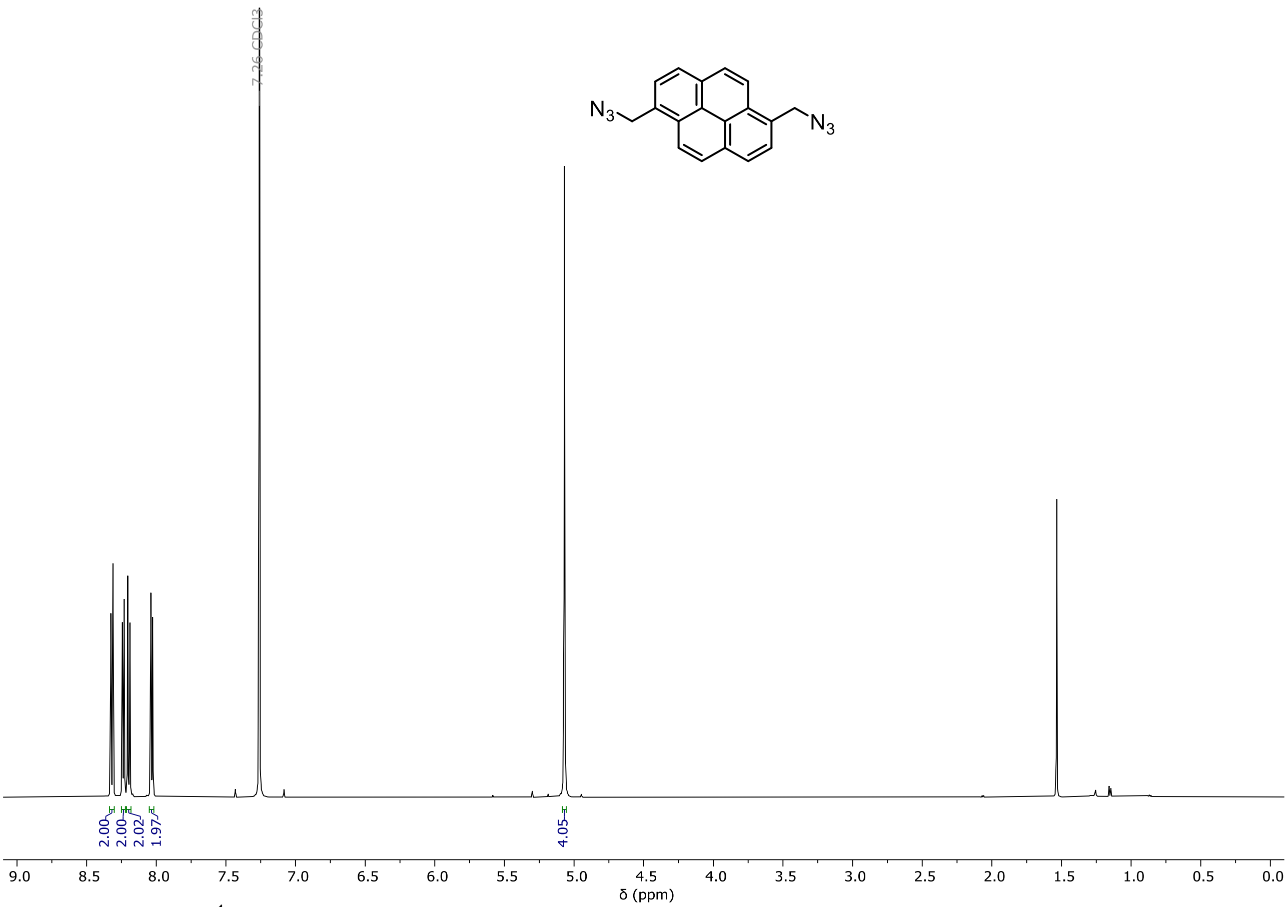


**Spectrum S9** – $^{1}$H NMR (600 MHz, 298 K, Chloroform-*d*) spectrum of **S5**.

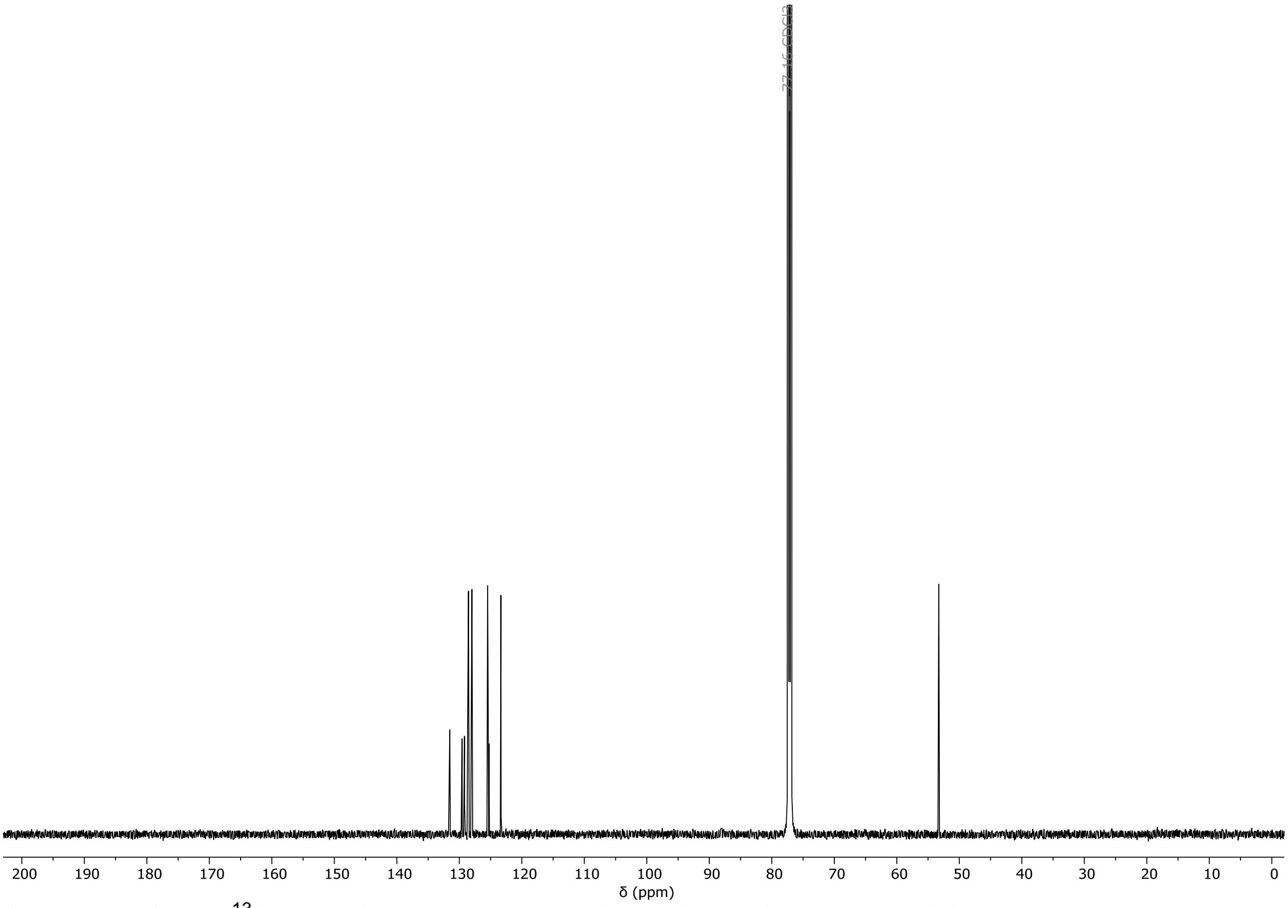


**Spectrum S10** – $^{13}$C NMR (151 MHz, 298 K, Chloroform-*d*) spectrum of **S5**.

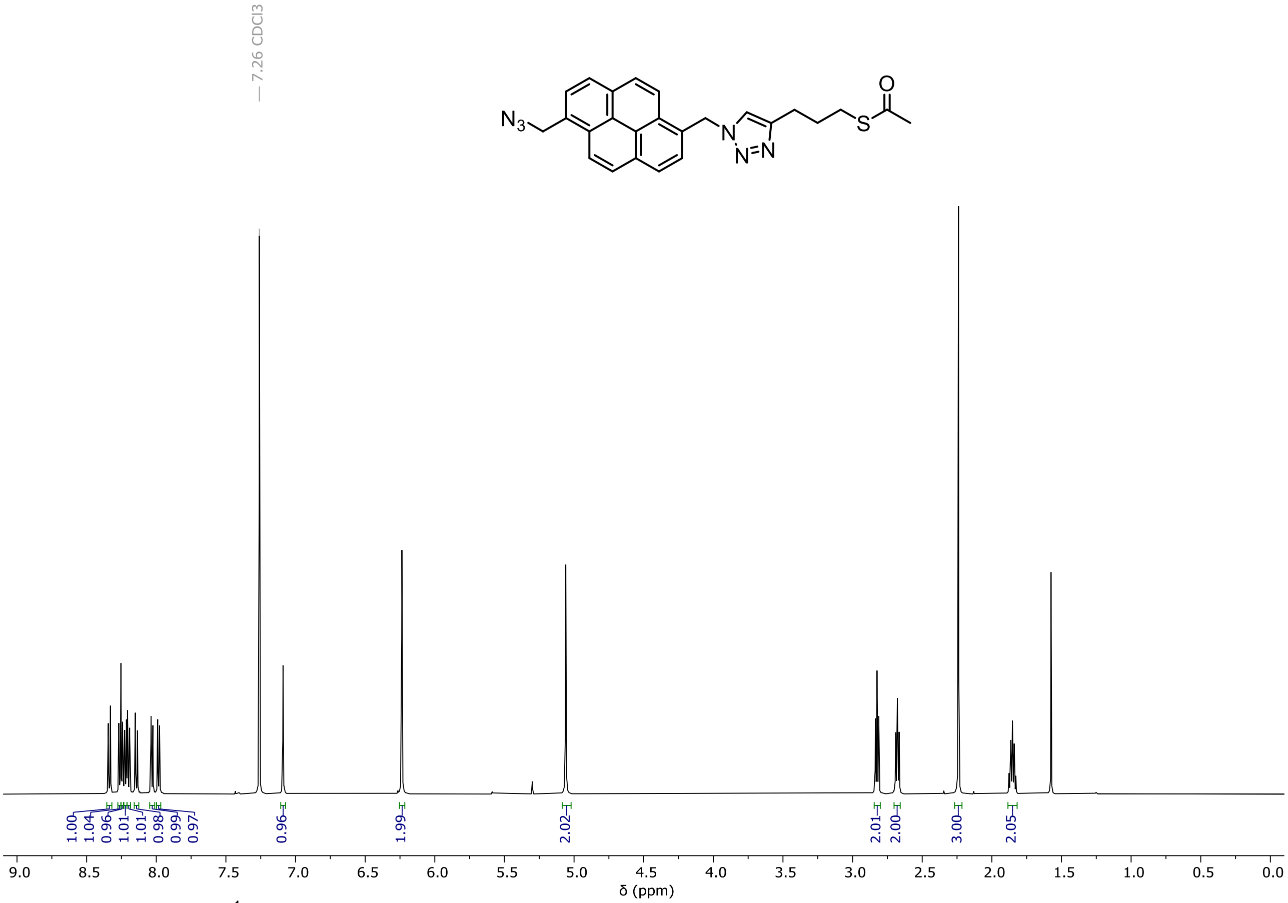


**Spectrum S11** – $^{1}$H NMR (600 MHz, 298 K, Chloroform-*d*) spectrum of **S7**.

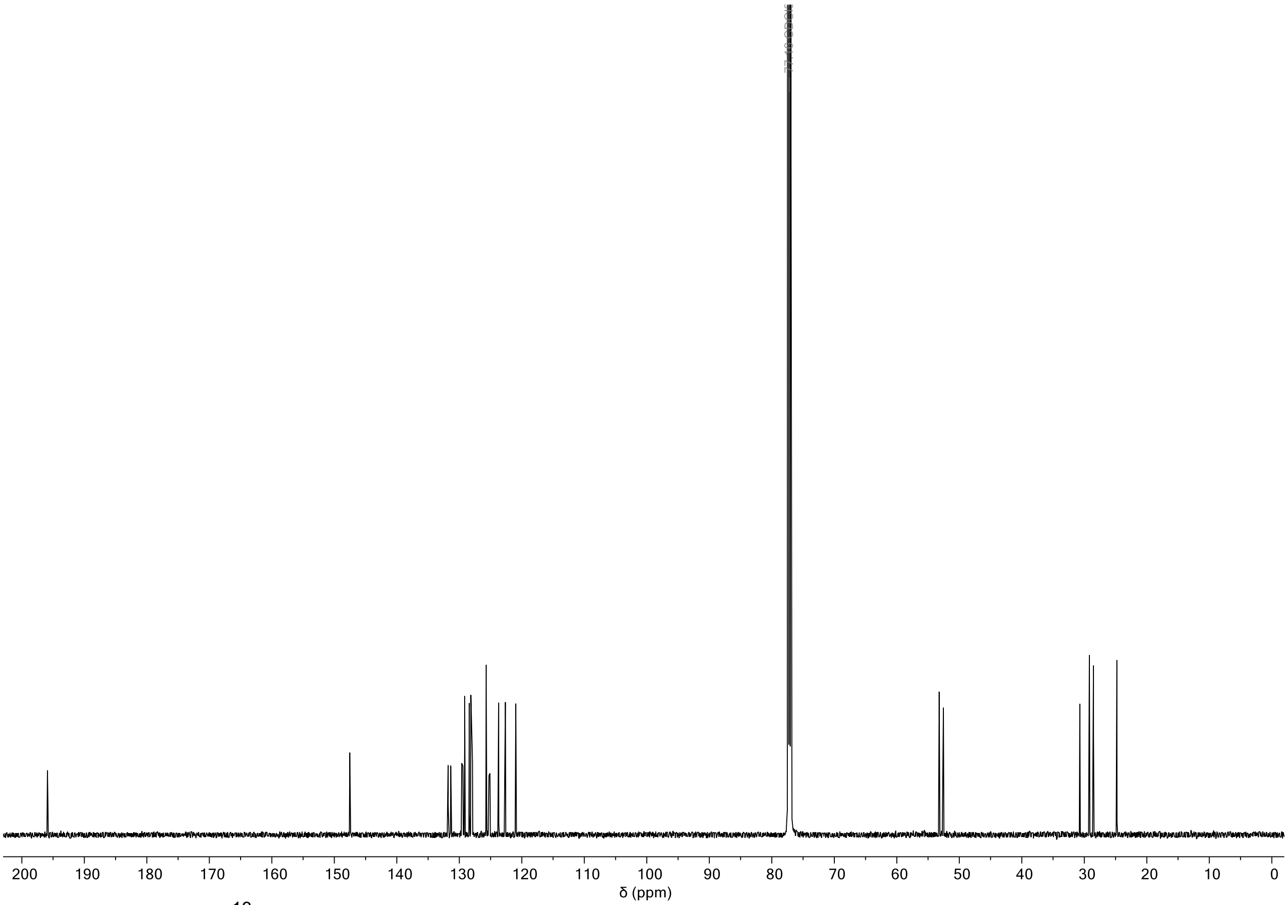


**Spectrum S12** – $^{13}$C NMR (151 MHz, 298 K, Chloroform-*d*) spectrum of **S7**.

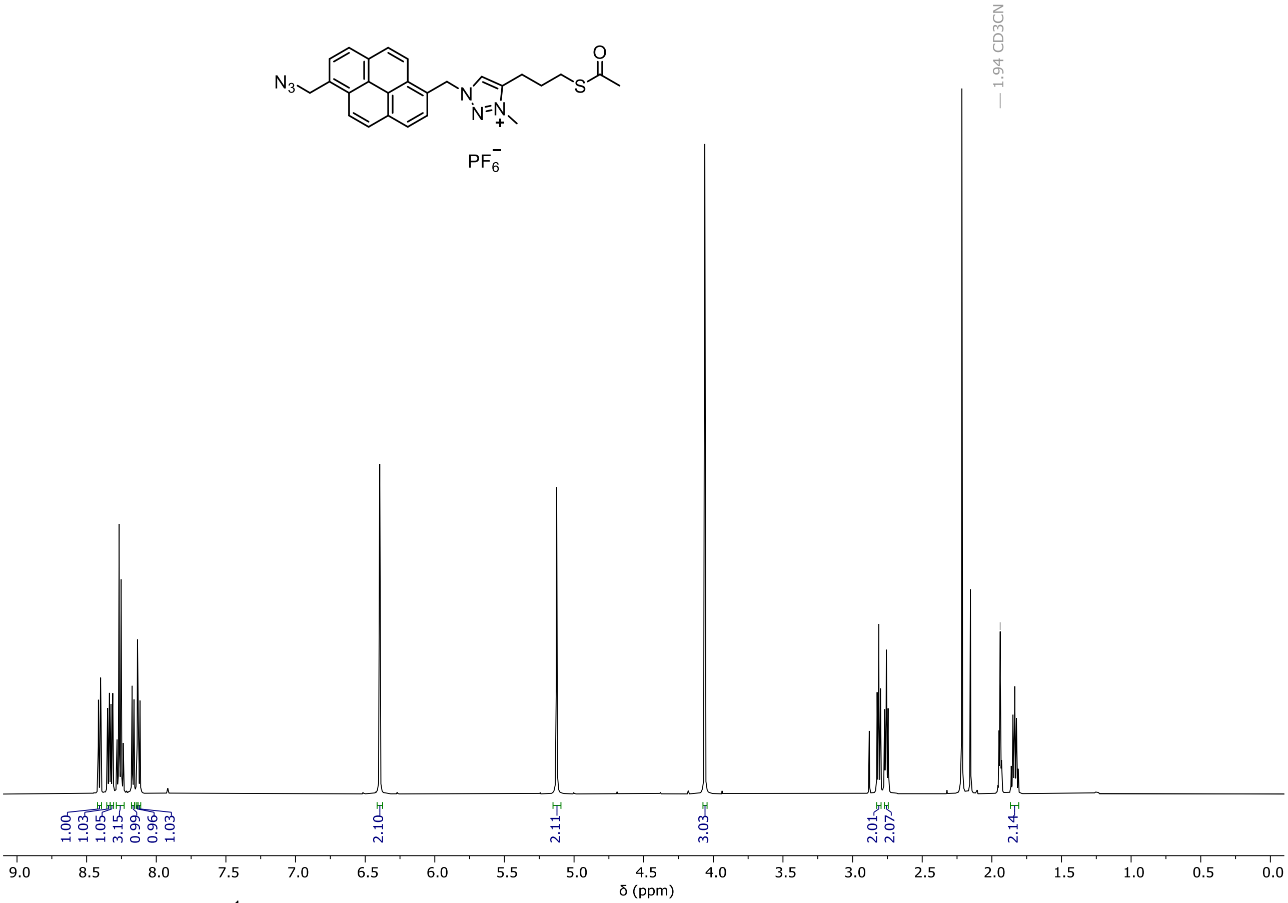


**Spectrum S13** – ¹H NMR (600 MHz, 298 K, Acetonitrile-$d_3$) spectrum of **S8**.

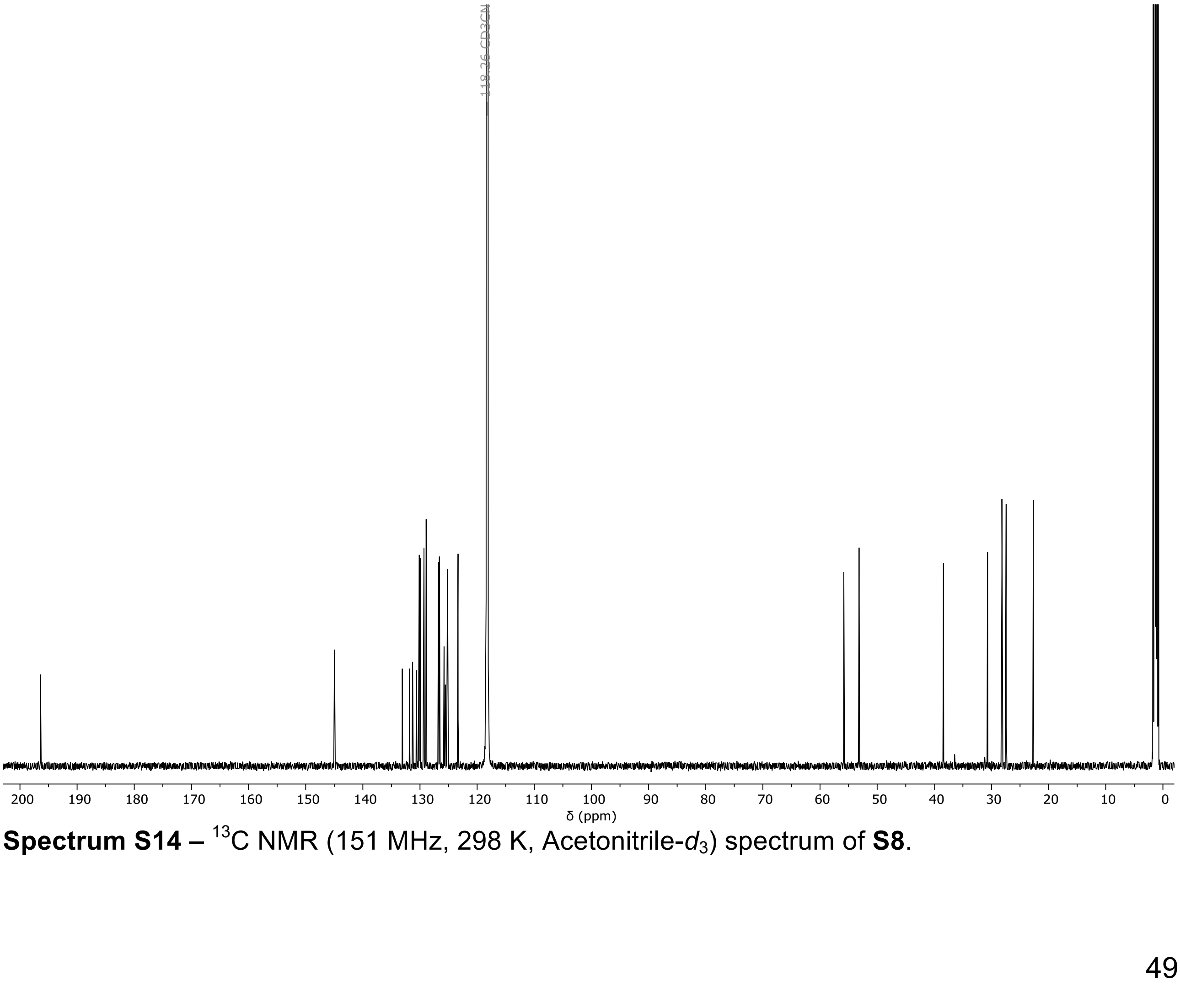


**Spectrum S14** – ¹³C NMR (151 MHz, 298 K, Acetonitrile-$d_3$) spectrum of **S8**.

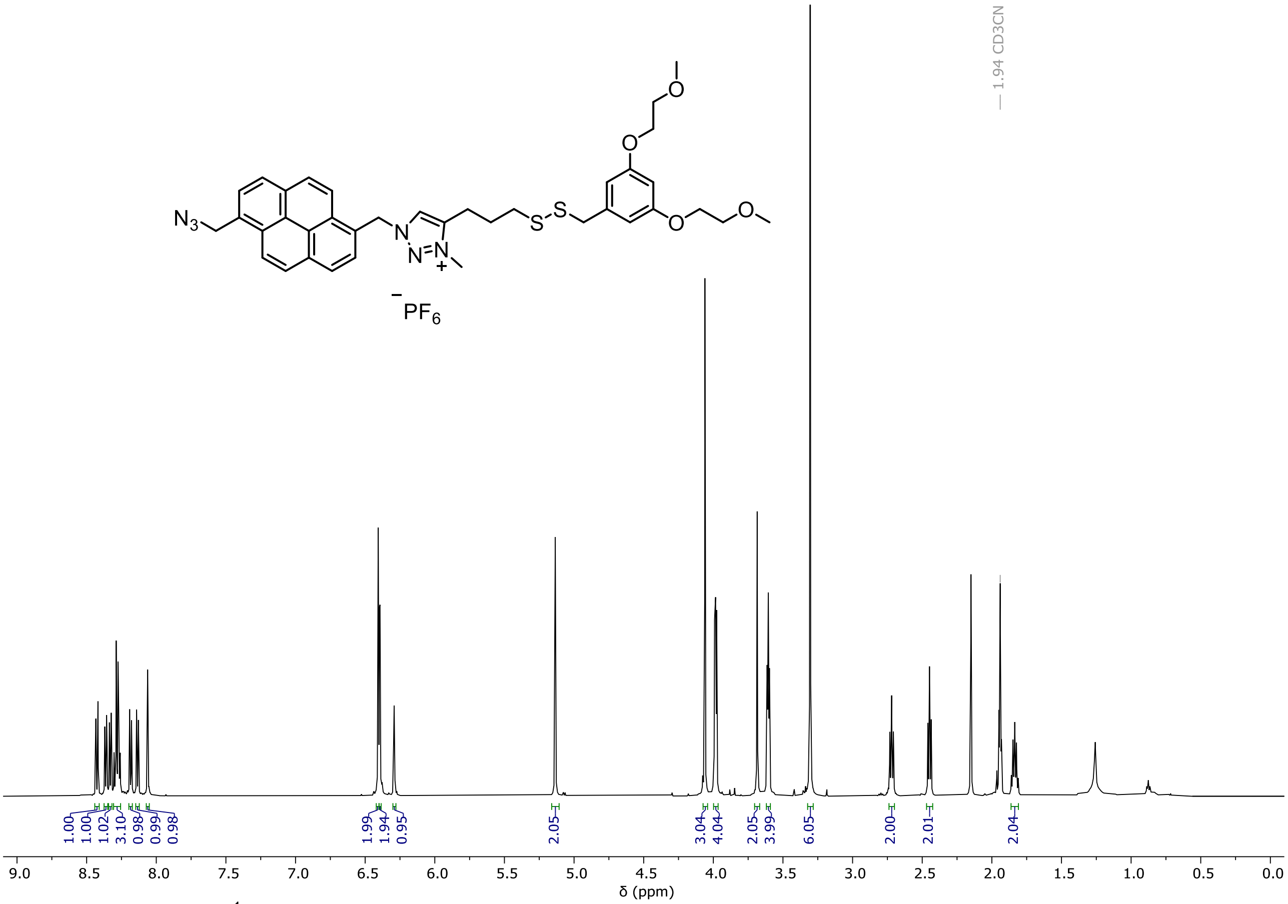


**Spectrum S15** – $^{1}$H NMR (600 MHz, 298 K, Acetonitrile-$d_3$) spectrum of **S9**.

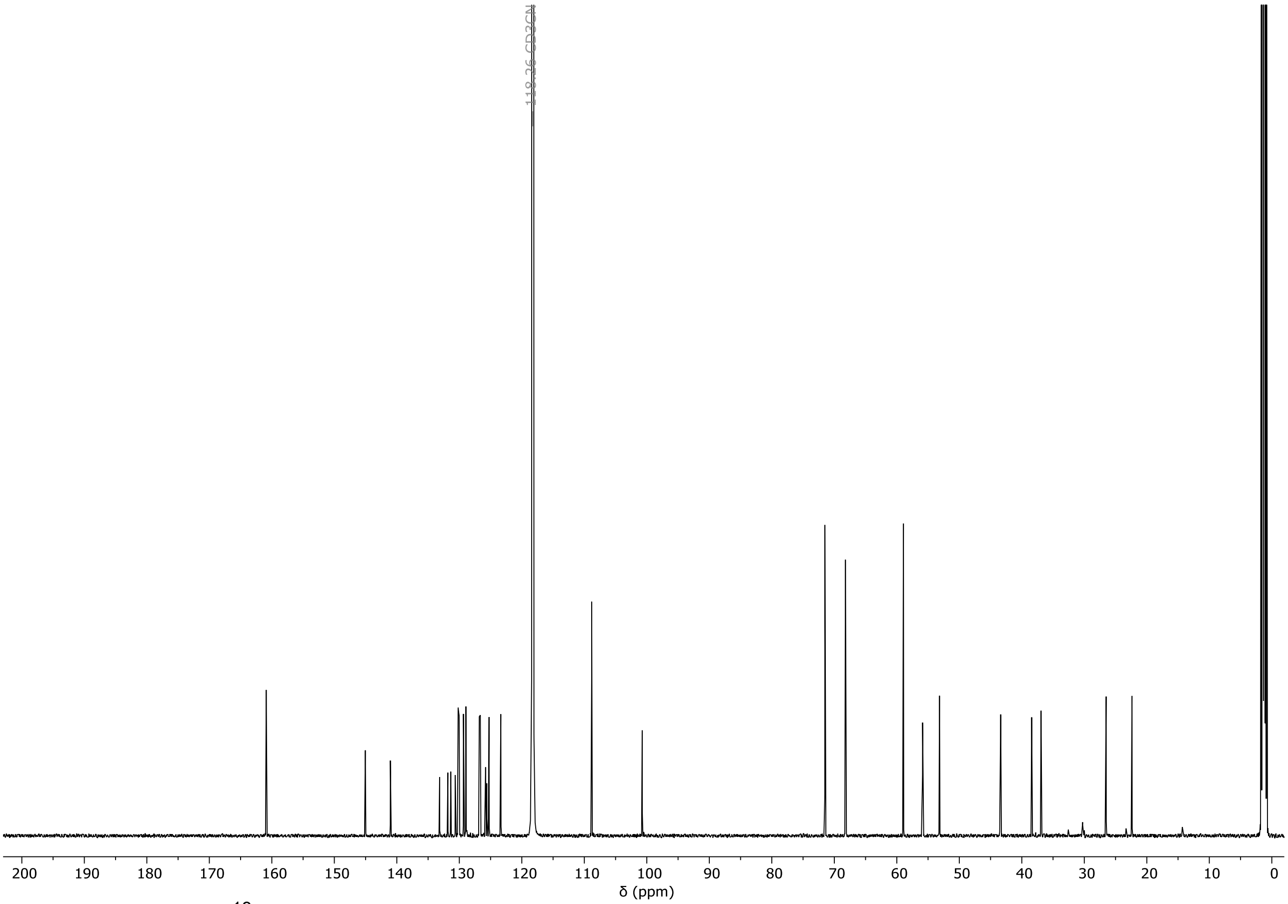


**Spectrum S16** – $^{13}$C NMR (151 MHz, 298 K, Acetonitrile-$d_3$) spectrum of **S9**.

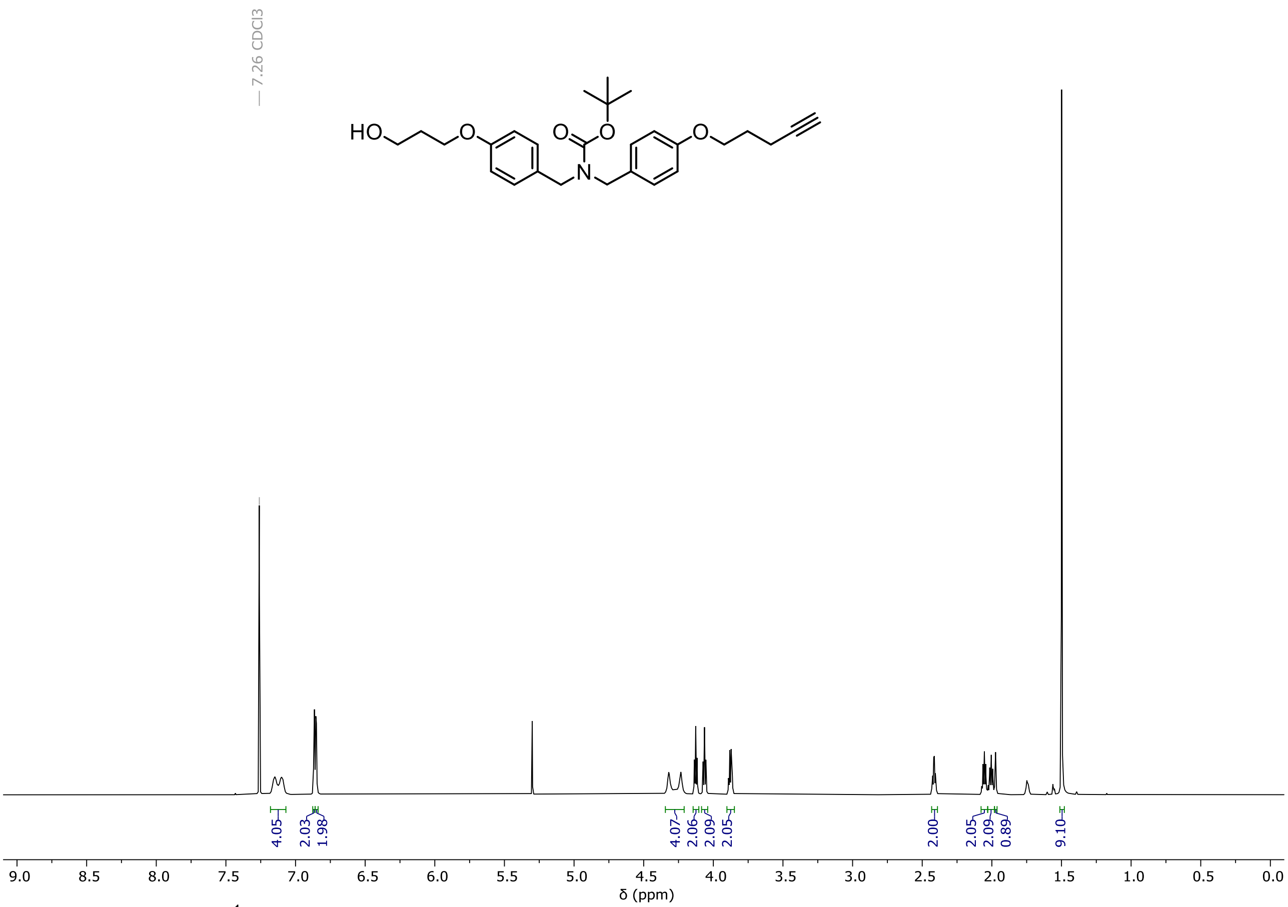


**Spectrum S17** – $^{1}$H NMR (600 MHz, 298 K, Chloroform-*d*) spectrum of **S12**.

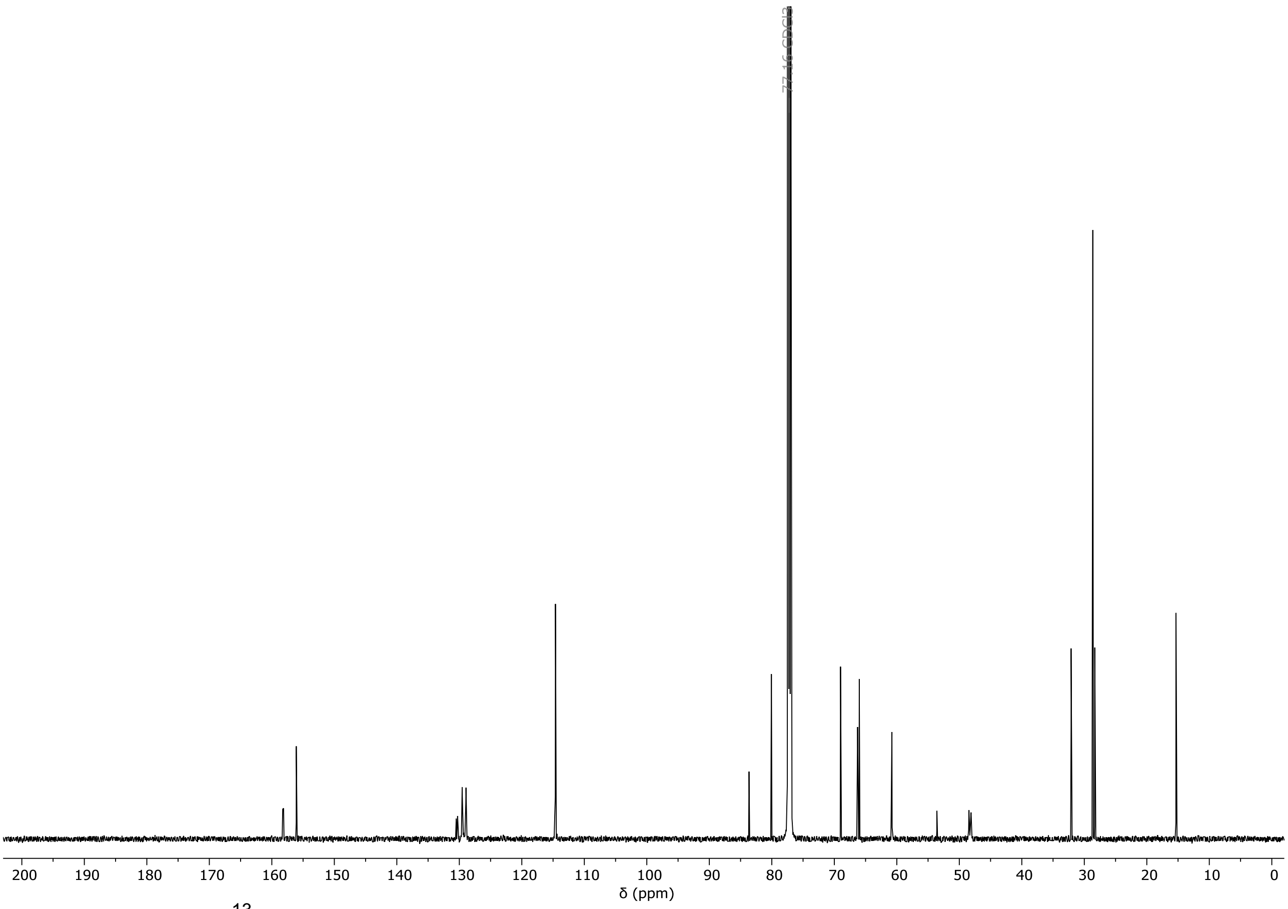


**Spectrum S18** – $^{13}$C NMR (151 MHz, 298 K, Chloroform-*d*) spectrum of **S12**.

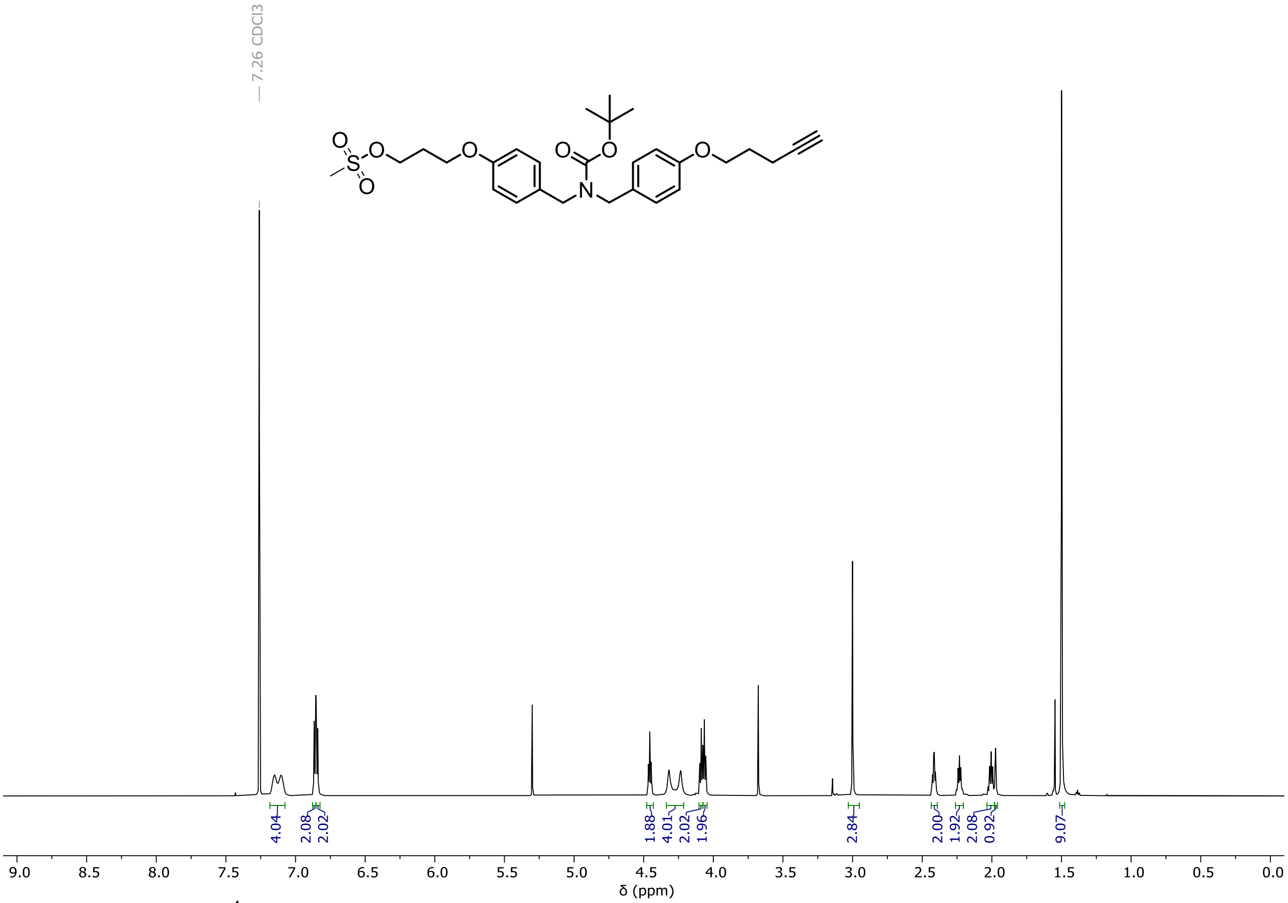


**Spectrum S19** – $^{1}$H NMR (600 MHz, 298 K, Chloroform-*d*) spectrum of **S13**.

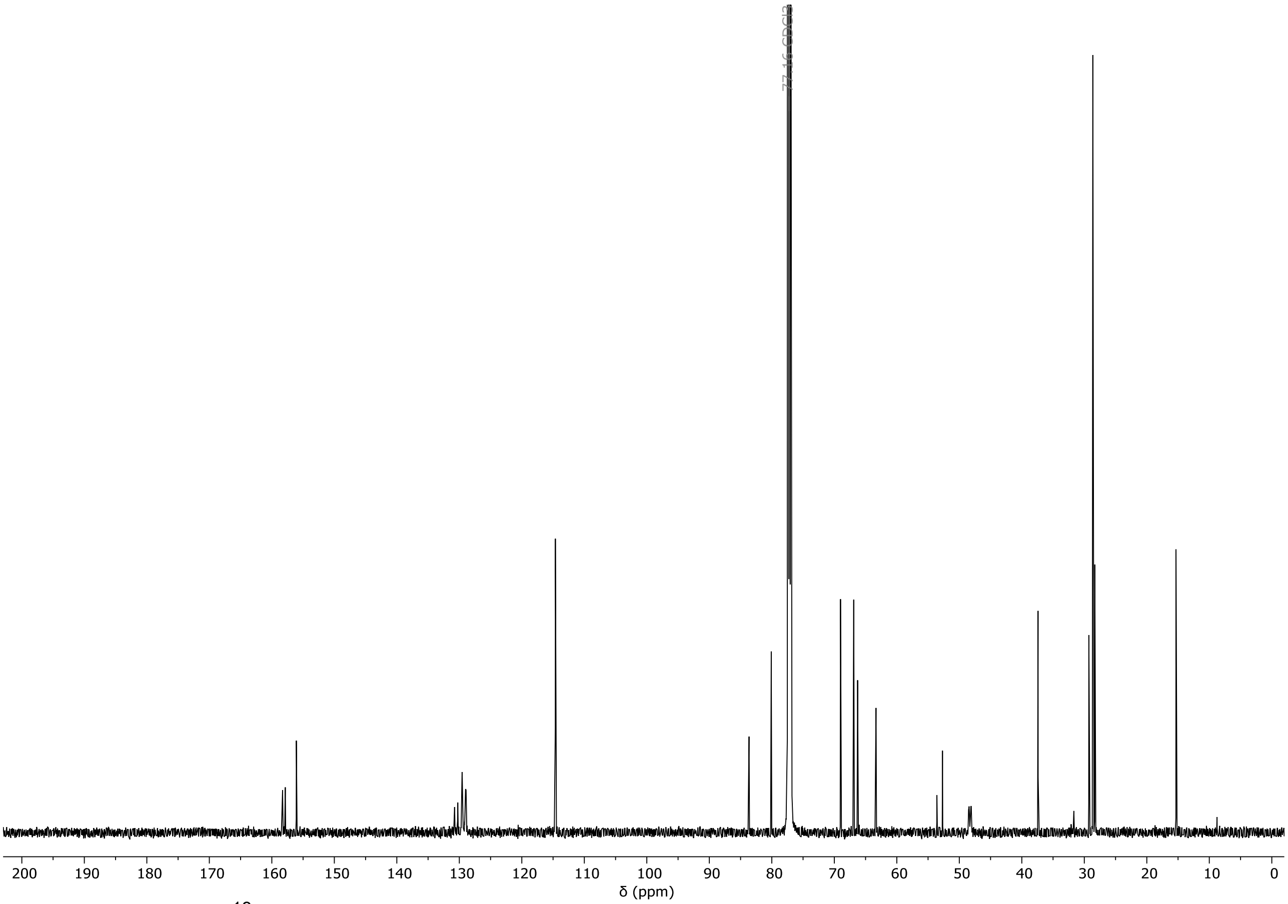


**Spectrum S20** – $^{13}$C NMR (151 MHz, 298 K, Chloroform-*d*) spectrum of **S13**.

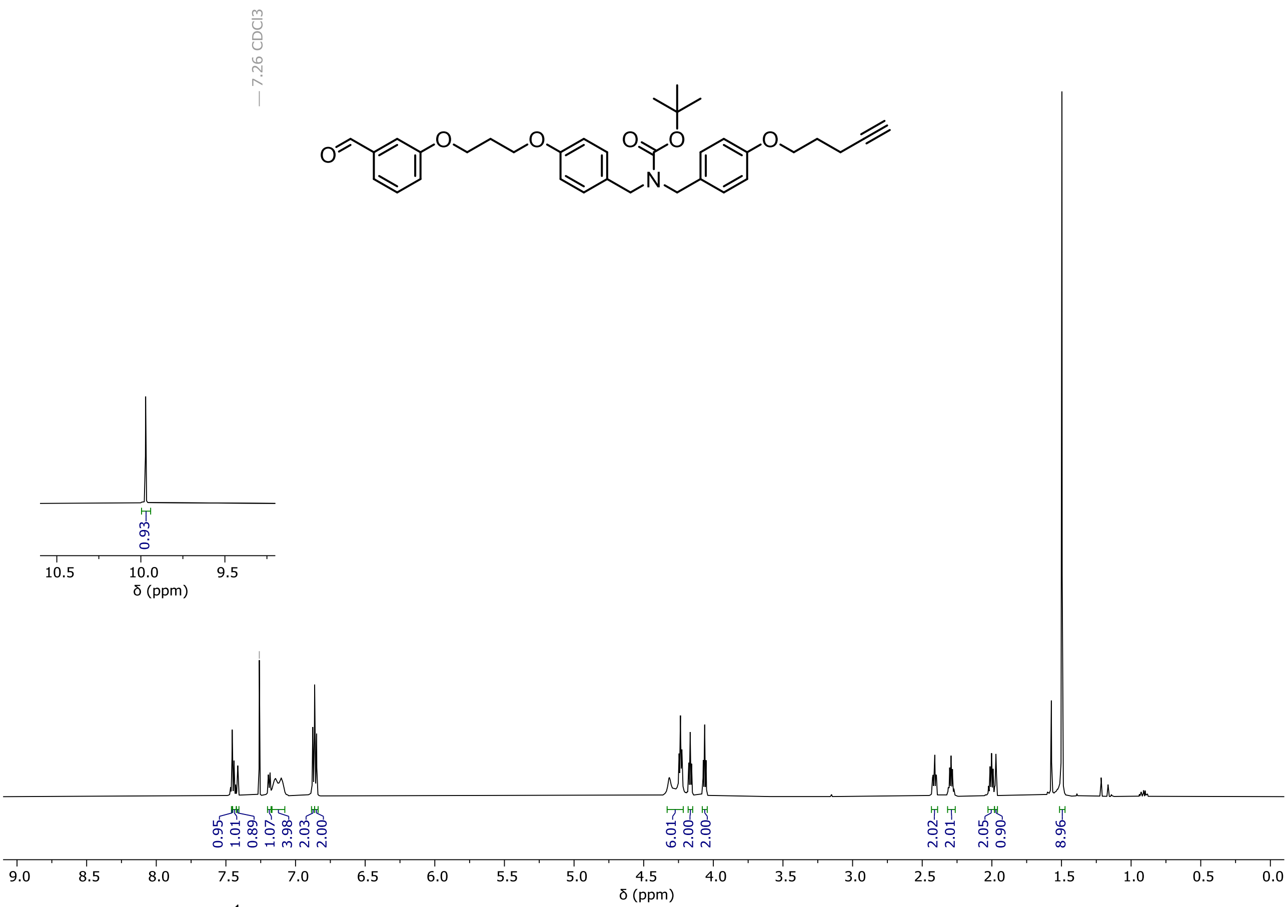


**Spectrum S21** – $^{1}$H NMR (600 MHz, 298 K, Chloroform-*d*) spectrum of **S14**.

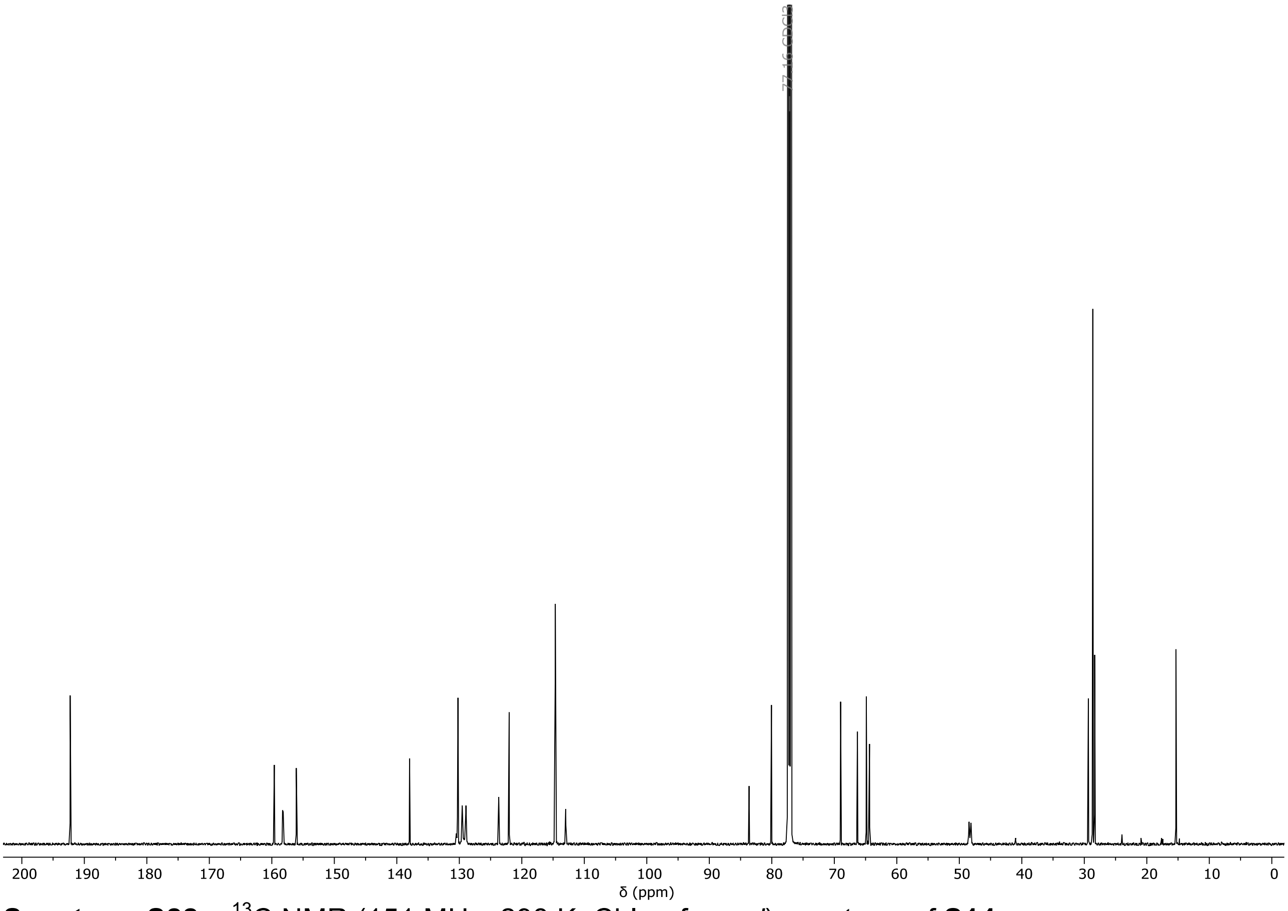


**Spectrum S22** – $^{13}$C NMR (151 MHz, 298 K, Chloroform-*d*) spectrum of **S14**.

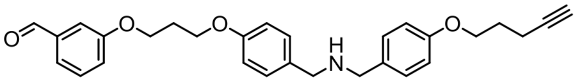

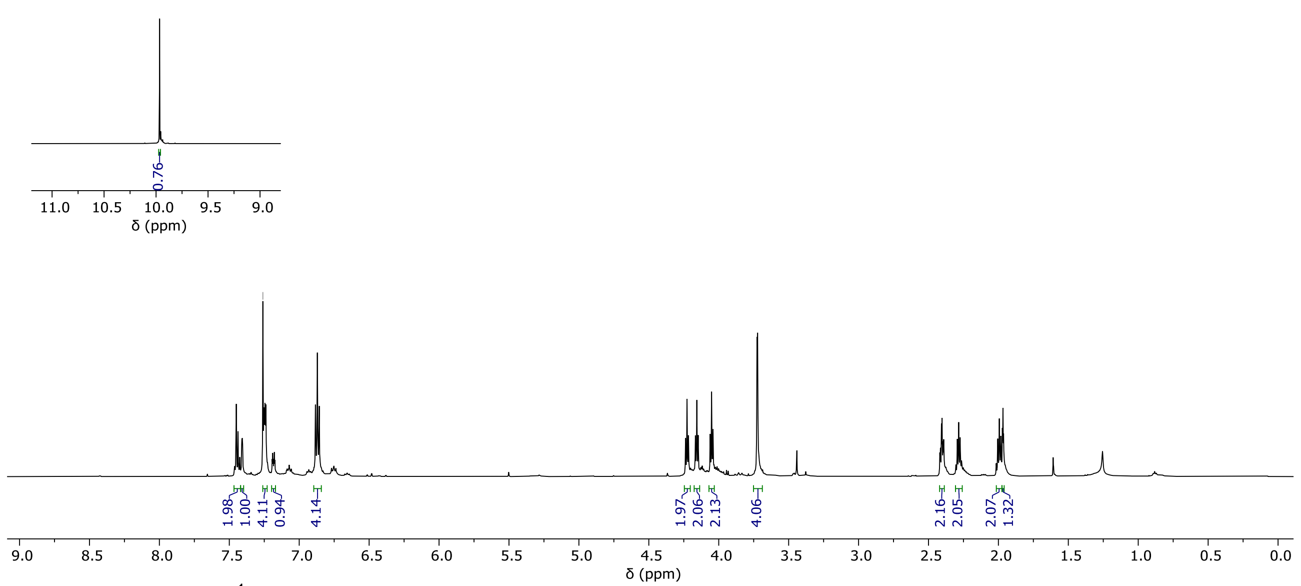


**Spectrum S23** – $^{1}$H NMR (600 MHz, 298 K, Chloroform-*d*) spectrum of **S15**.

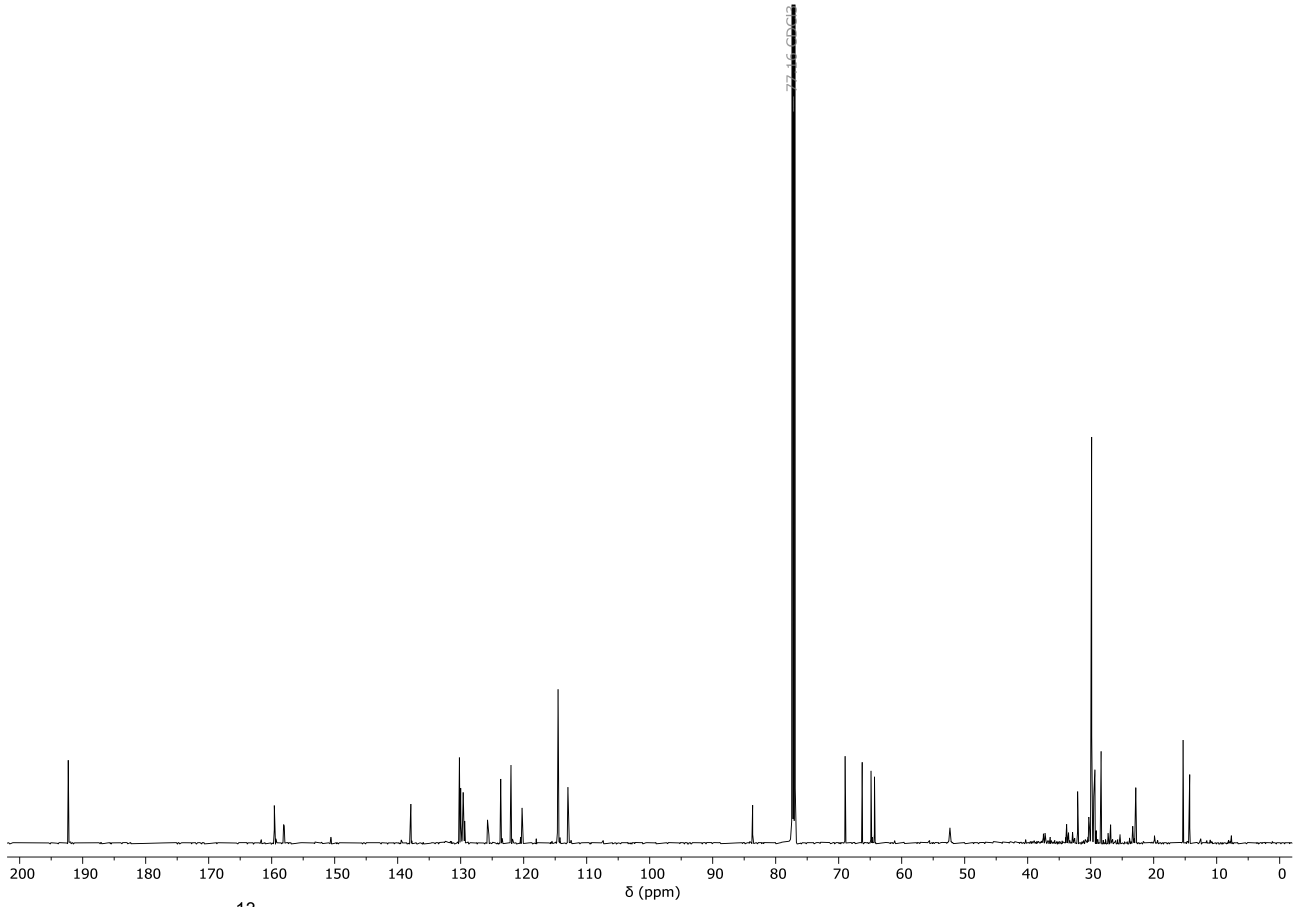


**Spectrum S24** – $^{13}$C NMR (151 MHz, 298 K, Chloroform-*d*) spectrum of **S15**.

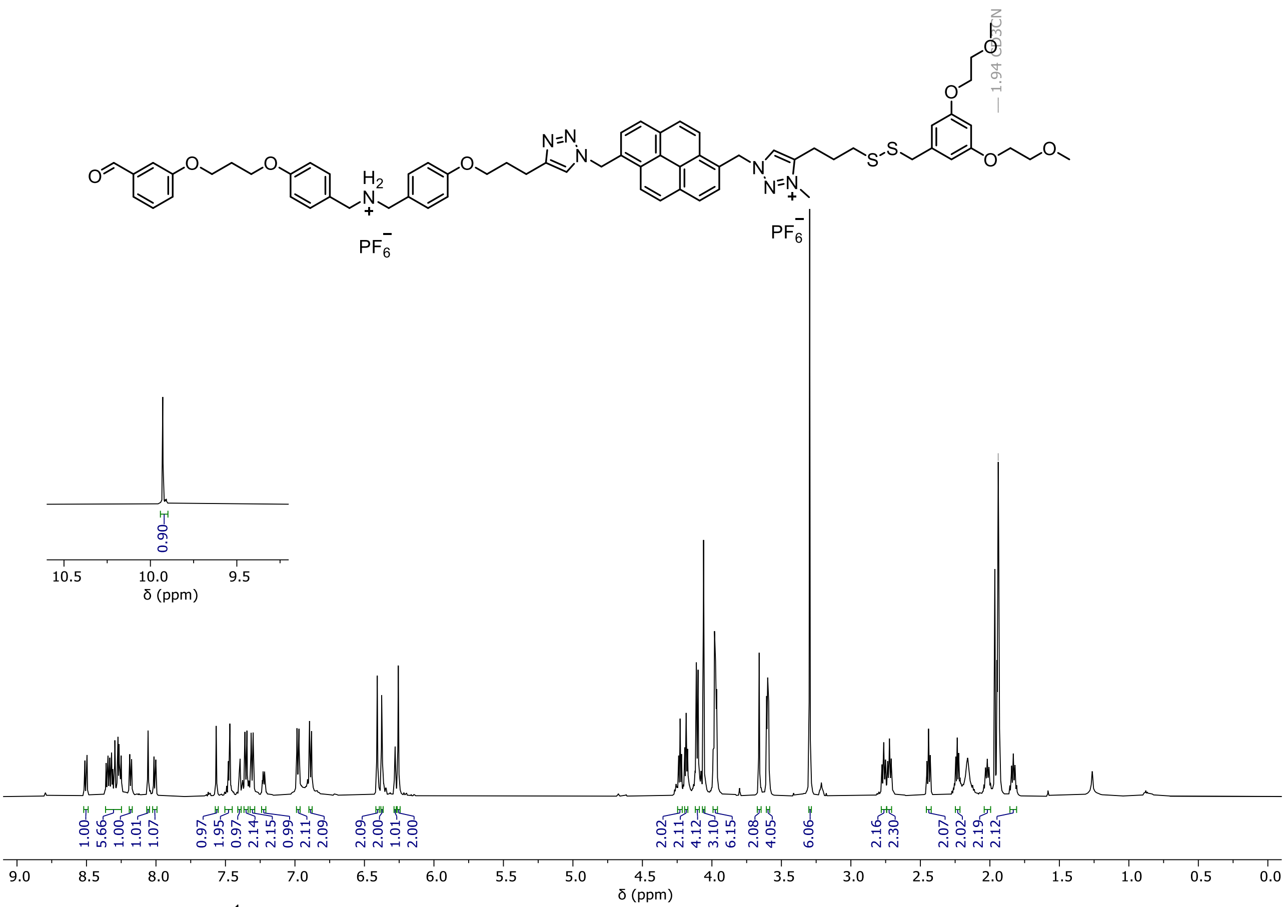


**Spectrum S25** – $^{1}$H NMR (600 MHz, 298 K, Acetonitrile-$d_3$) spectrum of **S16**·H$^{+}$.

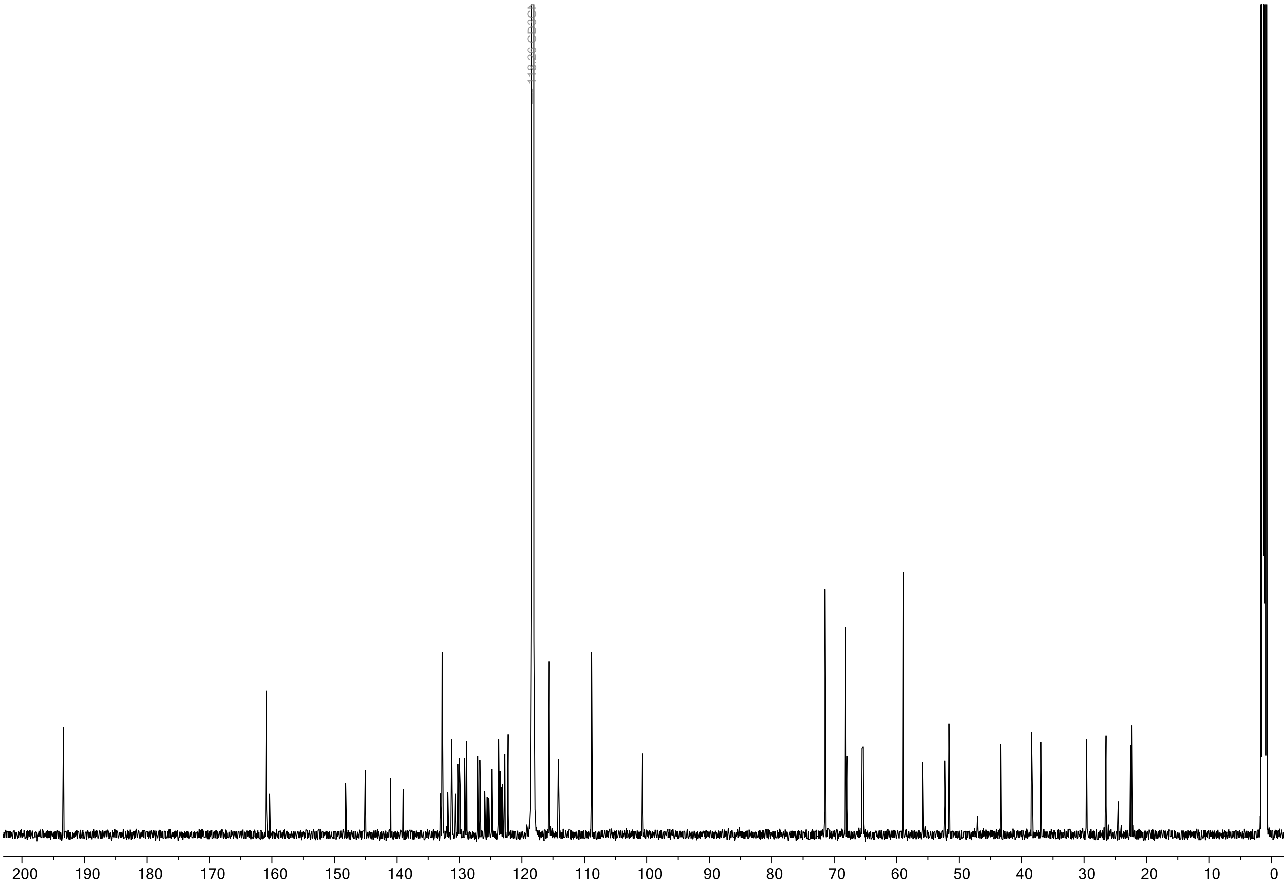


**Spectrum S26** – $^{13}$C NMR (151 MHz, 298 K, Acetonitrile-$d_3$) spectrum of **S16**·H$^{+}$.

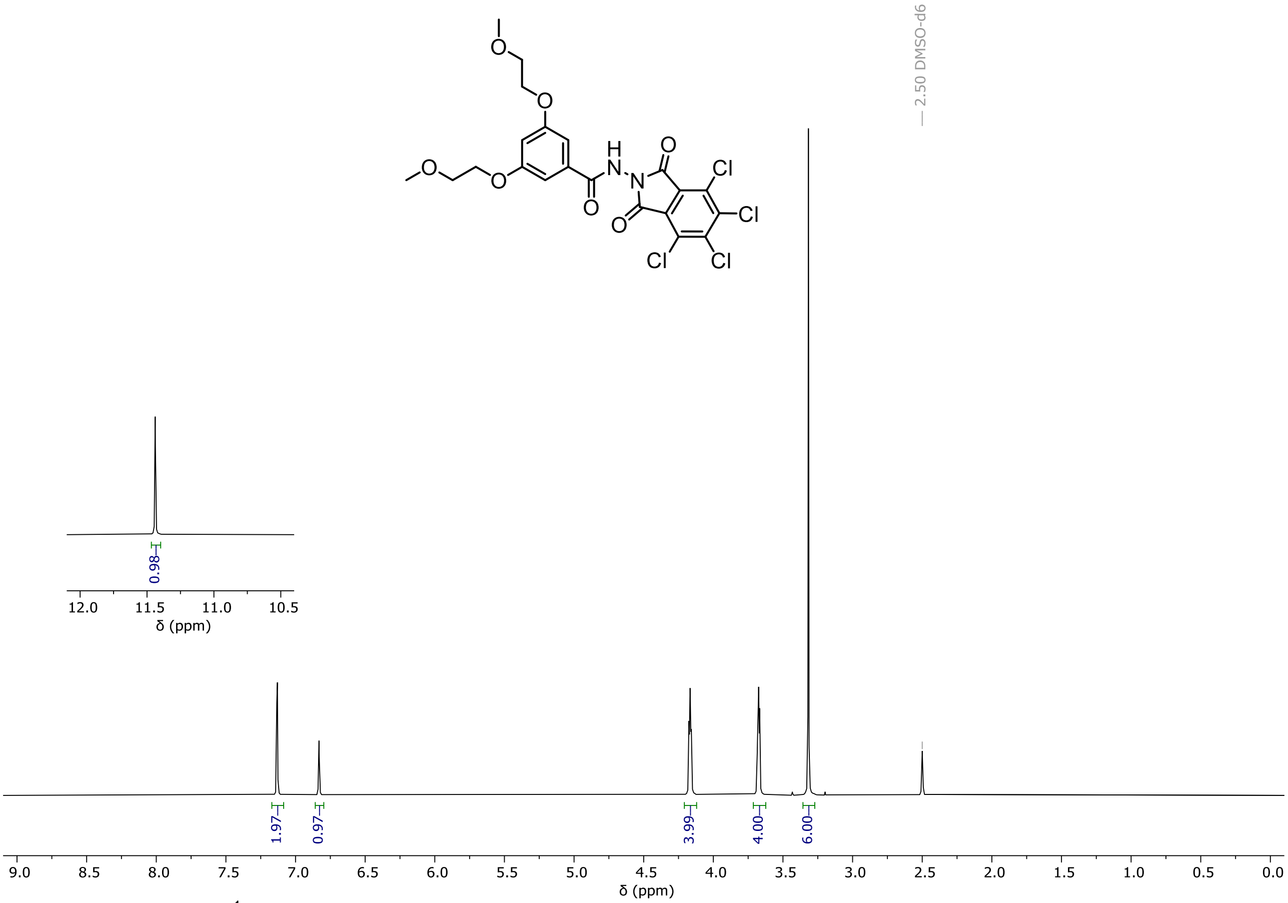


**Spectrum S27** – $^{1}$H NMR (600 MHz, 298 K, DMSO-$d_6$) spectrum of **S18**.

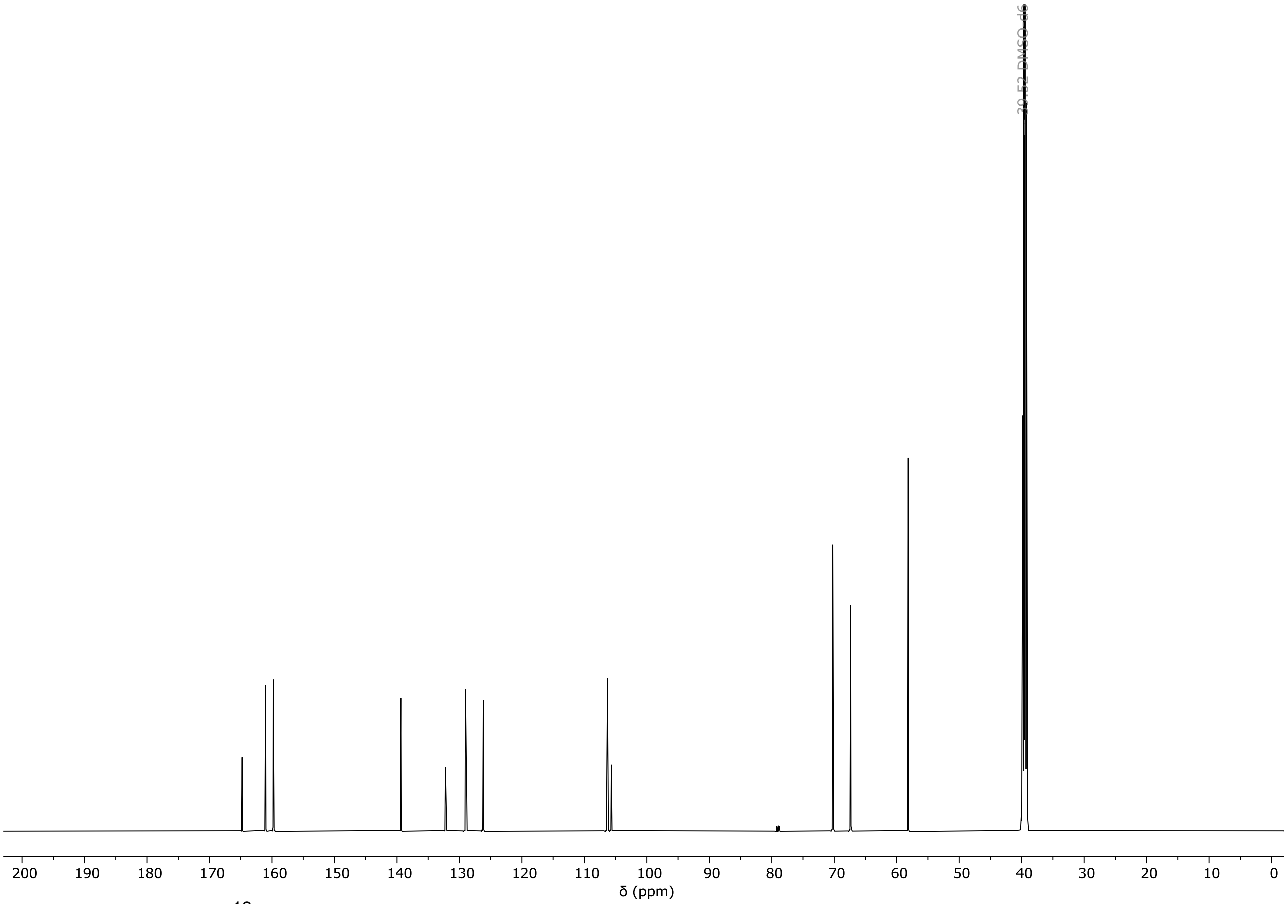


**Spectrum S28** – $^{13}$C NMR (151 MHz, 298 K, DMSO-$d_6$) spectrum of **S18**.

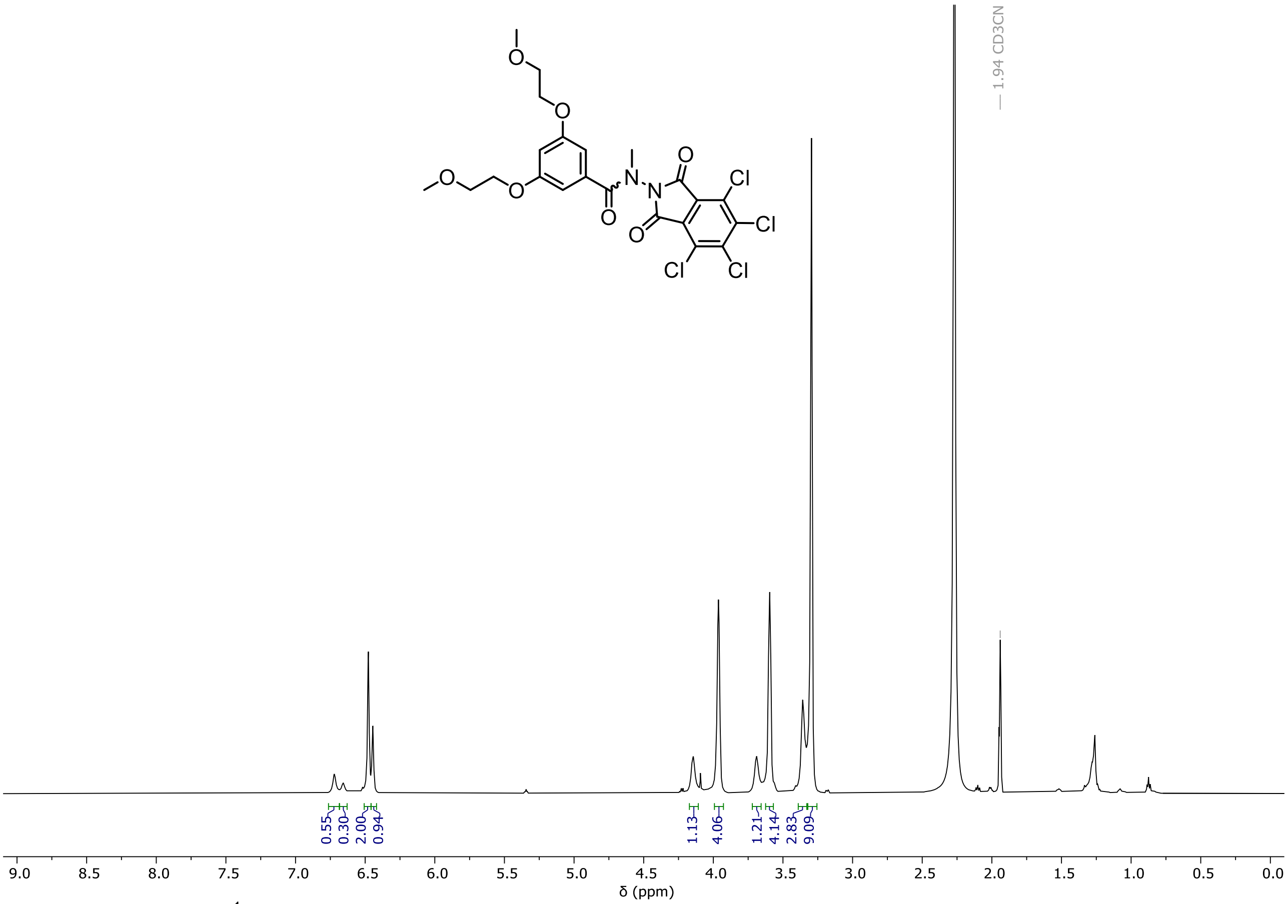


**Spectrum S29** – $^{1}$H NMR (600 MHz, 298 K, Acetonitrile-$d_3$) spectrum of **S19**.

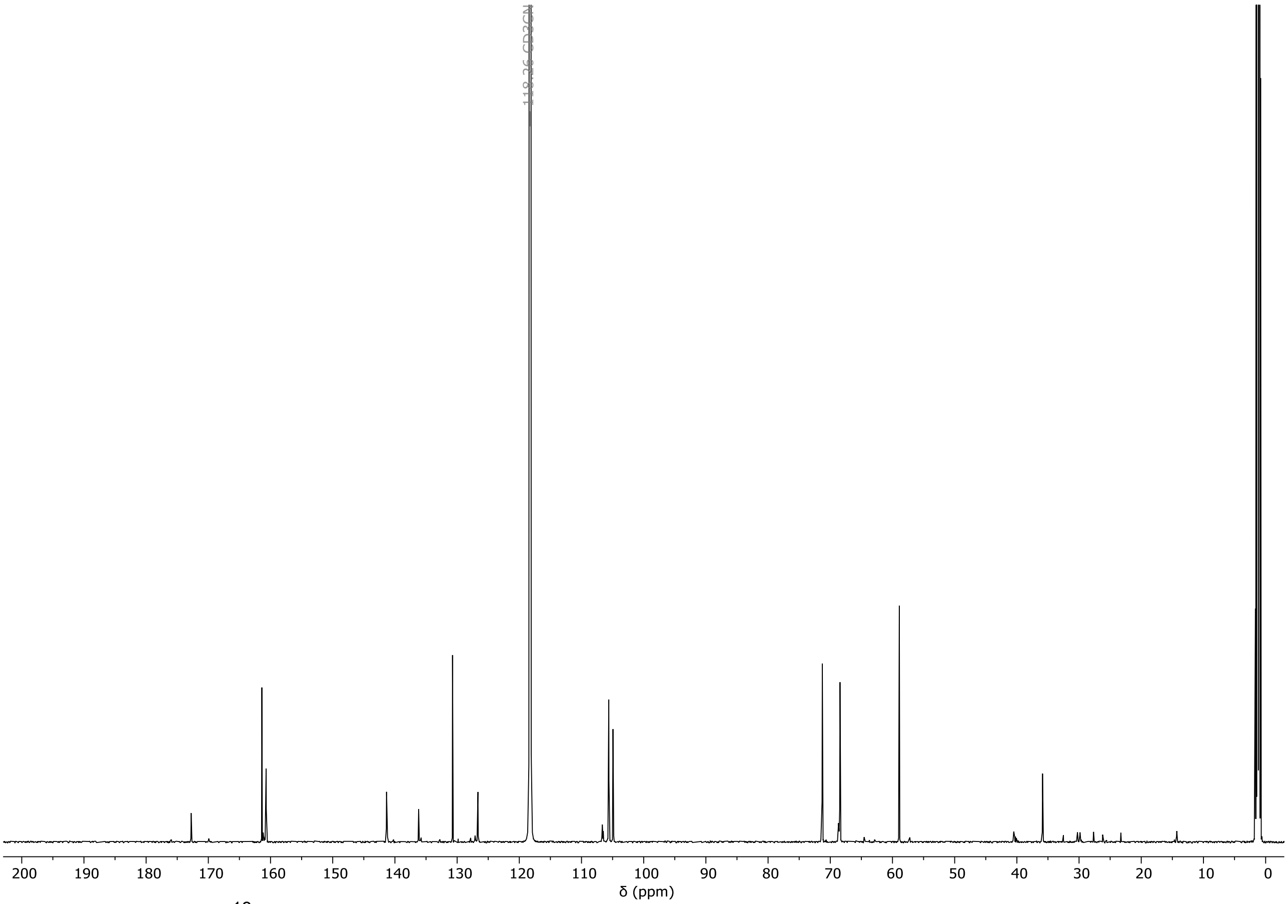


**Spectrum S30** – $^{13}$C NMR (151 MHz, 298 K, Acetonitrile-$d_3$) spectrum of **S19**.

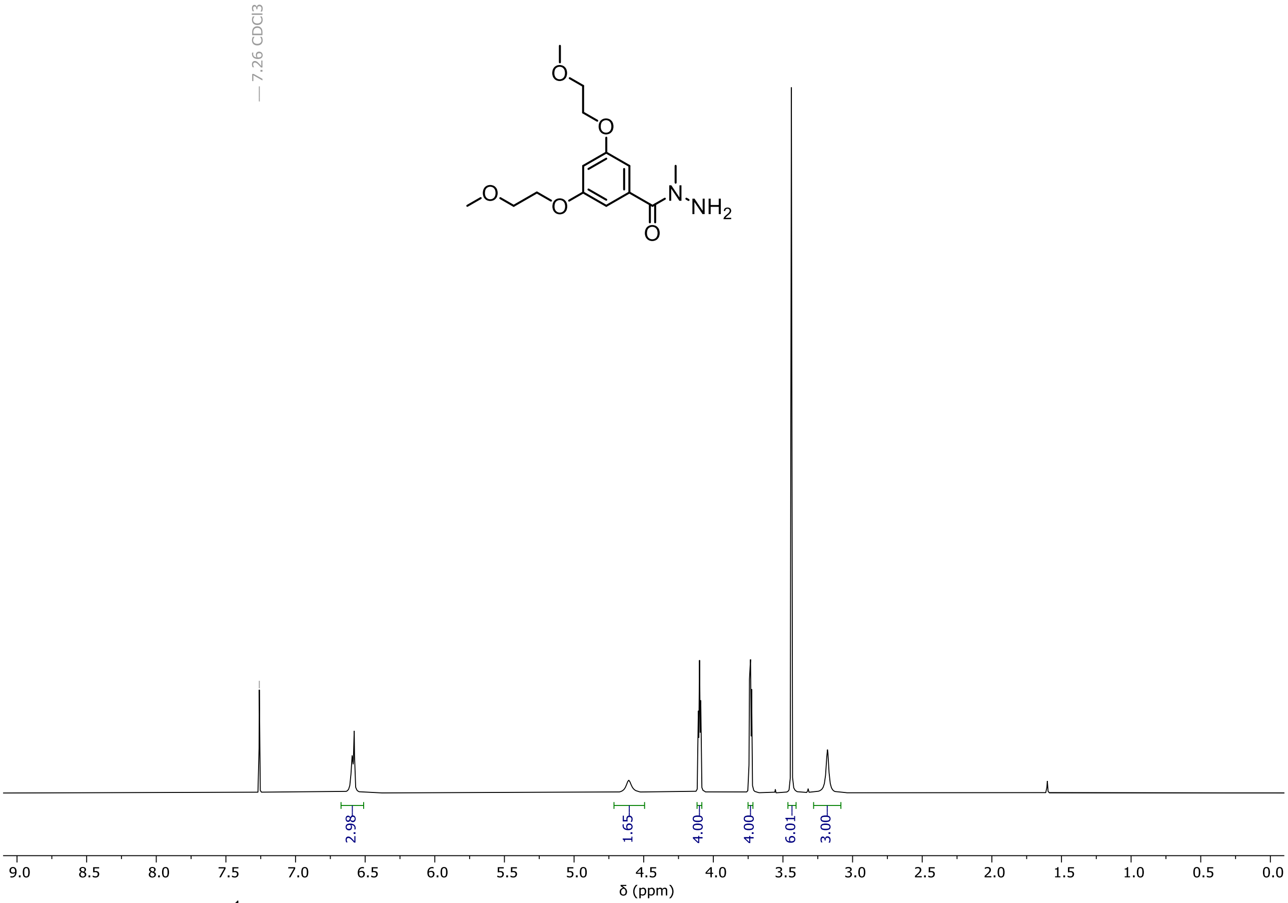


**Spectrum S31** – $^{1}$H NMR (600 MHz, 298 K, Chloroform-*d*) spectrum of **S20**.

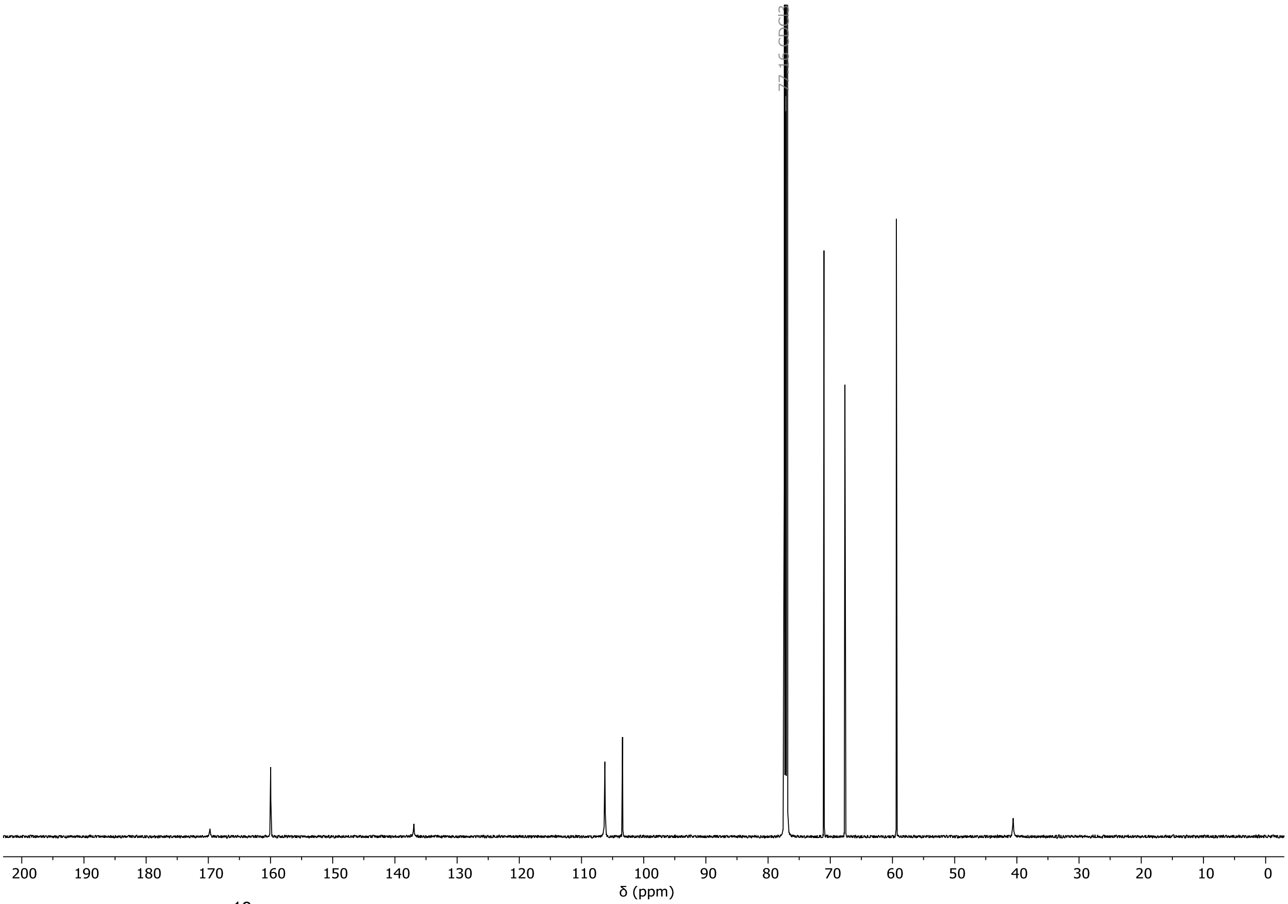


**Spectrum S32** – $^{13}$C NMR (151 MHz, 298 K, Chloroform-*d*) spectrum of **S20**.

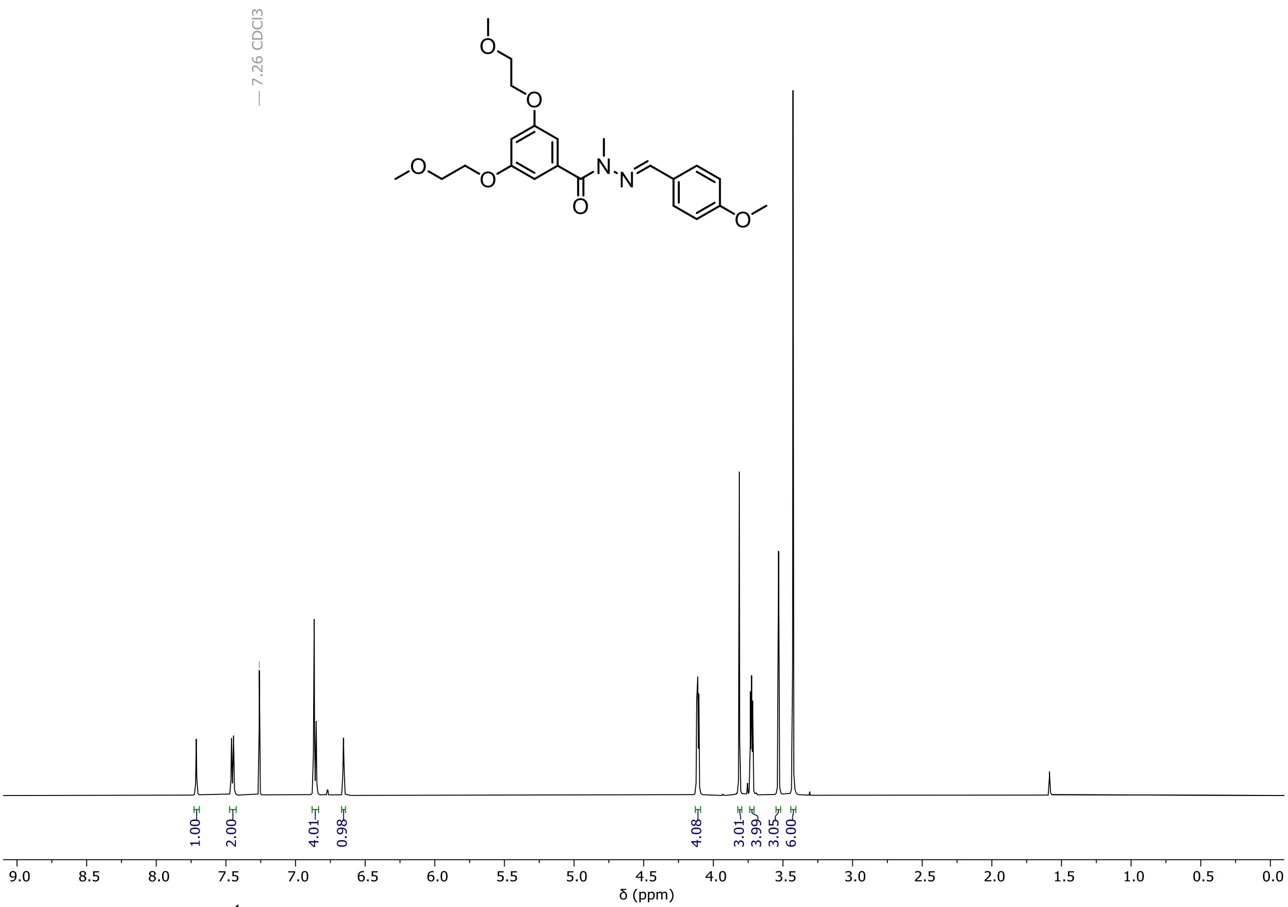


**Spectrum S33** – $^{1}$H NMR (600 MHz, 298 K, Chloroform-*d*) spectrum of **3**.

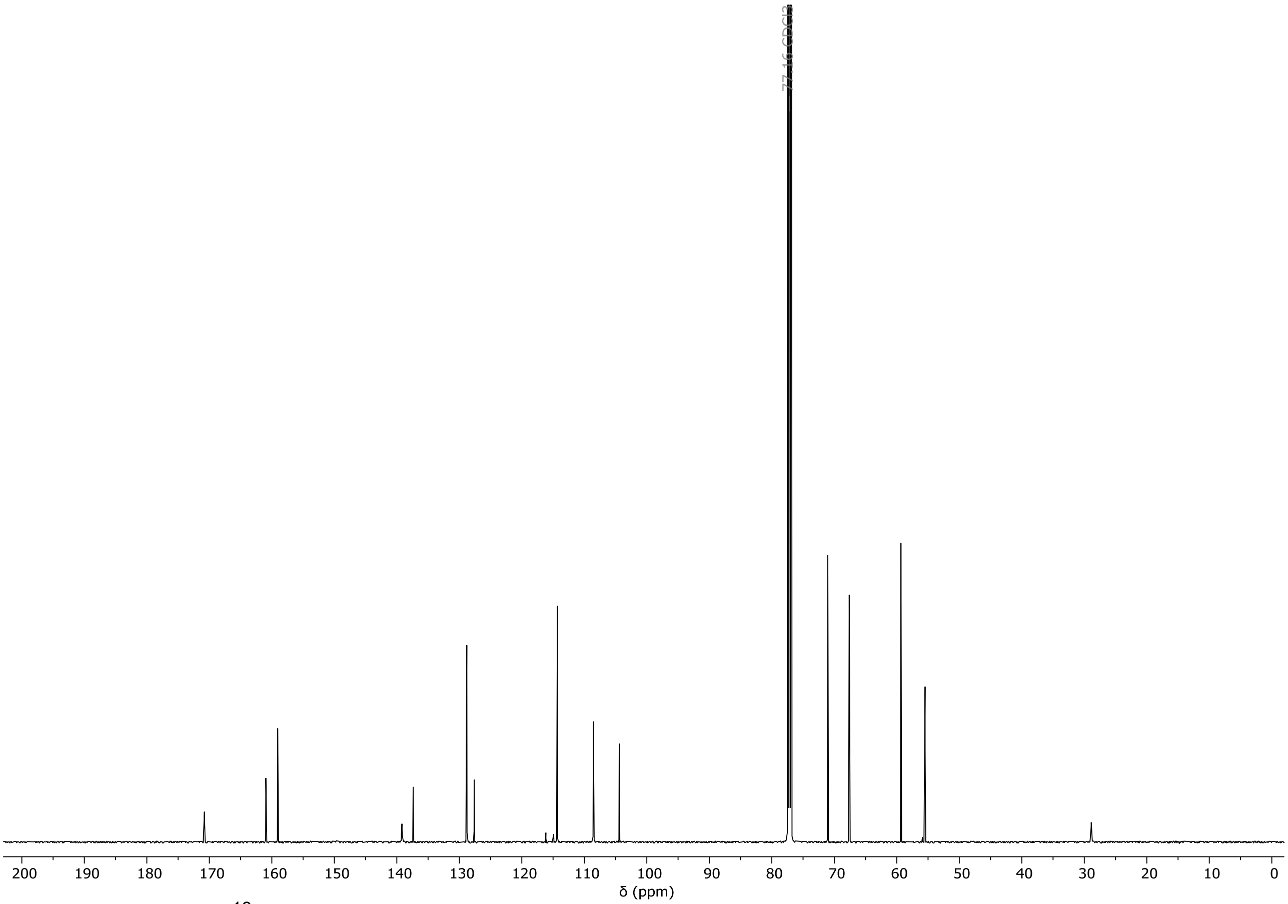


**Spectrum S34** – $^{13}$C NMR (151 MHz, 298 K, Chloroform-*d*) spectrum of **3**.

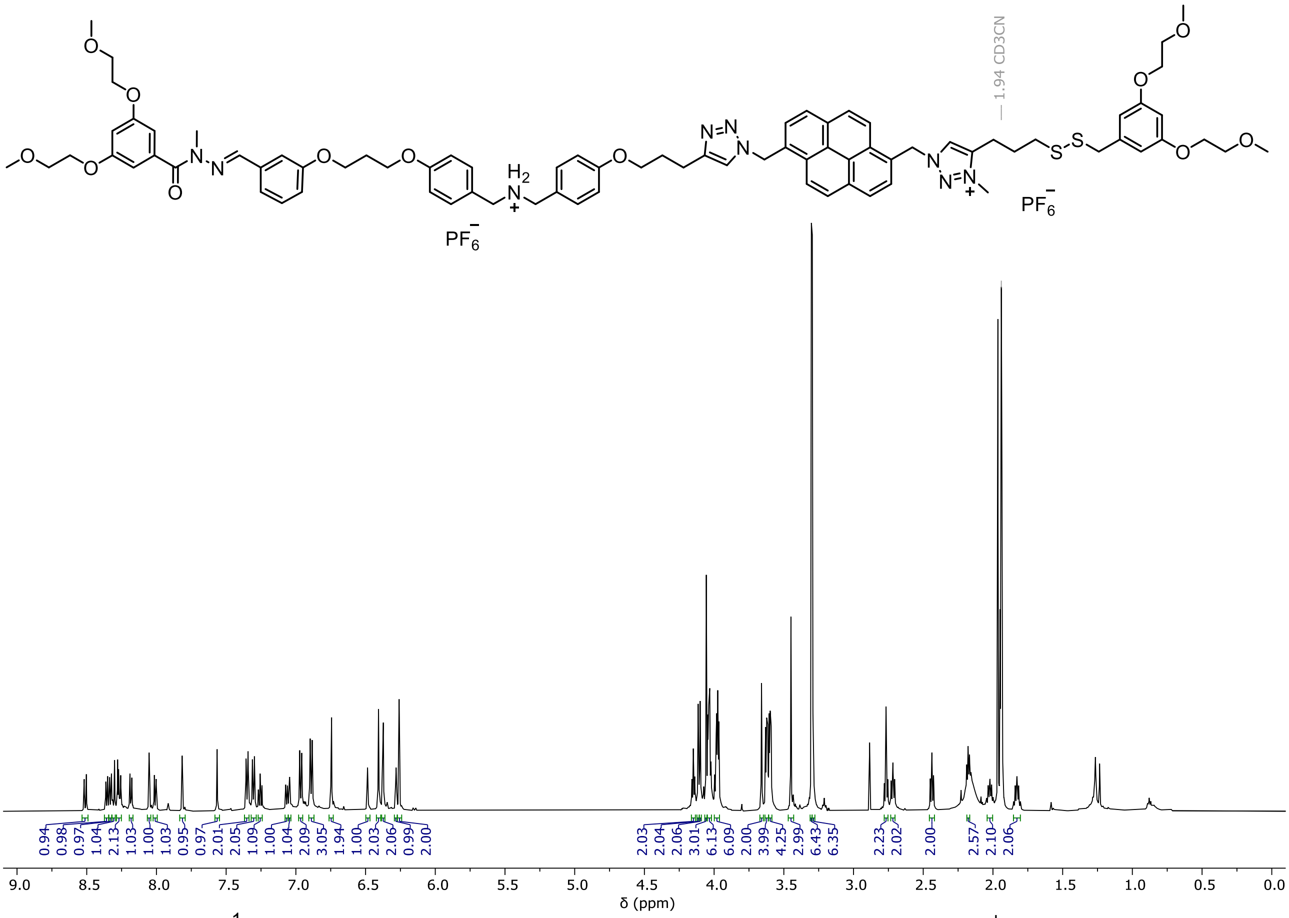


**Spectrum S35** – $^{1}$H NMR (600 MHz, 298 K, Acetonitrile-$d_3$) spectrum of **1**·H$^{+}$.

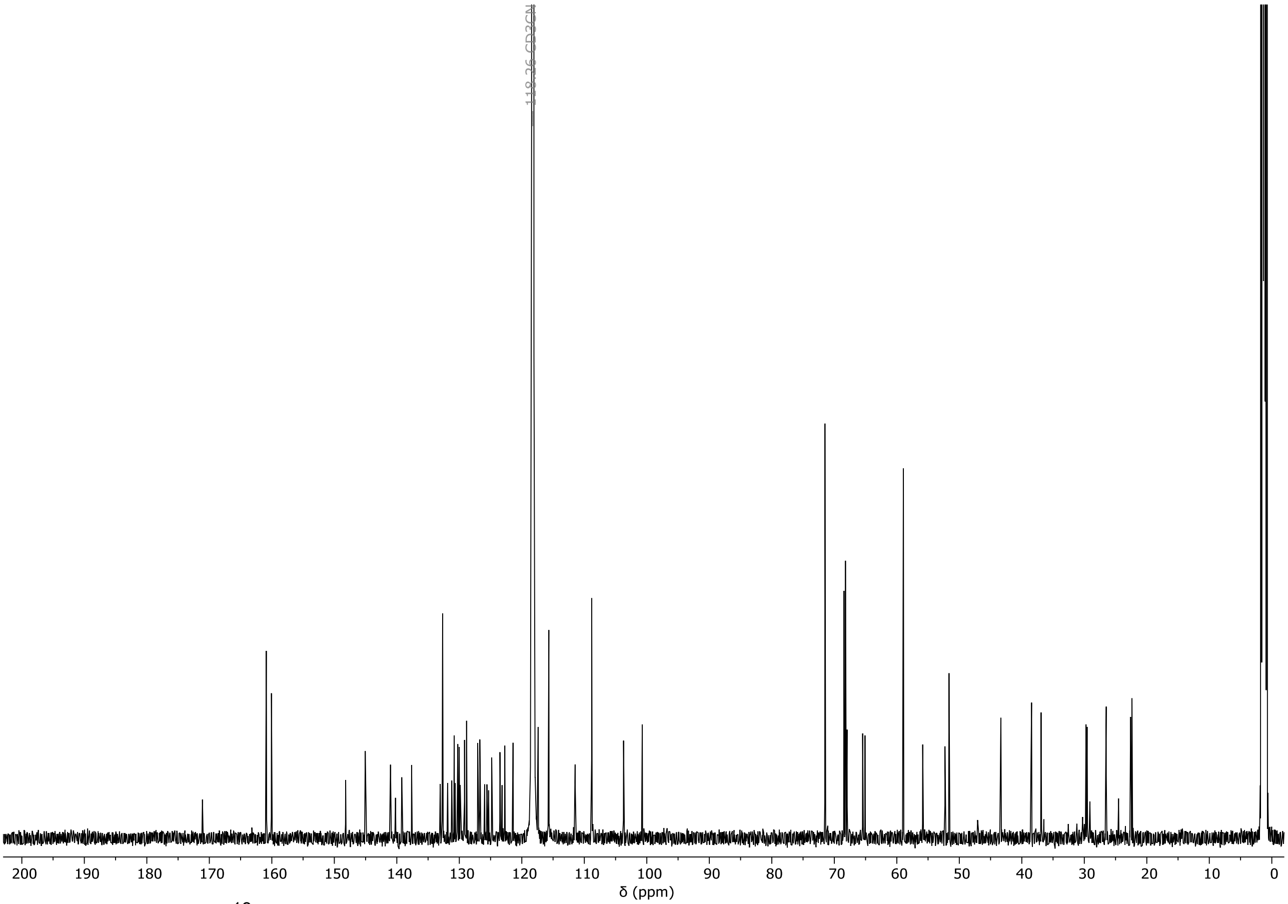


**Spectrum S36** – $^{13}$C NMR (151 MHz, 298 K, Acetonitrile-$d_3$) spectrum of **1**·H$^{+}$.

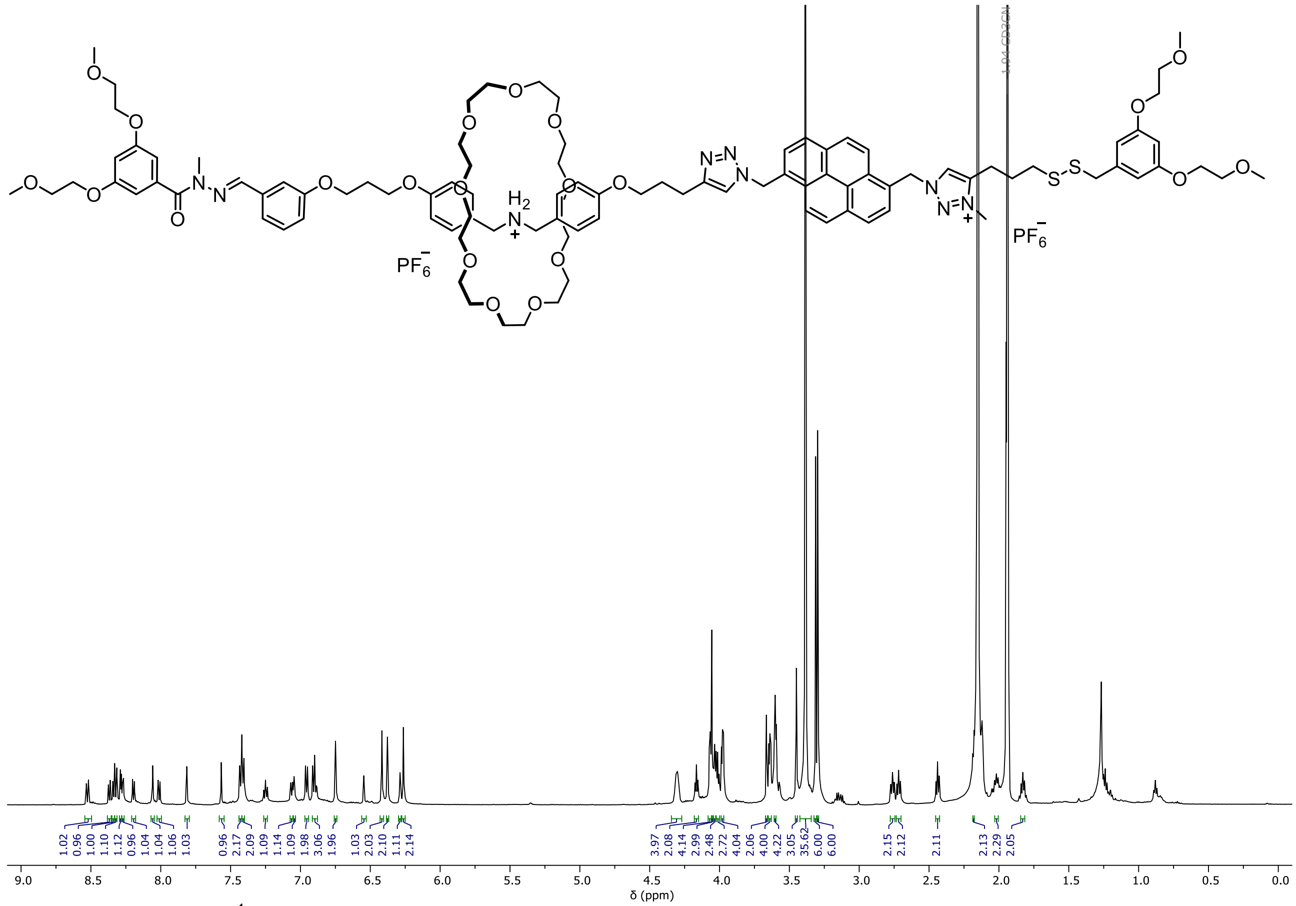


**Spectrum S37** – $^{1}$H NMR (600 MHz, 298 K, Acetonitrile-$d_3$) spectrum of *dba*-**2**·H$^+$.

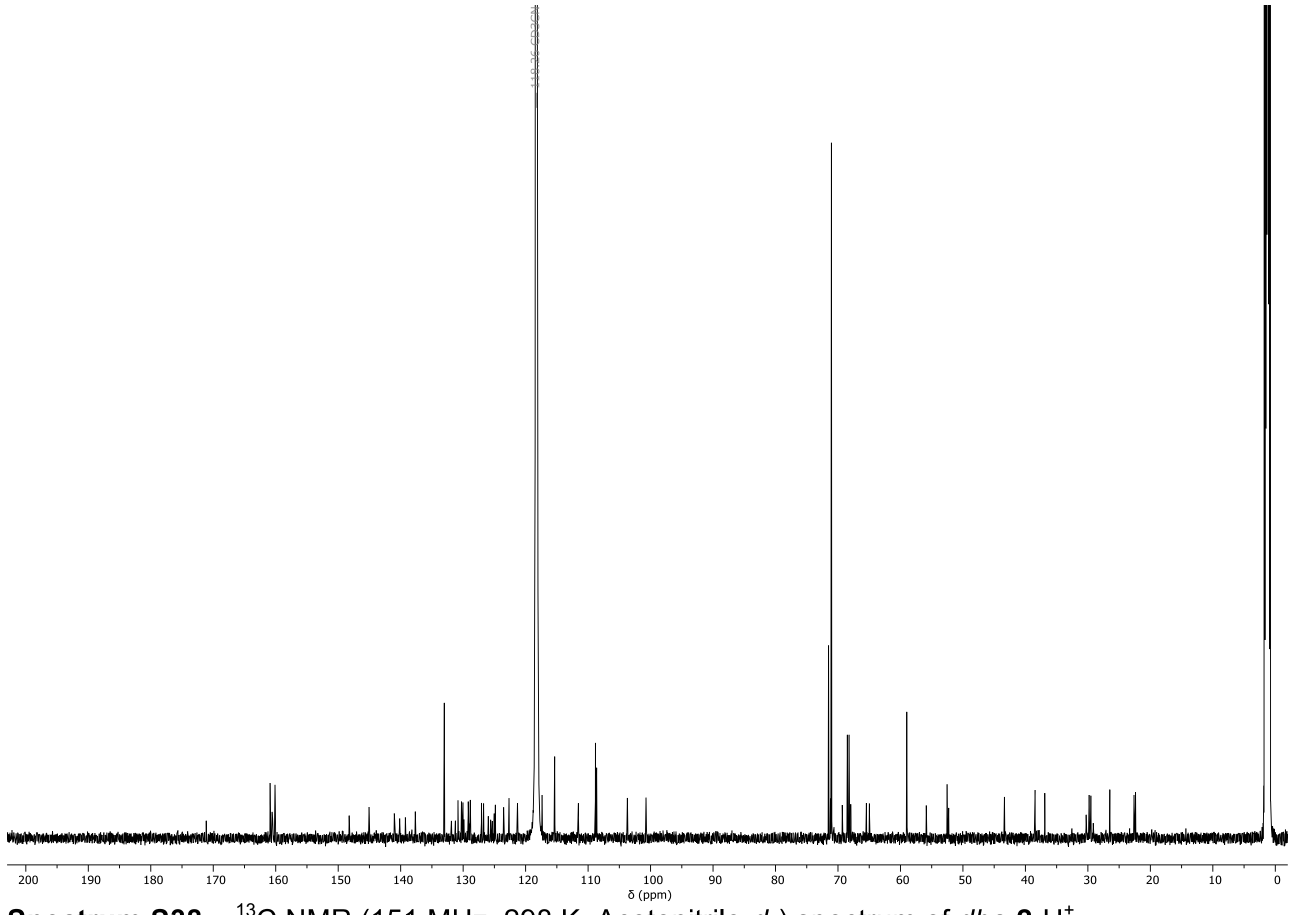


**Spectrum S38** – $^{13}$C NMR (151 MHz, 298 K, Acetonitrile-$d_3$) spectrum of *dba*-**2**·H$^+$.

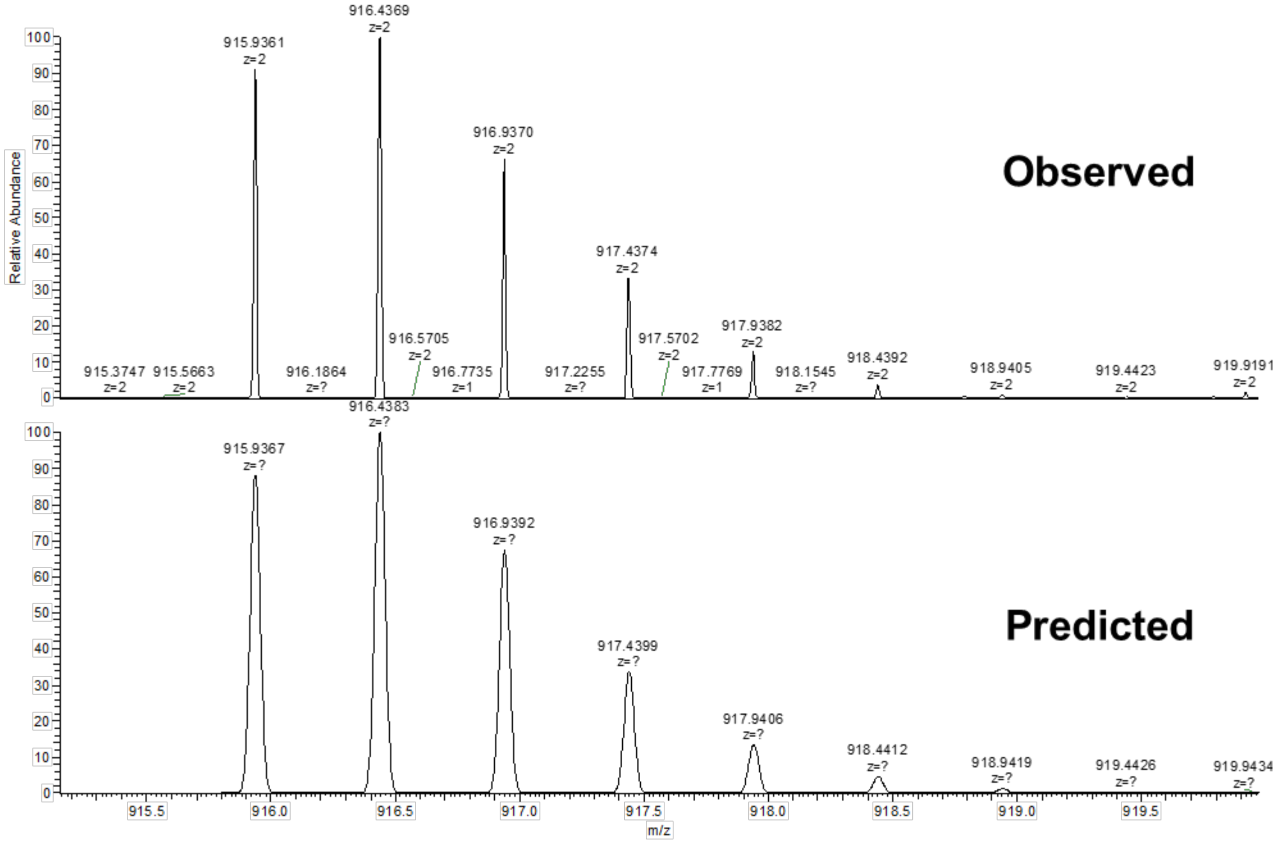


**Spectrum S39** – HRMS (ESI$^+$) spectrum of *dba*-**2**·H$^+$.

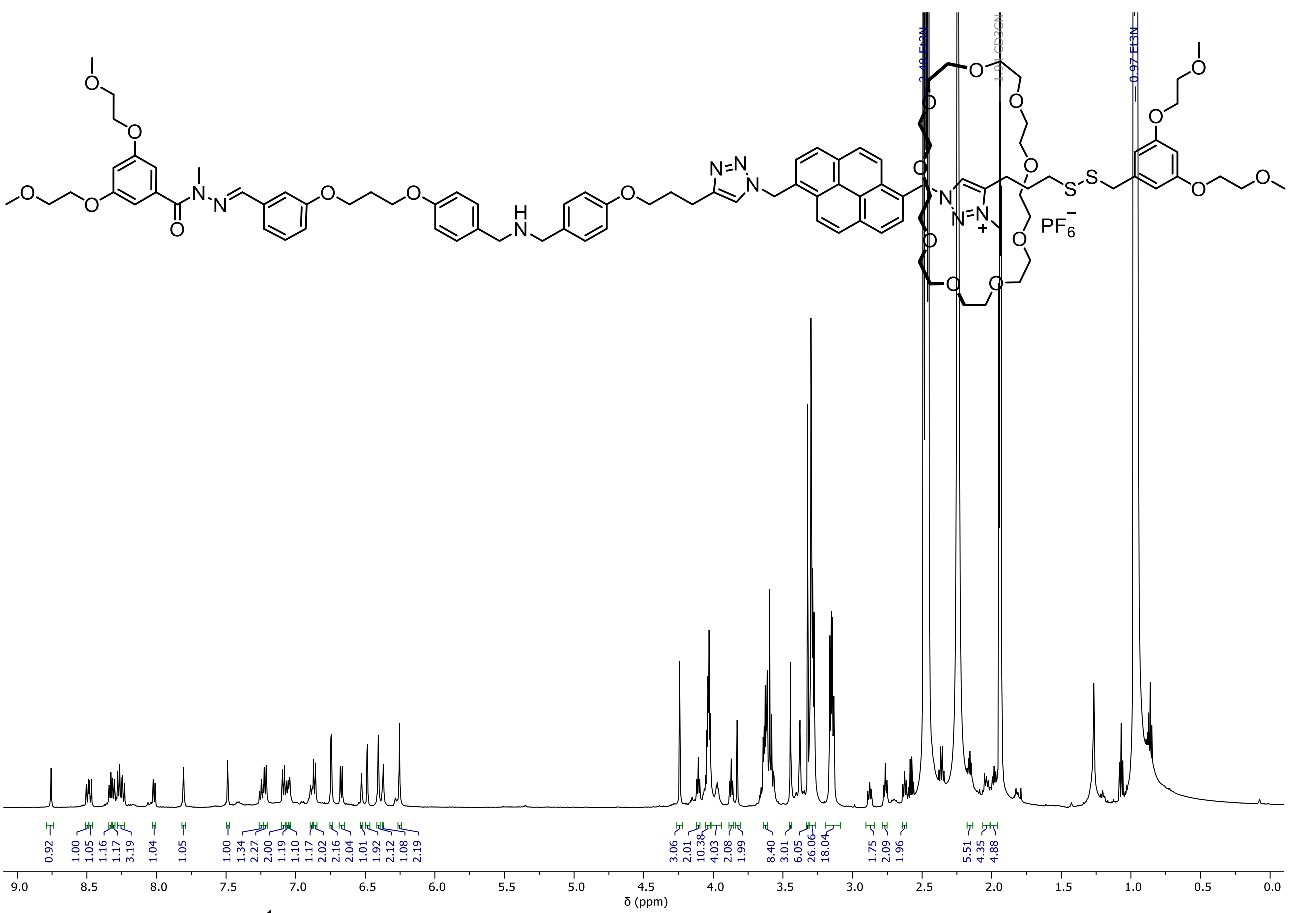


**Spectrum S4039** – $^{1}$H NMR (600 MHz, 298 K, Acetonitrile-$d_3$) spectrum of *mt*-**2**.

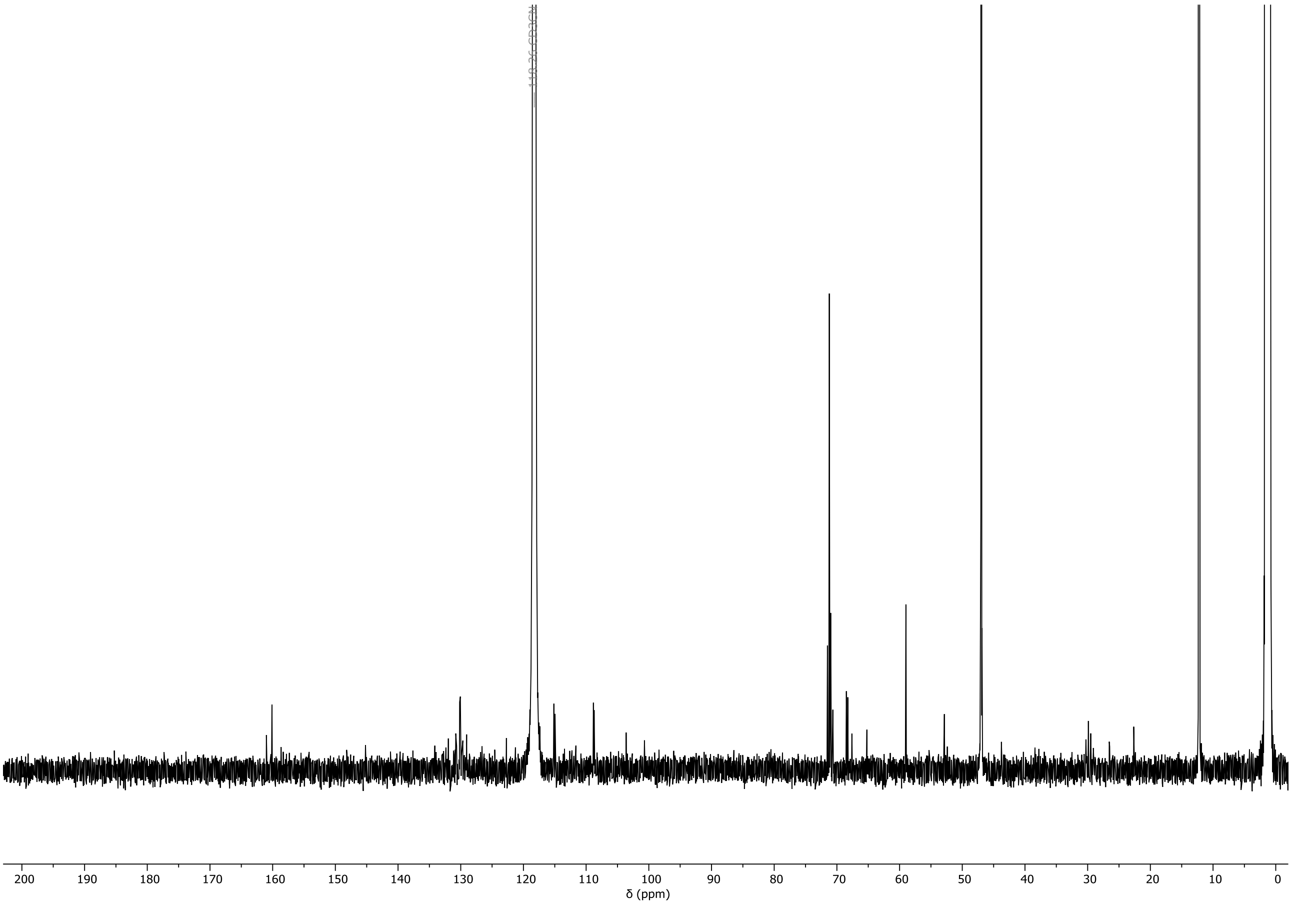


**Spectrum S41** – $^{13}$C NMR (151 MHz, 298 K, Acetonitrile-$d_3$) spectrum of *mt*-**2**.

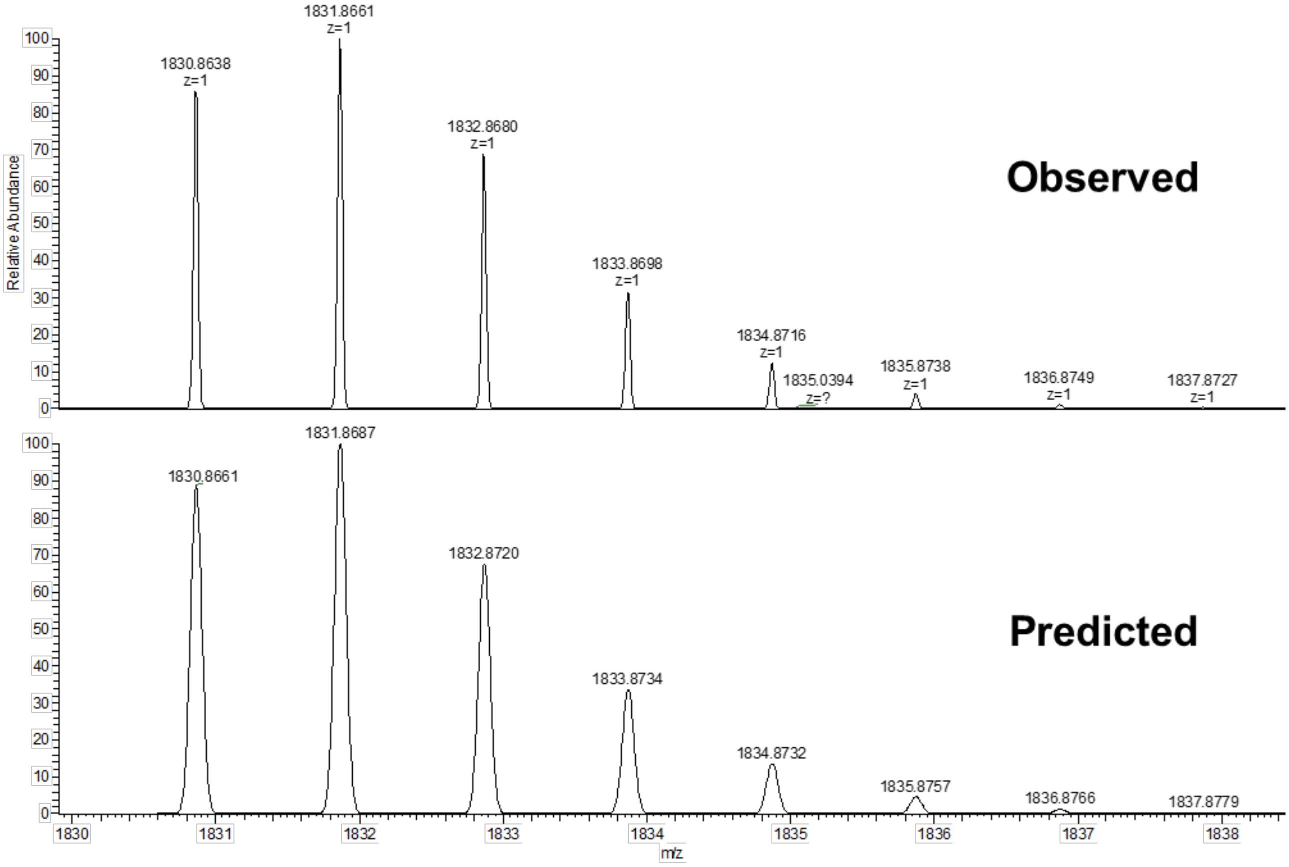


**Spectrum S42** – HRMS (ESI$^+$) spectrum of *mt*-**2**.

# 7 References


1. Shcherbakova, I.; Wermuth, C. G.; Jeannot, F.; Ciapetti, P.; Roques, V.; Heaton, W. L.; Breinholt, J. A.; Conklin, R. A. Pyridazinone Compounds as Calcilytics. U.S. Patent WO 2007044796 A2, April 19, 2007.
2. Driscoll, P.F.; Douglass, E. F. Jr.; Phewluangdee, M.; Soto, E. R.; Cooper C. G.; Macdonald J. C.; Lambert, C. R.; McGimpsey, W. G. Photocurrent generation in noncovalently assembled multilayered thin films. *Langmuir* **2008**, *24*, 9, 5140–5145.
3. Benati, L.; Calestani, G.; Leardini, R.; Minozzi, M.; Nanni, D.; Spagnolo, P.; Strazzari, S. Generation and intramolecular reactivity of acyl radicals from alkynylthiol esters under reducing tin-free conditions. *Org. Lett.* **2003**, *5*, 8, 1313–1316.
4. Zheng, H.; Li, Y.; Zhou, C.; Li, Y.; Yang, W.; Zhou, W.; Zuo, Z.; Liu, H. Synthesis of a [2]Rotaxane Incorporating a “Magic Sulfur Ring” by the Thiol-Ene Click Reaction. *Chem. Eur. J.* **2011**,*17*, 2160–2167.
5. Weiss, H.; Reichel, J.; Görls, H.; Schneider, K. R. A.; Micheel, M.; Pröhl, M.; Gottschaldt, M.; Dietzek, B.; Weigand, W. Curcuminoid–$BF_2$ complexes: Synthesis, fluorescence and optimization of $BF_2$ group cleavage. *Beilstein J. Org. Chem.* **2017**, *13*, 2264–2272.
6. Thomas, D.; Tetlow, D. J.; Ren, Y.; Kassem, S.; Karaca, U.; Leigh, D. A. Pumping between phases with a pulsed-fuel molecular ratchet. *Nat. Nanotechnol.* **2022**, *17*, 701–707.
7. Borodin, O.; Shchukin, Y.; Robertson, C. C.; Richter, S.; von Delius, M. Self-assembly of stimuli-responsive [2]rotaxanes by amidinium exchange. *J. Am. Chem. Soc.* **2021**, *143*, 16448–16457.
